# Coarse-grained Martini 3 model for collagen fibrils

**DOI:** 10.1016/j.bpj.2025.10.012

**Published:** 2025-10-10

**Authors:** Matthias Brosz, Johanna Buck, Fabian Grünewald, Debora Monego, Jaewoon Jung, Yuji Sugita, Camilo Aponte-Santamaría, Frauke Gräter

**Affiliations:** 1 Max Planck Institute for Polymer Research, Ackermannweg 10, 55128 Mainz, Germany; 2 Heidelberg Institute for Theoretical Studies, Am Schloss-Wolfsbrunnenweg 35, 69117 Heidelberg, Germany; 3 Institute for Scientific Computing, Heidelberg University, Im Neuenheimer Feld 205, 69120 Heidelberg, Germany; 4 Computational Biophysics Research Team, RIKEN Center for Computational Science, Kobe 650-0047, Yogo, Japan; 5 Theoretical Molecular Science Laboratory, RIKEN Pioneering Research Institute, Wako 351-0198, Japan

## Abstract

Collagen is a prevalent protein in the Animalia kingdom, especially in mammals. It is abundant in all connective tissue such as bone or ligaments, and thus, it is subjected to substantial mechanical forces. Cross-links play an essential role for the structural and mechanical integrity of collagen, determining its stiffness and rigidity. Until now, studies on collagen including cross-links have either been confined to fully atomistic simulations, which are computationally intensive and restrict the accessible time and length scales, or to coarse-grained descriptions that do not resolve the force response on a residue level and therefore do not consider the triple helical structure and the connectivity of cross-links. To bridge this gap, we report on the development and validation of a computational model based on the Martini 3 coarse-grained force field, in which we parametrized the fibrillar collagen structure including cross-links. We validated the model, through extensive equilibrium and nonequilibrium molecular dynamics simulations, against experimental properties and all-atom simulations. Because the type and distribution of cross-links vary with aging, we expect that this collagen model can be employed to provide insights into age-related changes in tissue mechanics and guide the development of biomimetic materials.

## Significance

Collagen’s distinctive triple helical structure and exceptional mechanical stability make it a central component of connective tissues. Understanding the role of covalent cross-links in stabilizing collagen fibrils remains a key challenge due to limitations in existing computational models, which often compromise on either resolution or system size. Here, we present a coarse-grained computational model of collagen fibrils, enabling detailed investigations of the role of cross-links and their impact on fibril properties.

## Introduction

Collagen-based fibrillar structures serve dual critical roles in animals: they act as force-transmitting proteins between muscles and bones and, in this context, bear extreme mechanical loads—up to 90 MPa in a stretched Achilles tendon (1,2,3). As one of the most abundant proteins in the human body, collagen is widely used as a biomaterial in tissue engineering to treat burns and wounds, to serve as a biological scaffold for bone and cartilage regeneration, and to promote cell integration and proliferation in cardiovascular applications (4). The properties of collagen can be precisely controlled through its composition and cross-linking. This allows collagen-based scaffolds to be engineered to meet the diverse mechanical and structural requirements across different applications (5,6,7).

Collagen, which exists in several types, is most commonly found as collagen type I, consisting of stiff triple helices with persistence lengths ranging between 11 and 15 nm or between 65 and 180 nm, as measured through atomic force microscopy or dynamic light scattering, respectively (8, 9). Cross-links interconnect the triple helices, forming a substructure called a fibril. At this scale, collagen exhibits an alternating pattern of high- and low-density regions, referred to as overlap and gap zones. Each unit of gap and overlap region is a D-band and has a length of 67 nm. Several fibrils assemble together to form collagen fibers.

To uncover collagen’s dynamic and mechanical properties across this hierarchical structure, computational simulation methods ranging from the atomistic to the mesoscopic scale have been employed. All-atom (AA) molecular dynamics (MD) simulations, on the one hand, have investigated both single collagen molecules and 67-nm-long fibrillar structures, revealing mechanisms of triple helical folding and stress distribution within cross-linked fibrils, respectively (10,11,12,13,14,15). However, they have been limited to single D-band lengths, preventing analysis of full-length (∼335 nm) microfibrils under force. To bridge the gap between molecular and fibrillar scales, Buehler et al. and others developed a mesoscopic model from AA-MD simulations of a 84-nm-long collagen molecule to investigate how cross-linking affects the stress-strain response at the fibril level (16). Vaughan further extended this model to characterize the influence of mineralization on collagen’s response to mechanical load (17). Although this mesoscopic model qualitatively reproduces collagen’s overall strength, it lacks a clear chemical and thermodynamic distinction between amino acids and cross-link types and does not fully resolve the triple helical structure giving rise to collagen’s molecular stiffness. Additional earlier efforts to develop a coarse-grained (CG) model for the collagen triple helix were made by Gautieri, using the Martini 2 force field. Their approach parameterized bond length, bond angle, and torsion angle potentials by matching force-extension curves of small peptides, such as glycine-proline, glycine-proline-hydroxyproline, and glycine-proline-hydroxyproline-glycine, to AA-MD simulations (18). Despite these advances, extant CG models fail to capture collagen’s hierarchical structure in sufficient detail, especially its triple helical conformation and cross-links. The limitations of existing AA-resolution, mesoscopic, and earlier CG models hinder our understanding of how cross-linked collagen responds to mechanical perturbation at the microscale, highlighting the need for a new CG collagen model with improved resolution and accurate helical structure.

Here, we present a CG model for collagen based on the Martini 3 force field in conjunction with a Gō model, designed to perform large-scale CG-MD simulations of cross-linked collagen microfibrils under both equilibrium and high-force conditions (19, 20). We focused on understanding the molecular mechanisms underlying collagen rupture by simulating forces comparable to those that cause failure in biological systems. Our model successfully captures the force-extension behavior observed in AA simulations and enables CG-MD simulations of large collagen microfibrils under tension. The parametrization process combines standard Martini 3 fitting techniques with parameter optimization under force, making it suitable for nonequilibrium simulations. Combined with the automated generation of Martini 3 input files for MD simulations through our extended ColBuilder server, this model enables detailed investigation of the relationship between cross-link configuration, microfibrillar structure, and mechanical load across both microscopic and mesoscopic scales (21).

## Materials and methods

In Martini 3, each CG interaction site represents two to four heavy atoms plus their associated hydrogens, positioned at the center of geometry of the underlying AA structure. Martini 3 employs a building block principle for mapping a given AA structure to CG resolution and uses a mixed top-down and bottom-up parameterization strategy for the nonbonded and bonded interactions, respectively (19, 22). To model collagen microfibril’s complex structure—composed of multiple triple helices connected by cross-links—we first parameterized individual cross-links and triple helices before assembling them into microfibrillar structures. As the divalent hydroxylysino-5-keto-norleucine (HLKNL) and trivalent pyridinoline (PYD) cross-links are among the most prevalent and well-characterized cross-links in mature collagen tissue, they were chosen as a proof of concept. This approach can readily be extended to other cross-links to assess their role in collagen structure, dynamics, and mechanics.

To find the bead types and mapping schemes of the not yet parametrized subblocks, we considered symmetry arguments, polarity, and thermodynamic calculations. We evaluated three collagen systems of increasing complexity: first a single triple helix in water spanning one 67-nm-long D-band without intermolecular cross-links, second a fibrillar assembly of the same length comprising 40 triple helices with cross-links localized at two different regions, and third a fibrillar structure of the length of a full collagen molecule (335 nm) containing 267 triple helices with 10 cross-link regions (3, 23). An overview of the details of the simulated systems can be found in Table S1.

**Figure 1:**
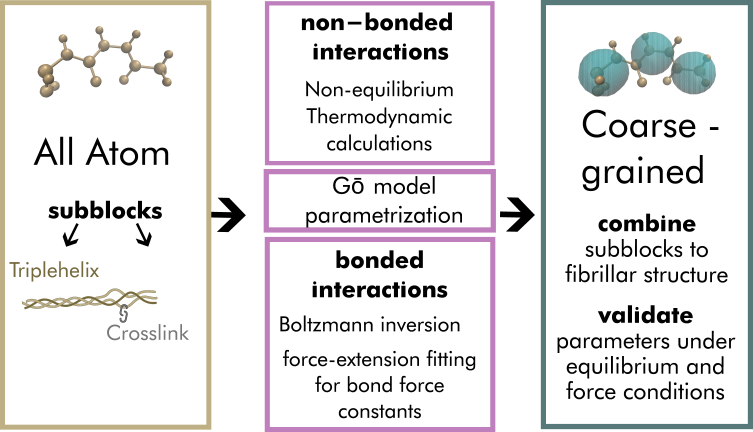
Overview of the parameterization process. The AA model is used to identify building blocks, which are independently parametrized by two main steps: the nonbonded interactions, the bead types, and the mapping schemes are found using thermodynamic calculations, symmetry, and polarity arguments. The bonded interactions are fitted via Boltzmann inversion to meet the AA data under equilibrium and under force conditions. If the building blocks are successfully determined, the two subsystems and their parameters are combined, and the structure is assembled via ColBuilder (21). To this end, the cross-linked microfibrillar structure is validated against experimental data and atomistic simulations under equilibrium and under force conditions taking into account different sized collagen systems.

The parameterization procedure for the CG model is outlined in Fig. 1. Specifically, we split the model into smaller building blocks and parametrized each block individually by determining the nonbonded interactions for both cross-links and the bonded interactions for the collagen triple helix. We aimed to reproduce the properties of the AA reference simulations and the experimental data with the Martini 3 simulations.

### All-atom simulation protocol

Our AA simulations were based on two different atomistic force fields: the Amber99SB-ildn^∗^ force field (AMBER) for simulating the collagen triple helix and 67-nm-long cross-linked fibril and the Chemistry at Harvard Macromolecular Mechanics (CHARMM36m) force field for parametrizing cross-links through nonequilibrium MD-based thermodynamic free energy calculations (24,25,26,27,28,29). The AMBER parameters for both cross-links were derived by Zapp through density functional theory, using the B3LYP functional for geometry optimization and Antechamber to determine partial charges (14, 30, 31). The topology of the collagen fibrils was built using an earlier version of ColBuilder, a pipeline to generate simulation-ready structures and topology files for collagen molecules, including cross-links (21). Our simulation protocol began with the solvation of each system in a rectangular simulation box with TIP3P water (26). After energy minimization, we neutralized the system with counter-ions replacing some of the water molecules. For the AA simulations, two different engines were used: smaller systems (67 nm) were simulated with GROMACS, whereas longer fibrils (335 nm)—AA systems of approximately 43 million atoms including water—were simulated on the Fugaku supercomputer using GENESIS (32, 33).

We used GROMACS for simulations of cross-links (v2023), triple helices (v2023), or the D-band of a fibril (v2020) (i.e., the small and intermediate AA systems) (34,35,36). The solvent was equilibrated around the protein by applying a harmonic potential to position-restrain the heavy atoms of the collagen backbone with a force constant of 1000 kJ mol^−1^ nm^−2^. Short-range electrostatic and van der Waals interactions were truncated at 1 nm using the Verlet scheme, and long-range electrostatics were treated using the particle-mesh Ewald method (37,38,39). The temperature was maintained at 300 K using the velocity rescaling thermostat with a time constant of *τ*_*T*_ = 0.1 ps, and the pressure was initially set to 1 bar. The Parrinello-Rahman barostat was used with *τ*_*P*_ = 2 ps and a compressibility of 4.5 × 10^−4^ bar^−1^ (40, 41). NPT and NVT equilibration steps were performed for 10 ns each. Periodic boundary conditions were applied throughout the simulations. For the GENESIS simulations (the large 335-nm AA system), we used a 1-nm cutoff for short-range electrostatic and van der Waals interactions. The system was equilibrated in the NVT ensemble at 300 K for 10 ns, followed by 20-ns NPT equilibration using the Martyna-Tobias-Klein barostat to maintain pressure at 1 atm (42). During the equilibration steps, position restraints were gradually decreased.

After obtaining a fully equilibrated system, assessed based on the convergence of the box volume, pressure, and temperature, we performed both equilibrium simulations and simulations under constant force pulling. For the latter, we defined each pulling group as the three caps of an individual triple helix, enabling force application at the center of each molecule. Forces were applied at both ends of collagen in the *z*-direction, aligned with the helical axis, to mimic physiological stress conditions. The magnitude of the applied force ranged from 300 pN to 1000 pN (per triple helix). To prevent unwinding of the triple helices, torque restraints were used for all AA systems. In the GROMACS simulations, a rotational force of 2000 kJ mol^−1^ nm^−2^ was applied to the caps of the AA models, following the approach of Zapp (14, 43). As a consequence of the applied force, the collagen triple helix/fibril straightened in the pulling direction until a new equilibrium state under force was reached.

#### Nonbonded interactions

For parametrization of the nonbonded interactions, we divided the collagen system into two blocks: cross-links and collagen triple helix. For the triple helix, we mapped the amino acids of the AA trajectory to CG resolution before finding the Martini 3 parameters. For the cross-links, we followed the Martini 3 parametrization procedure, which involves determining partition coefficients through nonequilibrium thermodynamic calculations (44).

##### Nonequilibrium thermodynamic calculations

The selection of Martini 3 bead types for the divalent HLKNL and trivalent PYD cross-links was based on a comparison of partition coefficients from Martini 3 with AA simulations (19). Although we acknowledge the limitations of this parametrization approach (45), experimental validation data for these specific cross-links are, to the best of our knowledge, currently not available. The partition coefficient measures the distribution of a compound between two immiscible phases, such as octanol and water, and is determined by the solute’s partitioning free energy. Using nonequilibrium MD-based free energy calculations, we estimated the free energy change to transfer each cross-link from water (*S*1) to a hydrophobic solvent (*S*2), in two thermodynamic steps, via

(1) $$\Delta \Delta G_{S1\to S2}=\Delta G_{\emptyset \to S2}-\Delta G_{\emptyset \to S1}\text{,}$$

with $\Delta G_{\emptyset \to S2}$ and $\Delta G_{\emptyset \to S1}$ corresponding to the free energy associated to transfer the cross-link to (forward transition) or from (backward) the hydrophobic solvent and to and from water to vacuum, respectively. We determined the solvation free energy in water, $\Delta G_{\emptyset \to S1}$, and in the hydrophobic solvent, $\Delta G_{\emptyset \to S2}$, using the maximum likelihood method to extract equilibrium free energies from nonequilibrium transitions, based on Crooks fluctuation theorem as proposed by Shirts (46, 47). Uncertainties in the solvation free energy differences, Δ*G*, were quantified through bootstrapping and error propagation. To generate the coordinate and topology files for both cross-links, we used the CHARMM-Gui input generator (29, 48, 49). We relied on the standard capping for the cross-link parametrization using GROMACS and the CHARMM36m force field. Specifically, the amide group of the cross-link’s peptide bond is capped with a single hydrogen (−NH_2_) and the carbonyl group with an alcohol group (−COOH).

Each compound was first solvated in water and anhydrous octanol, then neutralized, and finally thermodynamically integrated (50). For the trivalent cross-link PYD, we inserted a chloride ion to compensate for the charged nitrogen N^+^ on the pyridine ring, achieving a zero net charge system. To prepare for the nonequilibrium transitions, we energy minimized the system using the steepest descent algorithm and performed a NVT equilibration for 50 ns using the Berendsen thermostat at 300 K in each end state (51). After discarding the first 10 ns, we extracted 100 starting configurations from both end state ensembles.

Nonequilibrium alchemical transitions were completed using a coupling parameter *λ*, which was varied in both forward and reversed directions, i.e., 0→1 and 1→0, with *λ* = 1 corresponding to having the compound in solution and *λ* = 0 in vacuum. To sample the AA data, four separate replicate simulations, each consisting of 100 backward and 100 forward transitions of 2–10 ns in length, were carried out, yielding a cumulative simulation time of 4 *μ*s. The same cumulative time was reached by 100 forward and 100 backward transitions of the CG model. The GROMACS implementation for free energy calculations was used for this purpose (32). During nonequilibrium runs, the temperature was implicitly kept constant at 300 K using a stochastic dynamics integrator, whereas the pressure was maintained at 1 bar using the stochastic rescaling barostat with *τ*_*P*_ = 5 ns and a compressibility of 4.5 × 10^−5^ bar^−1^ (52, 53). A Gapsys soft-core potential was selected (*α*_*LJ*_ = 0.85, *σ*_*LJ*_ = 0.3, *α*_*Q*_ = 0.3) to prevent singularities when changing the Lennard-Jones and Coulomb interactions together (54). The work distributions associated with the forward and backward nonequilibrium transitions were finally collected to quantify the free energy of transferring each compound between solvents, using the *pmx* package developed by Gapsys et al. (54, 55). This procedure was performed while iterating over bead types and mapping schemes to match the free energy estimates from Martini 3 to AA reference simulations. Although the Martini 3 convention is to use wet octanol as a reference solvent (19), we initially employed dry octanol for parametrization to ensure consistency with our nonequilibrium AA workflow. To account for recent advances in QM calculations and the findings of Isik et al. (45), we also compared the logP values obtained by the QM-tool ORCA (56) with SMD solvent(57), machine learned predictions (58) (reported in the supporting material) and Martini 3 thermodynamical integration. The latter were obtained with equivalent sampling as our original free energy calculation while solvating with a “wet” octanol (concentration 92:8 octanol:water) to compare against QM(SMD) predictions (19, 45, 59, 60).

#### Bonded interactions

We followed the standard Martini 3 framework, i.e., using AA reference data to parameterize bond lengths, angles, and dihedrals. We performed direct Boltzmann inversion by fitting a Gaussian *P*(*q*) to the measured probability distributions to derive the Martini 3 force field parameters, treating each bonded term separately (61):

(2) $$P\left(q\right)=C_{q}\cdot \;\exp\;\left(-\frac{1}{2}\frac{{\left(q-\mu_{q}\right)}^{2}}{\sigma_{q}^{2}}\right)\text{,}$$

where *q* denotes a single degree of freedom (bond distance *r*_*ij*_, bond angle *θ*_*ijk*_, or torsion angle *ϕ*_*ijkl*_), and *μ*_*q*_, $\sigma_{q}^{2}$, and *C*_*q*_ are the mean, variance, and amplitude of the fitted Gaussian, respectively. For the Boltzmann inversion, we assumed each degree of freedom to be independent in the canonical ensemble, following a Boltzmann distribution (61):

(3) $$P\left(q\right)=Z^{-1}\cdot \;\exp\;\left(-\beta \cdot V^{CG}\left(q\right)\right)\text{,}$$

where *Z* is the partition function for the ensemble, and *V*^*CG*^(*q*) is the Martini 3 potential. By comparing the mean *μ*_*q*_ and variance $\sigma_{q}^{2}$ of the Gaussian function (Eq. 2) to the Boltzmann distribution (Eq. 3), we obtained the equilibrium bond value (*μ*_*q*_) and an initial force constant under equilibrium (yielding proper *σ*_*q*_) of each bonded term in the Martini 3 force field.

We performed inverse Boltzmann fitting separately for each of the two building blocks. For the triple helix parameters, we performed AA equilibrium simulations of three 67-nm-long collagen molecules in water. These three triple helices were obtained by splitting the 300-nm-long collagen molecule into five parts: two telopeptide regions (at each end) and three central 67-nm segments. The telopeptide regions were discarded, and the three centered segments were chosen as parametrization configurations. Each segment was run for 100 ns, before applying a center-of-geometry-based mapping scheme to the protein backbone atoms. We then extracted bond lengths, bond angles, and torsion angles between backbone beads (BBs), using their probability distributions as our major parametrization targets while considering their helical shape, particularly the experimental results for the triple helix (62,63,64,65). Additionally, we conducted fitting under force to fine-tune bond force constants. For both cross-links, we also determined the bonded terms with Boltzmann inversion and optimized the bond force constants during simulations under force.

##### Gō model

The standard Martini 3 force field does not inherently preserve the helical structure of the collagen molecule. To address this, we implemented a Gō model, following the work of Souza, which adds a Lennard-Jones potential applied to virtual sites that move with the protein BBs (20, 66). Specifically, we restricted the Gō-like potentials to each triple helix, that is, to intrahelical interactions, as shown in Fig. S9. Note that the influence of the Gō model’s potential well depth on the elongation under high forces is negligible. For this reason, we opted to rely on the default parameters, including a potential well depth of *ϵ*_*r*_ = 9.414 kJ mol^−1^ (67). To generate the topology of the collagen fibrils, we relied on ColBuilder (21). More details about the design of the Gō model and the generation of the fibrillar structure can be found in the supporting material in Topology generation.

### Coarse-grained simulation protocol

For the CG simulations, we followed the standard Martini 3 parameter setup for the neighbor list, electrostatic, and van der Waals interactions (68). Specifically, the neighbor list was defined with a cutoff at 1.35 nm, whereas the reaction-field algorithm cutoff the electrostatics at 1.1 nm with a relative permittivity of *ϵ*_*r*_ = 15 (69, 70). These parameters follow the Martini 3 simulation guidelines available at https://www.cgmartini.nl. All simulations were performed using GROMACS (v2023) (32).

For simulating a single collagen triple helix, we first solvated the structure in a rectangular simulation box using Martini 3 water beads. We then neutralized the system with counter-ions and performed energy minimization using the steepest descent algorithm. Temperature stabilization followed for 5 ns at 300 K using the velocity rescaling thermostat with *τ*_*T*_ = 1 ps. Pressure equilibration was achieved using the Berendsen barostat, maintaining 1 bar for 3 ns with a compressibility of 3 × 10^−5^ bar^−1^ and *τ*_*P*_ = 12 ps. In the production run, we set the pressure to 1 bar using isotropic pressure coupling with the Parrinello-Rahman barostat. For higher-order crystal structures, we followed similar energy minimization steps but implemented a longer multistep NVT and NPT equilibration procedure (see Fig. S10). For the 335-nm-long microfibril, we inserted additional water beads, in several insertion steps between NPT runs, to ensure adequate solvation. After achieving equilibrated CG collagen molecules and fibrils under isothermal-isobaric conditions, we performed pulling production runs. Although the setup for the CG system matched the AA conditions, for the CG model, we decided not to use torque restraints because the force constants we applied in the AA simulations were too low for the CG simulations, and we had no systematic basis for choosing appropriate force constants. Also, these restraints were unnecessary for the 335-nm-long CG collagen fibril due to its structural stability. The trajectory analysis and structural properties were computed using the protein helices analysis modules from MDAnalysis 2.5 (71, 72). For other properties, we used GROMACS tools (32). For equilibrated properties, we discarded the initial 20 ns, 30 ns, or 150 ns of trajectories, depending on the system size.

## Results

### Mapping of the cross-links and caps

**Figure 2:**
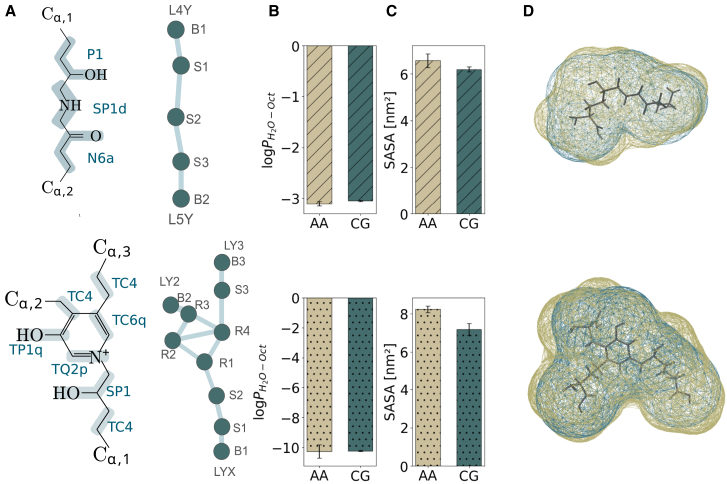
Mapping and bead type selection of the collagen cross-links with Martini 3. The divalent HLKNL (*top*) and the trivalent PYD (*bottom*) cross-link were considered. (*A*) Mapping and bead types are shown, highlighting the atoms grouped in each Martini 3 bead type (*left*) and the connectivity with an alpha-numeric numbering (*right*). (*B*) Octanol/water $\log\;P_{H_{2}O-Oct}$ values were obtained from AA and CG simulations to validate the proper thermodynamic partitioning of the cross-links. (*C*) Solvent-accessible surface area (SASA) and (*D*) Connolly surface area from AA simulations (*ocher*) and CG simulations (*dark turquoise*) confirmed the proper size of the CG cross-links.

Specifically, for the HLKNL cross-link, we developed a symmetrical mapping relative to the central secondary amine group (Fig. 2 *A*); a small *SP1d* bead represents the hydrogen donor characteristics of this amine group. Standard four-to-one mapping was applied to the 1-propanol and 1-propanone groups, using a polar *P1* bead for the alcohol and an intermediate hydrophilic *N6a* bead for the ketone group. The aromatic structure of the PYD cross-link required small and tiny beads (Fig. 2 *A*, *bottom*). The central pyridine ring was represented by four tiny beads: a *TQ2p* bead for the ring nitrogen, a *TP1q* bead for the phenol group, a *TC6q* bead for the benzene moiety, and a *TC4* bead at the ortho-bridging carbon that connects to the C_*α*_ of the lysine residue (*LY2*). This rhombic arrangement (*R1*, *R2*, *R3*, *R4* in Fig. 2 *A*, *bottom*), preserves the structural rigidity of the aromatic ring. The ring structure could only withstand such high forces with this rhombic structure of covalent bonds. Each of these four bonds was found to be crucial for maintaining ring stability under pulling forces, preventing the separation of the two arms of the trivalent cross-link attached to the same triple helix. The nontrivial bonds (*R2*–*R4* and *R3*–*R4*) cannot be straightforwardly replaced by constraints. Despite the planarity of the ring, we decided to not define a dihedral angle for this degree of freedom, since this led to high numerical instabilities in the simulations under force. Moreover, similar rhombic arrangements have been successfully used for mapping aromatic systems in CG models (44), validating our approach. For the beads attached to the ring, we used angles to maintain the system’s geometry. To account for the protonated pyridine nitrogen, we distributed partial charges across the tiny beads, assigning *q* = 0.7 e, *q* = 0.2 e, and *q* = 0.1 e to the *TQ2p*, *TP1q*, and *TC6q* bead, respectively, following the AMBER charge distribution. We applied q-labeled beads even for charges below 0.25 to emphasize the delocalization of charges. This choice is not in line with the Martini 3 guidelines, but this bead typing yielded good agreement with the partitioning behavior from AA simulations. For the C_*α*_ connections to the helices, we selected moderate polar *TC4* beads showing good agreement with the partitioning data (supporting material: Fig. S6). The link between the charged nitrogen and C_*α*,1_ was represented by a polar *SP1* bead.

We based our bead type selection by comparing partition coefficients for both cross-links from Martini 3 with AA simulations (70). The partition coefficient of each compound was determined in an octanol/water system using nonequilibrium MD-based free energy calculations. The transfer-free energies obtained through this method are $\Delta \Delta G_{W\to O}=-19.06\pm 0.07\;\text{kJ}\;{\text{mol}}^{-1}$, for the divalent cross-link, and $\Delta \Delta G_{W\to O}=-60.63\pm 0.24\;\text{kJ}\;{\text{mol}}^{-1}$, for the trivalent cross-link. From these transfer-free energies, the partition coefficients were obtained via Eq. 1. We observed good agreement between the partition coefficients for the divalent and trivalent cross-links from Martini 3, as visualized in Fig. 2 *B*, showing that the chosen bead types properly capture the thermodynamic partitioning of the cross-links. We further validated our bead type selection by comparing partition coefficients obtained by QM(SMD)/machine-learned prediction methods for both cross-links (70). The logP values are summarized in Table 1 (computational details for comparative methods are provided in the supporting material). For the neutral HLKNL cross-link, AA-dry and CG-dry closely agree, showing that the chosen bead types properly capture the thermodynamic partitioning of the cross-links. When wet octanol is used instead, CG-wet and QM(SMD) converge within uncertainty, indicating that our CG mapping is robust across solvent definitions. For the charged PYD cross-link, AA-dry and CG-dry yield artificially high values, reflecting the ill-defined nature of logP for charged species. By contrast, CG-wet and QM(SMD) agree well with empirical predictions. This is consistent with observations from the SAMPL7 blind challenge, where charged molecules showed large method-dependent deviations (73).

**Table 1:** Comparison of logP values across different methods

Cross-link	AA-*dry*	CG-*dry*	CG-*wet*	QM(SMD)
HLKNL	−3.11 ± 0.05	−3.05 ± 0.01	−3.50 ± 0.69	−2.9
PYD	−10.26 ± 0.44	−10.22 ± 0.04	−5.46 ± 0.76	−4.3

All partition coefficients were obtained via Eq. 1 by first estimating the free energy. The “AA-dry” and “CG-dry” partition coefficients were used for parametrization, whereas the “CG-wet” and QM(SMD) columns were obtained for further validation. The CG values agree well with their respective reference (AA or QM).

These comparisons demonstrate that our parametrization strategy yields transferable bead assignments: although AA-dry was used for initial parametrization, the close agreement between CG-wet and QM(SMD) values validates our approach across different solvent definitions. Given that QM(SMD) approaches are increasingly considered state-of-the-art for logP predictions (73), the agreement of the corresponding values confirms our bead choices. We chose to fit the model to the partitioning data as the prior target, trying also to be as consistent as possible with the Martini 3 guidelines. However, in some cases, trade-offs arose where simultaneously fulfilling both requirements was not possible. We consider the presented mappings to be a reasonable compromise while acknowledging that alternative bead choices may also reproduce the data well and could be more suitable for other applications.

### Parametrization of bonded terms for cross-links

We followed the previously mentioned AA simulation protocol under equilibrium conditions to obtain an equilibrated ensemble for each cross-link in water. We computed bond distances, bond angles, and torsion angles between cross-link beads from the mapped trajectory and analyzed their probability distributions. We then adjusted the CG probability densities to match the mapped AA distributions, following the standard bottom-up procedure for parametrizing new molecules with Martini 3 (19, 74). To reproduce the atomistic structure, the lengths of bonds between Martini 3 beads were rescaled based on solvent-accessible surface area (SASA) comparisons with AA simulations. The adjustments are typically small (on the order of 0.1–0.2 nm) but essential for ensuring that the CG model reproduces the correct molecular volumes and packing behavior (details in Fig. S7). As expected, the SASA value for the divalent cross-link from Martini 3 simulations (6.2 ± 1.1 nm^2^) agrees with the estimates from AA simulations (6.5 ± 0.3 nm^2^). Due to the challenging geometry of the trivalent cross-link, we accepted larger deviations for this cross-link, with a value of 8.24 ± 0.2 nm^2^ for Martini 3 and 7.04 ± 0.3 nm^2^ for AA simulations (Fig. 2 *C*). Further details on the Martini 3 force field parameters for both cross-links can be found in Tables S4–S6.

To examine structural differences at the molecular level, we analyzed Connolly surfaces derived from AA and CG simulations (Fig. 2 *D*). Although the agreement is not perfect, the CG model reasonably reproduced the HLKNL cross-link Connolly surface from the AA structure. Similar agreement was observed for the Connolly surface of the trivalent PYD cross-link. The differences in size likely arise from the inherent loss of fine-grained atomic details upon coarse-graining. These differences become negligible in the larger fibril structure, where bonded interactions dominate under applied force.

### Parametrization of bonded terms for triple helical backbone

**Figure 3:**
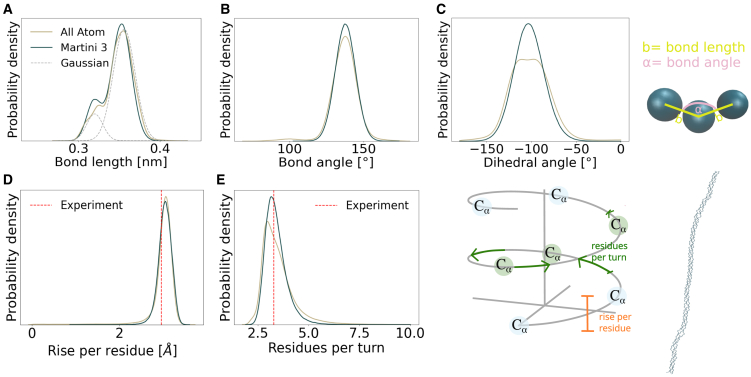
Parametrization of bonded interactions and shape of collagen triple helix under equilibrium conditions. Mapped AA trajectory (simulated in equilibrium, *ocher*) versus CG trajectory (*dark turquoise*). (*A*) Bond length including the two Gaussian fits (*gray*), (*B*) bond angles, (*C*) dihedral angles, (*D*) rise per residue, and (*E*) residues per turn (*right*). All figures show qualitative agreement of the two different setups. Additional properties (local bends and twist of the helix) can be found in the supporting material (Fig. S2).

The collagen molecule exhibits a triple helical structure that confers unique mechanical properties through both intra- and interhelical interactions. These interactions are critical for understanding the molecule’s behavior under physiological conditions and its role in tissue mechanics. Therefore, the parametrization of bonded terms is of particular importance. For the backbone structure, we first assigned bead types based on the Martini 3 parameters for amino acids. We combined conditional probability distributions with Boltzmann inversion to reproduce the triple helix symmetry and shape. Bond length analysis revealed a bimodal distribution (Fig. 3 *A*), indicating that backbone bead distances are sequence specific rather than uniform. We fitted this complexity using a bimodal Gaussian distribution—comprising two Gaussian functions—which allowed us to estimate the mean and variance of each distribution separately, thereby accounting for the nonequidistance of the mapped collagen backbone.

Based on our analysis, the first Gaussian, representing the lower peak of the bimodal distribution, was centered at *μ*_*b*,1_ = 0.318 nm, with *σ*_*b*,1_ = 0.010 nm, whereas the second was located at *μ*_*b*,2_ = 0.354 nm, with *σ*_*b*,2_ = 0.013 nm. Sequence analysis identified hydroxyproline (HYP) and proline (PRO) as primary contributors to the first peak, consistent with their helix-stabilizing role. We therefore implemented two bond potentials: one for general amino acid pairs and another for HYP/PRO-X combinations (where X is any amino acid). To decide which bond distance to assign to each amino acid type, we first determined the intersection point of the two Gaussian distributions at *x*_0_ = 0.329 nm, based on the overlapping probability densities. We computed backbone bead distances and applied this threshold as a filter. Next, we assigned probabilities to each residue type based on whether its backbone bead was at the start (BB_1_) or end (BB_2_) of the bond. The detailed probability assignment process and validation are available in Fig. S2. To achieve better agreement with the AA simulation peaks, we optimized the model’s accuracy by marginally adjusting the bond lengths to *μ*_*b*,1_ = 0.320 nm and *μ*_*b*,2_ = 0.356 nm. This dual-potential approach was essential to capture the sequence-specific structural variations in the collagen backbone. We applied equivalent procedures for bond angle and dihedral angle parametrization, both of which exhibited unimodal probability distributions (Fig. 3 *B* and *C*). The parameters derived through our systematic optimization approach are presented in Table 2.

**Table 2:** Martini 3 force field parameters

Bonded terms	Equilibrium value [nm or °]	Force constant [kJ mol^−1^ nm^−2^ or kJ mol^−1^]
BB_1_−BB_2_^a^	0.356	18,000
BB_1_−BB_2_^b^	0.320	34,000
BB_1_−BB_2_−BB_3_	138	152
BB_1_−BB_2_−BB_3_−BB_4_	76	17

Force constants were derived from simulations under constant force to capture the mechanical response accordingly. Of note, the bond length value is chosen to be marginally larger than the Boltzmann inversion suggested since the peaks of the Gaussian distributions from Martini 3 tend to be at smaller values than the AA data.

a Bond length potential for all amino acid sequences, except those starting with a proline-like type of amino acid.

b Bond length potential for amino acid sequences, such as HYP/PRO-X.

We validated the parameters by comparing two key observables: rise per residue (characterizing axial extension) and residues per turn (describing radial geometry) (Fig. 3 *D* and *E*) (62, 63, 65, 75, 76). The rise per residue was slightly higher than the experimental value of 0.290 nm measured by x-ray fiber diffraction (0.307 nm for AA, and 0.304 nm for CG simulations) (62, 63). Both simulations underestimated residues per turn compared with experiments. The AA simulations show a peak at 2.96 residues per turn (10% below experimental values), whereas CG simulations exhibit a peak at 3.15 residues per turn (5% below experimental measurements with 3.28 residues per turn) (64, 65, 75). These comparisons demonstrate that our CG collagen model successfully captures both the axial and radial degrees of freedom of each helical strand and largely reproduces the shape of the collagen triple helix.

**Figure 4:**
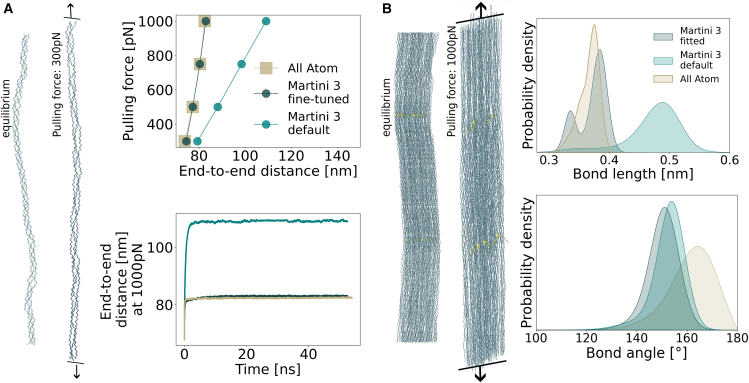
Fitting and validation of the obtained backbone-bonded parameters in simulations under force. (*A*) Triple helix under force: visualization of a triple helix in equilibrium and under force conditions (*left*). Converged values of the end-to-end distance of the triple helix simulated under forces from 300 pN to 1000 pN are shown for the adjusted bonded parameters, “Martini 3 fine-tuned” (backbone force constants of 18,000 kJ mol^−1^ nm^−2^/34,000 kJ mol^−1^ nm^−2^), and for default Martini 3 (force constant of 4000 kJ mol^−1^ nm^−2^) and AA simulations for comparison (*right, top*). A representative timeseries at 1000 pN applied pulling force (*right, bottom*). (*B*) A 67-nm-long, trivalently cross-linked fibril under force. Structure under equilibrium and force conditions, where the yellow dots represent the cross-links (*left*). Bond length and angle width distributions of the fibrillar structure (*right*).

Equilibrium parameters did not accurately capture mechanical properties under applied force. Bond force constants were identified as the primary determinant of extension behavior of a triple helix, and thus also of fibrillar collagen structures, under force. As a result, we fine-tuned the force constant of the bond length potentials under force to capture the appropriate force response. Therefore, we performed simulations of a single triple helix under a range of pulling forces (300–1000 pN) using both AA and CG models (Fig. 4 *A*). We systematically explored the interplay between the Gō model and bonded parameters by testing different potential well depths under varying mechanical loads (see supporting material Fig. S9). Although the Gō model well depth significantly influences the molecular response below 300 pN (entropic force regime), this effect disappears above 1 nN. Rather, at such high forces, bond stretching dominates the mechanical response. This confirms that optimizing bonded parameters, rather than the Gō model epsilon, is the appropriate approach for reproducing collagen mechanics under high stress. Increasing bond stiffness successfully matched the AA response. For this, we increased the bond force constants to 18,000 kJ mol^−1^ nm^−2^ and 34,000 kJ mol^−1^ nm^−2^. For comparison, we also evaluated the standard Martini 3 force field with its default bond force parameter of 4000 kJ mol^−1^ nm^−2^, which showed significantly less accurate reproduction of the mechanical response.

We tested our model by evaluating the mechanical response of a 67-nm-long collagen fibril, cross-linked with both divalent (Fig. S14) and trivalent bonds (Fig. 4 *B*). To analyze backbone-bonded parameters under force, we mapped AA trajectories to CG resolution and compared distributions at the Martini bead level. Specifically, we examined the distributions of bond lengths and bond angles between BBs subjected to a force of 1000 pN applied at each triple helix. Under force, the bimodal bond length distribution observed in AA equilibrium simulations (Fig. 3 *A*) transforms into a single broad distribution, whereas the Martini 3 model maintains a distinct double-peak character. Despite this difference in distribution shape, stiffening the bonds in our model significantly improved the overall mechanical response of the fibril when compared with the AA data.

For the trivalently cross-linked fibril, the bond angles under force revealed partial deviations between the CG and AA simulations (Fig. 4 *B*). The fine-tuned CG model exhibits more restricted angular variations compared with the mapped AA trajectory, where the backbone angles show greater flexibility. Although this discrepancy could be partially attributed to the loss of information upon mapping from atomistic to CG resolution, we deliberately maintained higher angle force constants to preserve the characteristic triple helical structure, prioritizing biological relevance over exact flexibility matching. The trade-off between exact reproduction of AA flexibility and maintenance of helical stability reflects our focus on capturing the fundamental structural properties of collagen in the CG model. Future work could explore more sophisticated parameter balancing approaches.

### Force-extension dynamics

Finally, to evaluate the applicability of our model for large-scale simulations, we considered a 335-nm-long microfibril of collagen. Such systems consisted of ∼43 million atoms (at AA resolution) and 6.3 million beads (at CG resolution), highlighting the relevance of developing CG models for studying these types of systems. We examined the force-extension dynamics of this fibril under applied force, applying a pulling force of 1000 pN. For this case, we chose the trivalent PYD cross-link, because its challenging parametrization made it an especially rigorous test case for model validation.

**Figure 5:**
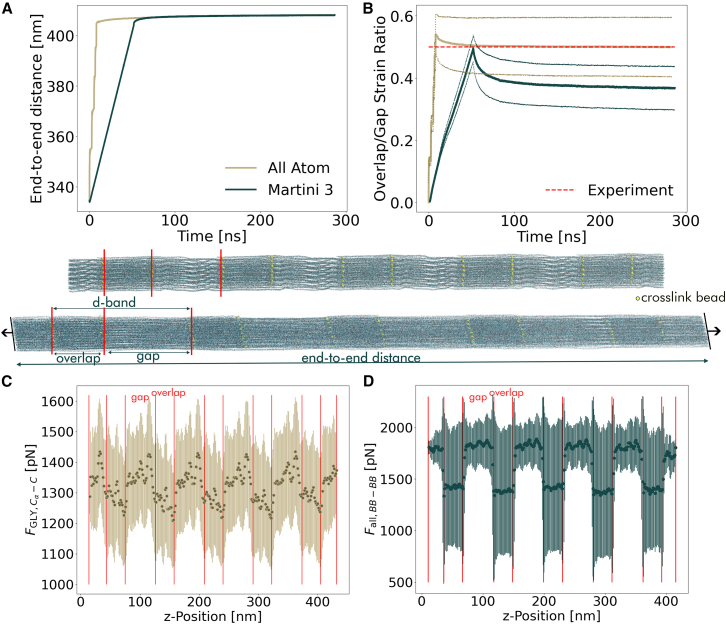
Force-extension dynamics of the 335-nm-long microfibril of collagen. (*A*) Timeseries of the end-to-end distance obtained from AA and Martini 3 simulations. (*B*) Overlap-gap strain ratio time series with the corresponding uncertainty: since the cross-link positions vary, we show the propagated error of the uncertainty of the overlap and gap length. (*C* and *D*) Detailed analysis along the *z*-axis of the fibril: binned mean of 300 bins evenly distributed along the *z*-axis and shows their *z*-position. For comparison, we show the forces between the *C*_*α*_-*C* atoms in glycine in the AA model and the forces within all backbone beads (BBs) in the CG model.

The end-to-end distance under 1000 pN force demonstrates that both AA and CG models converge to the same elongation, showing a similar force response (Fig. 5 *A*). To examine the structural deformation in detail, we calculated the average overlap/gap strain ratio $\left\langle \epsilon_{overlap,t}\right\rangle /\left\langle \epsilon_{gap,t}\right\rangle$ according to

(4) $$\frac{\left\langle \epsilon_{overlap,t}\right\rangle }{\left\langle \epsilon_{gap,t}\right\rangle }=\frac{\left\langle l_{overlap,t}-l_{overlap,0}\right\rangle }{\left\langle l_{gap,t}-l_{gap,0}\right\rangle }\text{,}$$

where *l*_*overlap*,*t*_ and *l*_*gap*,*t*_ are the length of the overlap and gap region at time *t*, respectively. The dotted lines in Fig. 5 *B* represent the propagated uncertainty of the gap and overlap measurements, arising from variations in cross-link positions within the fibril. We computed gap and overlap elongations as averages ⟨…⟩ across all complete segments (four and five, respectively) in the fibril. The AA data match the experimental value of 0.5 very closely. The CG data converge to a value around 0.4, indicating a greater stiffness of the overlap region relative to the gap region. Thus, our Martini 3 model overestimates this difference and finds the overlap region to be slightly stiffer compared with AA. We attribute this difference to the loss in structural details at the cross-link region, which slightly changes cross-link tilting and extension under force. This deviation highlights the critical role of cross-links as structural connectors in determining mechanical properties. Nevertheless, the agreement between our CG model and the AA data at this large scale (∼300 nm) is excellent.

To further characterize the mechanical response, we analyzed the force distribution along the fibril’s *z*-axis. For the AA model (Fig. 5 *C*), we analyzed C_*α*_-*C* bond forces in glycine residues ($F_{GLY,C_{\alpha }-C}$) as representative backbone markers. For the Martini 3 model, we examined the forces $F_{all,BB_{i}-BB_{i+1}}$ between all BBs (Fig. 5 *D*).

Although our force distribution analysis provides valuable insights into the mechanical response along the fibril axis, we acknowledge that this approach has limitations in capturing the full complexity of interhelical interactions. Specifically, hydrophobic interactions between collagen chains within the fibril may not be fully represented by this force-based analysis method. At the high pulling forces used in our simulations (1000 pN), mechanical response is dominated by covalent interactions (primarily cross-link mechanics and backbone bond stretching) rather than weaker noncovalent interactions such as hydrophobic effects. Additionally, the inherent smoothing of the free energy landscape in CG models reduces friction between triple helices, potentially leading to enhanced elongation compared with AA systems (77). This effect, combined with differences in load distribution between individual chains during mechanical deformation, represents a fundamental challenge in CG modeling that requires careful analysis. In the case of collagen, we expect that the interhelical water layer both at AA and CG levels reduces friction due to relative helix sliding. A complete characterization of these interaction effects between the triple helices is an important area for future model development.

The CG model showed a repeating force pattern across all regions, with consistently lower average forces in overlap regions compared with the gap regions. Furthermore, CG simulations showed wider force distributions in overlap regions, whereas the AA model exhibited uniform uncertainty across both regions. Notably, the AA data reveal higher forces at gap-to-overlap transitions, a feature not captured in the Martini 3 model. This discrepancy likely results from a combination of the reduced precision of the mapping procedure and the intrinsically smoothed nature of the CG force field. Despite these differences, recapitulation of the repeating force pattern enabled the CG model to reproduce the correct force elongations.

## Discussion

We have developed and validated a CG model for collagen using the Martini 3 force field. Our model enables the detailed investigation of cross-link interactions with triple helical structures, significantly improving upon previous approaches (17, 18). We have integrated our model into the framework of ColBuilder, enabling large-scale CG-MD simulations of collagen fibrils and a comprehensive analysis of how cross-link density and types influence collagen dynamics (21).

The unique structural characteristics of collagen presented distinct challenges for parametrization. Specifically, we encountered situations, when parameterizing the cross-links, where we had to balance adhering to the Martini 3 guidelines with reproducing the AA data, a trade-off that should be carefully considered in future development and application of the presented model. Also, the triple helical coil and GLY-X-Y pattern, where X is typically a PRO and Y a HYP, required refitting approaches. Despite these challenges, our model successfully reproduced key collagen-specific observables. To maintain the helical secondary structure, we implemented a Gō model. We observed that small systems, particularly single triple-helix ends, can exhibit partial unwinding under very high force. This unwinding behavior was not observed in biologically sized fibrillar systems (of several hundreds of nanometers like the one studied here), suggesting that system size and cross-linking provide natural structure stabilization. Although the current Martini 3 implementation cannot fully maintain the helical shape through applied potentials alone, that is, without the Gō model or similar ways of explicit secondary structure stabilization, future versions may offer new approaches for parametrizing helical proteins.

A key methodological contribution of our work addresses the challenge of out-of-equilibrium, stretched coarse-graining with Martini 3, for which no established protocols currently exist. Our observation that Martini 3 bonds require stiffening led us to prioritize bond potential fitting over angle potentials. We propose that bond forces are crucial for accurate force/pulling simulations, an insight that may be valuable for parameterizing other CG scales, such as the CALVADOS model (78), under force. We demonstrated feasibility of simulating exceptionally large systems (43 million atoms) on the Fugaku supercomputer at the Riken center. On standard high-performance computing clusters, we observed substantial computational efficiency with approximately 16-fold speed-up comparing the AA (0.31 ns/day/cpu) to the Martini 3 setup (5.0 ns/day/cpu). This enhanced performance enables extensions to the model, including system sizes that overcome boundary effects (Fig. S12) and enable better comparison with biological experiments and investigation of the effect of advanced glycation end products in collagen fibrils.

The Martini 3 force field’s coarse-graining approach offers several advantages beyond its computational efficiency. Although maintaining residue-level resolution, it provides more detailed structural representation than coarser models such as colbreaker (79) or the model from Depalle (16). Furthermore, our model’s compatibility with backmapping techniques creates opportunities for predicting reactivity with chemical detail, e.g., through reactive-MD pipelines like KIMMDY (14, 80). This capability will allow detailed analysis of rupture events and force distribution patterns in systems of 335-nm length and beyond.

## Data and code availability

The force field parameters are available via the ColBuilder GitHub repository (github.com/graeter-group/colbuilder). Simulation data produced can be found at https://doi.org/10.17617/3.MF8BBT.

## Acknowledgments

This work was supported by the 10.13039/501100007316Klaus Tschira Foundation, the 10.13039/501100004189Max Planck Society, and the 10.13039/501100000781European Research Council (ERC) (grant number 101002812) and by a Research Grant from HFSP (ref.-no: RGP025/2024) and the award DOI: https://doi.org/10.52044/HFSP.RGP0252024.pc.gr.194178. F. Gräter acknowledges funding from the 10.13039/501100001659Deutsche Forschungsgemeinschaft (DFG, German Research Foundation) under Germany’s Excellence Strategy for the Excellence Cluster “3D Matter Made to Order” (EXC-2082/1–390761711). F. Grünewald acknowledges funding from the 10.13039/501100007316Klaus Tschira Stiftung gGmbH (Independent PostDoc). D.M. acknowledges funding from the Marie Skłodowska-Curie Actions Individual Fellowship (grant number 101151862). The authors acknowledge support by the state of Baden-Württemberg through bwHPC for computational resources on the bwForCluster Helix and the 10.13039/501100001659German Research Foundation (DFG) through grant EXC-2082/1-390761711. Y.S and J.J acknowledge the 10.13039/501100001700MEXT program for promoting research on the supercomputer Fugaku (JPMXP1020200101) and 10.13039/501100001700MEXT program for Big-data-driven bio/synthetic polymer science to create absolutely circular materials (JPMXP1122714694) and Data-Driven Research Methods Development and Materials Innovation Led by Computational Materials Science (JPMXP1020230327).

## Author contributions

M.B. and J.B. parametrized the model with Martini 3 and performed the simulations as well as the analysis. F. Grünewald and C.A.-S. helped with understanding, using, and tuning the Martini 3 force field to the intended model. M.B. and D.M. built ColBuilder, which was used to generate the simulated system. J.J. and Y.S. implemented and performed the all-atom molecular dynamics simulations on Fugaku. J.B., D.M, M.B., and F. Gräter wrote the manuscript with input from all authors. F. Gräter conceived and supervised the project.

## Declaration of interests

No competing interest is declared.

## Supporting material

Document S1. Figures S1–S18 and Tables S1–S6

Document S2. Article plus supporting material
