# Supplementary material for: Coarse-grained Martini 3 model for collagen fibrils
Source: Biophys J. 2025 Oct 10;125(2):581–93. doi: 10.1016/j.bpj.2025.10.012 (PMC12969027; doi:10.1016/j.bpj.2025.10.012)
Supplement: Document S1. Figures S1–S18 and Tables S1–S6 [file mmc1.pdf]

Biophysical Journal, Volume *125*

Supplemental information

Coarse-grained Martini 3 model for collagen fibrils

Matthias Brosz, Johanna Buck, Fabian Grünewald, Debora Monego, Jaewoon Jung, Yuji Sugita, Camilo Aponte-Santamaría, and Frauke Gräter

Supplemental Information: Coarse-Grained Martini 3 Model for Collagen Fibrils

Table S1: **Overview of the simulated collagen systems**: the single triple helix, the divalent and trivalent crosslinks, the 67 nm- and 335 nm-long fibril. We show the structure length in nm, the number of triple helices (3 alpha chains), number of crosslinks, number of D-bands, number of particles (excluding water molecules), and the simulation time of a single replicate.

Property	Triple helix	Free energy simulations (Crosslinks)	PYD	HLKNL	Short fibril	Long fibril
Length [nm]	67	-	-	-	67	335
Triple helix	1	-	-	-	41	225
Crosslinks	-	1	1	1	16	248
D-band	1	-	-	-	1	5
Particles AA	8571	-	60	44	73 k	4.9 Mio
Particles CG	1940	-	11	5	323 k	1.1 Mio
Simulation time AA [ns]	100 /segment	4×(100× forward/ backward), 2–10 ns each	100	100	100	450
Simulation time CG [ns]	100 /segment	1x(100× forward/ backward), 2–10 ns each	100	100	100	300

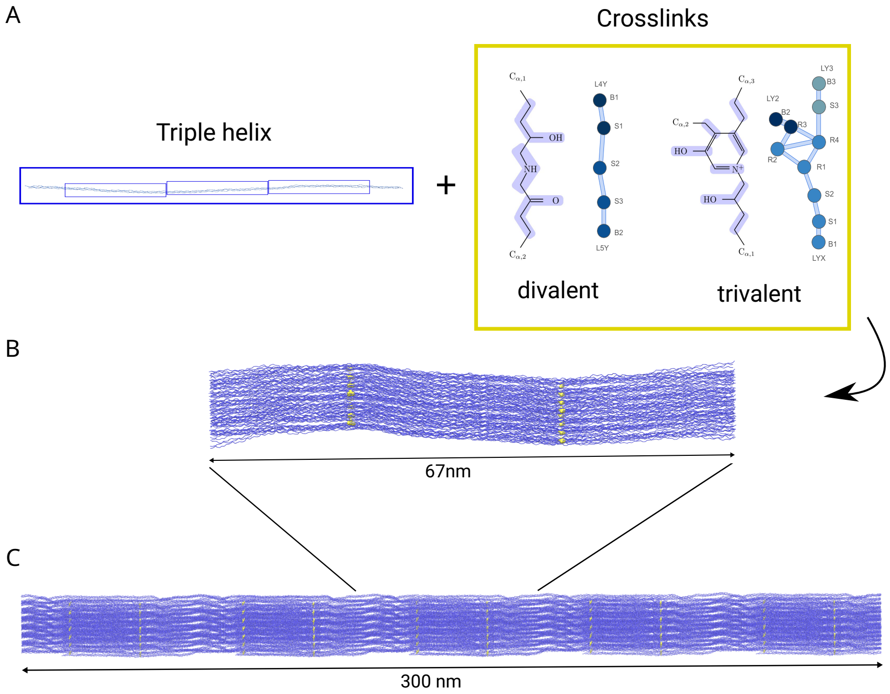

Figure S1: **Overview of collagen systems simulated for parametrization.** **A** Schematic showing triple helix building blocks (*left*) combined with divalent and trivalent crosslinks (*right, yellow box*). **B** 67 nm-long fibril containing 40 triple helices with crosslinks at 2 regions (*yellow dots*). **C** 335 nm-long fibril containing 267 triple helices with crosslinks at 10 regions (*yellow dots*). The three independently simulated 67 nm-long triple helix segments without crosslinks (highlighted rectangles in panel **A**) were used for backbone parametrization, while the fibrillar systems (**B** and **C**) were used to parametrize and validate crosslink interactions and mechanical properties.

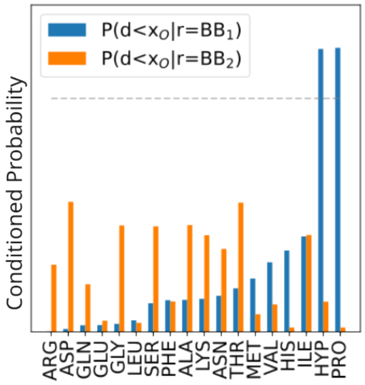

Figure S2: **Residue-specific bond length distributions in triple helix parametrization.** Conditional probability of specific amino acids at the start of bonds (BB_1, given that the bond length (d) is shorter than the intersection point (x_0) of the bimodal gaussian distribution, from equilibrium distributions. This analysis focuses on the first peak of the bimodal distribution ($d < x_0$), revealing that proline and hydroxyproline have very high probability of being involved in shorter bonds. Based on these results, bonds involving proline-type amino acids were assigned optimized bond lengths and force constants.

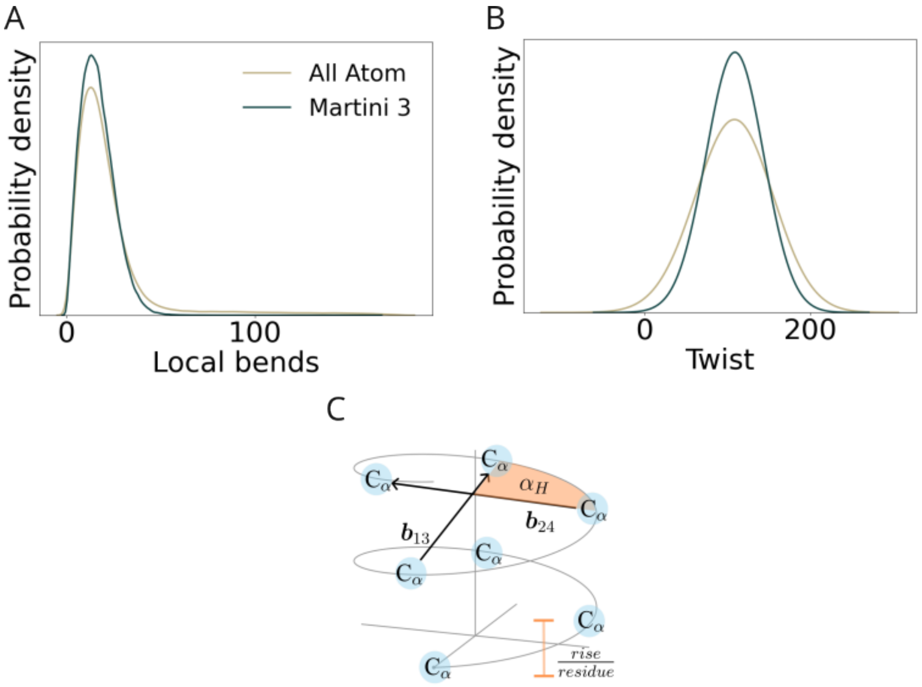

Figure S3: **Triple helical shape parameters.** **A** Local bending angles between helical axes separated by three residues, comparing AA (*brown*) and CG (*gray*) simulations. **B** Helical twist angle distributions showing good agreement between AA and CG models. **C** Schematic illustration of helical geometry calculations. For twist calculations, four adjacent C_α-atoms are selected and connected in sequence by Euclidean vectors to determine bisectors b_{13} and b_{24} (*black arrows*). The helical twist α_H is calculated as the dot product of these bisectors to estimate the enclosed angle. Local bending describes the deviation of local helical segments from perfect linearity. These geometric parameters validate that the CG model accurately reproduces the characteristic triple helical shape observed in AA reference simulations, confirming proper structural representation at the coarse-grained level.

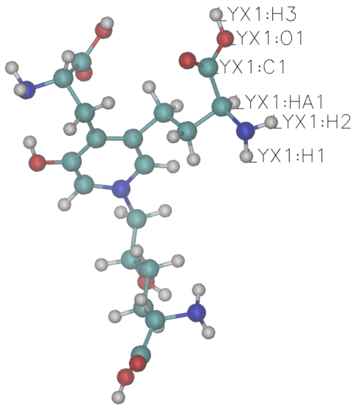

Figure S4: **Capping strategy for crosslink parametrization: Atomistic structure of the PYD crosslink.** The trivalent PYD crosslink connects two chains from one triple helix to one chain from another triple helix, requiring capping of three terminal ends for parametrization. To avoid using AMBER force field ACE/NME caps that would alter partitioning behavior, CHARMM36m force field was selected for free energy calculations since it provides suitable standard capping without additional modifications. Using the *pdb2gmx* command with CHARMM36m, each peptide bond terminus was capped: the amide group with a single hydrogen (-NH_2) and the carboxylate group with another (-COOH). This capping strategy preserves the crosslink's native chemical environment while enabling accurate thermodynamic property calculations for CG parametrization. The labeled atoms (LYX1:H3, LYX1:O1, etc.) indicate the CHARMM36m naming convention used in the calculations.

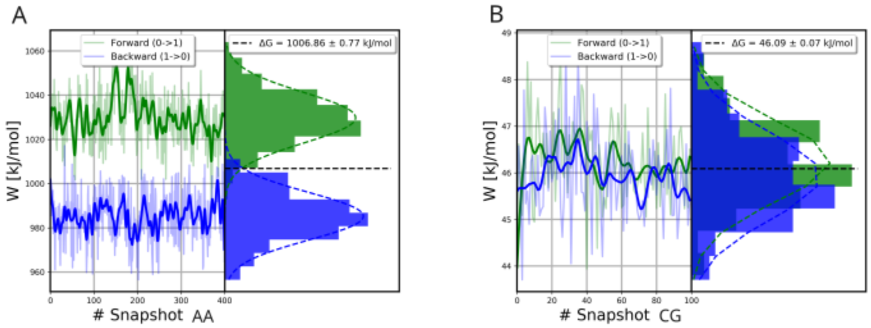

Figure S5: **Work distributions from non-equilibrium MD-based free energy calculations for crosslink parametrization.** **A** All-atom simulations of the divalent crosslink showing 400 forward ($0 \to 1$, *green*) and 400 backward ($1 \to 0$, *blue*) transitions between water and octanol phases. **B** Corresponding CG-MD simulations with 100 forward and 100 backward transitions. The work distributions (*left*) and cumulative work plots (*right*) demonstrate the sampling quality for free energy estimation using Crooks fluctuation theorem. The calculated free energy differences (ΔG values shown) validate the thermodynamic consistency between AA and CG models. This non-equilibrium approach enabled iterative optimization of bead types and mapping schemes to match the partitioning behavior of crosslinks between our CG model and AA reference simulations, ensuring accurate representation of crosslink hydrophobic/hydrophilic character in the CG model.

Free energy estimates and logP value comparison

QM(SMD) calculations

For the QM calculation we used the QM-software Orca 6.0 [9, 8] with the B3LYP DFT functional with the DEF2-SVP basis set, we performed a geometry optimization and computed vibrational frequencies numerically (finite differences of energies) using Grimme's D4 dispersion correction. These settings were also suggested in the manual of ORCA 6.0 [9, 10]. For the solvent, we chose to use the inexpensive solvent type SMD [5] with the specification *water* or *1-octanol*.

Thermodynamic integration for the CG *wet* calculations

To obtain a logP value in a solvated octanol configuration, we chose to perform DTI - thermodynamic integration using the same sampling as for the method presented for AA and CG *dry* methods described in detail in the main text. We applied the relation of 920:80 (octanol:water) [2] as solvent for *wet* octanol representing the experimental conditions which were used to fit the ML data. We first solvated the box with octanol+water or water, followed by an energy minimization, nvt and npt equilibration and the thermodynamic integration. We followed Martini3 standard methods found in [6]. We modified this approach using lambda in steps of 0.05 and a longer simulation time of 50 ns for each lambda value. After the integration, the free energy of solvation was computed

via the GROMACS bar module which uses Bennett acceptance ratio (BAR).

Using AlogP with SMILES

For calculating AlogP, we used the virtual computational chemistry tool which is available online using a SMILES as an input. Further details can be found in the respective publications of the tool [12].

Using RDKit with SMILES

We also applied the machine learned predicting tool RDKit on the SMILES of both crosslinks and obtained further values for comparison [4].

Crosslink	CG *wet*	QM(SMD)	AlogP	RDKit
HLKNL	-3.50 ± 0.69	-2.9	-4.2	-2.1
PYD	-5.46 ± 0.76	-4.3	-4.2	-5.5

Table S2: Comparison of logP values across different predicting methods. The AlogP values are of the same magnitude which does not correspond to our expectations. Due to the charge of the PYD molecule we expected a higher logP value than for HLKNL. The RDKit value, however, shows agreement with the trend and also the value for the PYD crosslink agrees (when including the Cl in the SMILES).

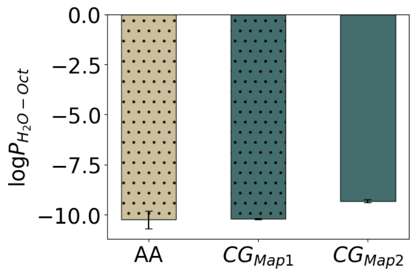

Label	Map 1 (chosen)	Map 2 (alternative)
B1	SP2	SP2
S1	TC3	TC4
S2	SP1	SP1
R1	TQ3p	TQ2p
R2	TP1q	TP1q
R4	TC5q	TC6q
R3	TC5	TC4
S3	TC3	TC4
B2	SP2	SP2
B3	SP2	SP2

Figure S6: **Bead type optimization through octanol/water partitioning validation.** Comparison of partition coefficients ($logP_{H_2O} \rightarrow Oct$) for the trivalent crosslinks using different Martini 3 bead types at specific backbone connection sites. Initially, $TC3$ beads were selected following standard Martini 3 protocols for positions $S1$, $S3$ of the trivalent crosslink and for $R3$ a $TC5$ bead (see Fig. 2A). However, comparison with AA reference simulations showed that $TC4$ beads provide better agreement at those positions (e.g., AA: 10.26, CG ($Map1$): 10.22 and CG ($Map2$): 9.33). This iterative optimization process of trying out many different alternative mappings where we show one example here ensured that the CG model accurately reproduces the thermodynamic properties of crosslinks in different solvent environments, validating the final bead type selection for the crosslink parametrization.

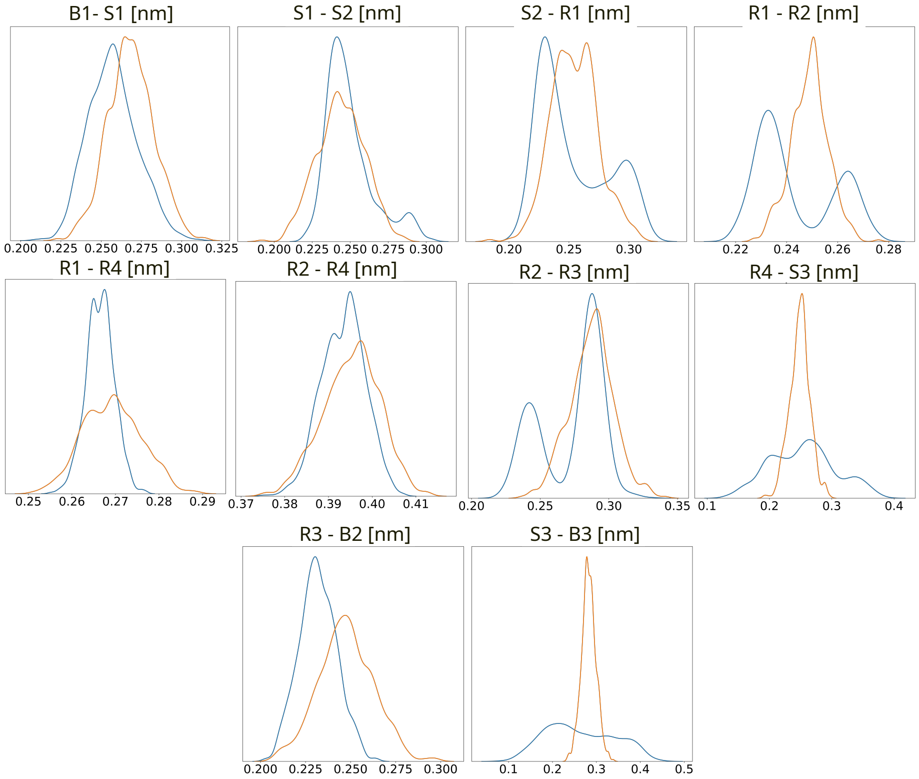

Figure S7: **Bond length distributions showing SASA-based optimization validation.** Probability densities for all bonded terms in the trivalent crosslink comparing center-of-geometry mapped AA trajectories (*blue*) with SASA-optimized Martini 3 simulations (*orange*). Each panel shows a different bond pair within the crosslink structure (*B1-S1*, *S1-S2*, *S2-R1*, etc, referring to the bead labels in Fig. 2A). The SASA-based optimization procedure adjusted bond lengths to better reproduce the structural characteristics observed in AA simulations, accounting for the different effective sizes of atoms versus CG beads. Without SASA-based adjustments, the CG distributions would have identical means to the initial Boltzmann inversion by construction, but would not accurately capture the true structural flexibility. The improved agreement between AA and CG distributions after SASA optimization demonstrates that this approach successfully maintains that crosslink's structural integrity while preserving its dynamic behavior in the CG representation.

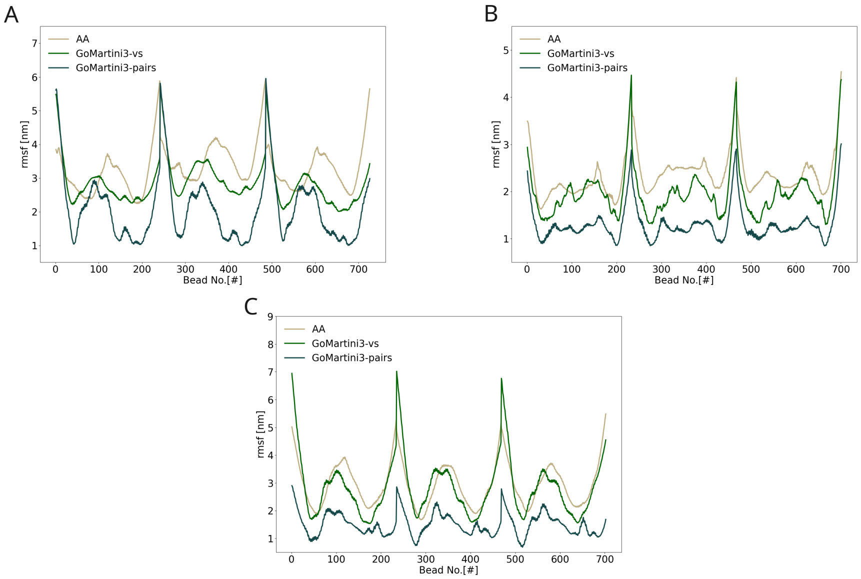

Figure S8: **Gō model implementation comparison: Root-mean-square-fluctuations for virtual sites versus pairwise interactions.** RMSF profiles comparing AA reference (*brown*), standard GōMartini 3 with virtual sites (*green*), and our modified GōMartini 3 with pairwise interactions (*blue*) across three different collagen molecules segments (**A, B, C**). The standard "vs" approach uses virtual sites for Gō model interactions following default GōMartini 3 protocols, while our "pairs" approach defines interactions directly between bead pairs. Although both CG approaches reproduce the overall magnitude of AA fluctuations, the pairwise method shows reduced noise and improved stability compared to virtual sites. Importantly, the pairwise approach dramatically reduces memory requirements, making it computationally feasible for large fibrillar systems where the virtual site implementation becomes impractical due to excessive memory demands. The three panels represent different regions along the collagen molecule, demonstrating consistent performance across the entire structure.

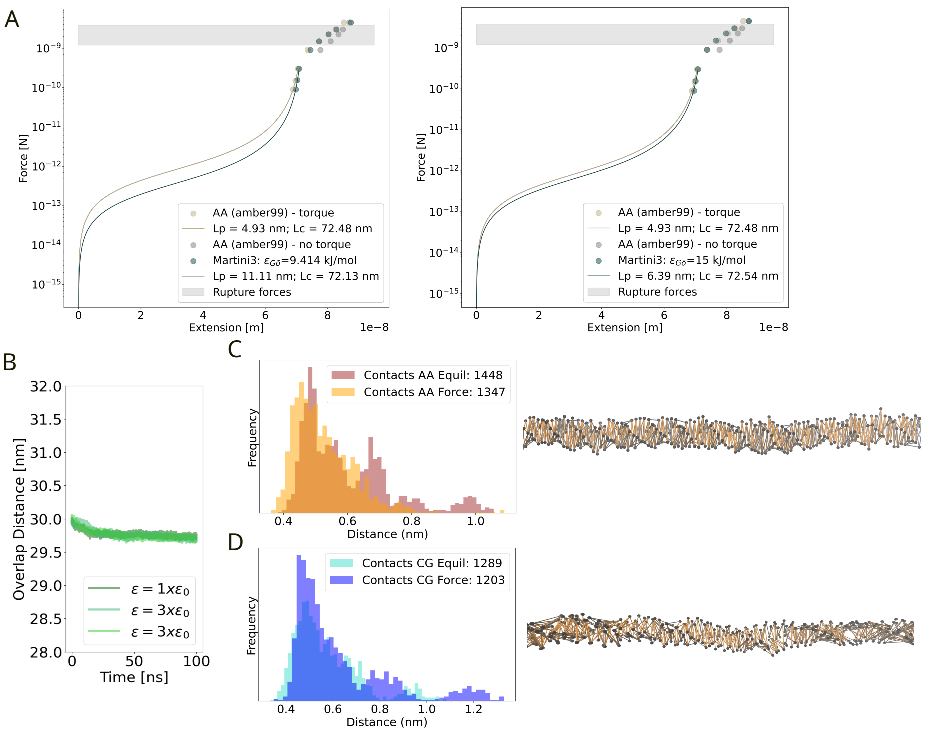

Figure S9: **Gō model details: Influence of the potential well depth ϵ** **A** End-to-end distance of the triple helix for different forces observed from AA and CG simulations. Also, the influence of the torque restrains, applied to both ends of the collagen triple helix, for the AA simulations is shown. **B** Overlap length of a trivalently crosslinked fibril with varying ϵ values (1x, 3x, 5x the original value of $\epsilon_{\text{G}\bar{\text{o}}} = 9.414\,\text{kJ}\,\text{mol}^{-1}$). **C** Gō model contacts under equilibrium and under force shown as a distribution of distances and vizualised in an example triple helix under force. The number of contacts shown is measured with a threshold of 1.1 nm which corresponds to the Lennard-Jones cutoff. Orange contacts persist under force while gray contacts are below the tresshold.

In our force-extension analysis, we observe two different regimes for this semiflexible polymer under tension: Entropic regime at low forces: The Gō model epsilon significantly affects the force-extension behavior, with the default value ($\epsilon_{\text{G}\bar{\text{o}}} = 9.414\,\text{kJ}\,\text{mol}^{-1}$) providing good agreement with AA simulations. Enthalpic regime at high force, $> 300\,\text{pN}$: Under high pulling-forces, collagen's mechanical response to stress is driven by stretching stiffness as the chain becomes stretched and larger than its persistence length. In this regime, the Gō model has minimal influence because it is not designed to handle such high forces. Both Gō model parameter sets converge to similar behavior, and additional tests with scaled epsilon values (factors of 3, 5, shown in the right panel of the figure) confirm that changes to the Gō model parameter have negligible impact on the high-force stretching response. As we see through stretching also the contacts below the Lennard-Jones cutoff of 1.1 nm is reduced. As expected, the distances of Gō contacts also shift to higher values.

Topology generation of crosslinked Martini 3 collagen fibrils

To generate the topology of the collagen fibril, we used a bottom-up approach by first constructing individual collagen molecule topologies, and second merging them based on crosslinking information. For each single collagen molecule, we pre-processed the coordinate file and used the *martinize2* command from the *vermouth* package to generate the Martini 3 force field topology and coordinate file [3]. We then applied the *contact map* analysis script to identify close contacts in the atomistic structure, considering both geometric and chemical criteria, namely interatomic distances and amino acid types. Using the Martini 3 coordinate file and the atomistic *contact map* as inputs for the *create_goVirt.py* script from Poma, we generated the GōMartini 3 topology and coordinates files by combining physics-based and structure-based modeling approaches[11]. This pipeline was applied to each triple helix to account for their intrahelical interactions. To combine crosslinked triple helices, we used the *connect* file from ColBuilder, which enables generation of fibrillar structures[7]. Next, crosslinks were incorporated at sites selected based on experimental knowledge of lysine/hydroxylysine positions as defined in ColBuilder. We then combined the topology files of crosslinked collagen molecules and added the bonded potentials between crosslink beads. We modified the standard GōMartini 3 approach by defining Gō-like Lennard-Jones interactions between virtual sites as *pairs*, rather than assigning new bead types for each virtual site. Using a cut-off of 1.1 nm, this modification enabled efficient generation of multi-million particle systems using *grompp* from GROMACS (v2023)[1]. The original definition of virtual site interactions in GōMartini 3 is impractical with *grompp* due to excessive memory and time requirements: We tested the time required to compile different sized test system (table below) to extract at which size the pairs approach is needed. Of note, this virtual site memory issue persists even with newer versions of GROMACS when simulating large systems. For fibrillar structure which were the aim in this work, we were not able to compile with gmx grompp within sensible timescales using current standard RAM. The final topology combined the GōMartini 3 files for pairs and triplets of collagen triple helices with the bonded terms for divalent and trivalent crosslinks.

We tested a compliation of an energy minimization at the start of the simulation without any solvent beads. The limit was measured on a standard Laptop with 31GB RAM GROMACS version 2022.5. The same result was also obtained with a interactive node of 32GB memory and GROMACS version 2024.3. Importantly, the Gō model needs to be of high density because of collagens given density and the triplehelical structure which we want to preserve. Table S3 shows why the decision was necessary to switch to pairs in order to simulate at scale.

System (no water)	No. of virtual sites	Virtual sites, t	Pairs, t
Triple helix	702	16 s	9 s
30 nm long fibril, dc = 18	14k	$> 7h$	29 s
40 nm long fibril, dc = 18	18k	fails	31 s
67 nm long fibril	26k	fails	37 s
300 nm long fibril, dc =40	393k	fails	231 s

Table S3: Overview of systems and the compilation time of the Gō model approaches virtual sites and pairs.

Equilibration procedure for Martini 3 collagen fibrils

Dependent on the collagen system, we performed a multi-step equilibration procedure to enable production run simulations. The 67 nm-long crosslinked collagen fibril was equilibrated in the NVT ensemble for 5 ns and for 30 ns with the NPT ensemble using a $2 - 5$ fs timestep.

For the 335 nm collagen fibril, we applied a multi-step equilibration procedure as given in Fig. S10. Moreover, we observed water holes subsequent to our pulling simulations. Thus, we introduced a resolvation step before we continued with further pulling, where we inserted more water molecules and performed shorter NPT-equilibration steps in between. We then continued with the pulling simulations where we first performed first constant velocity pulling in order to let the fibril adapt to the force, followed by the production run under constant force. Every pull group consisted of the three caps at the end of a single triple helix. We pulled with 3 nN per triple helix, thus 1 nN per strand. In the case of the 335 nm-long collagen fibril, this accumulates to 606 nN at each side.

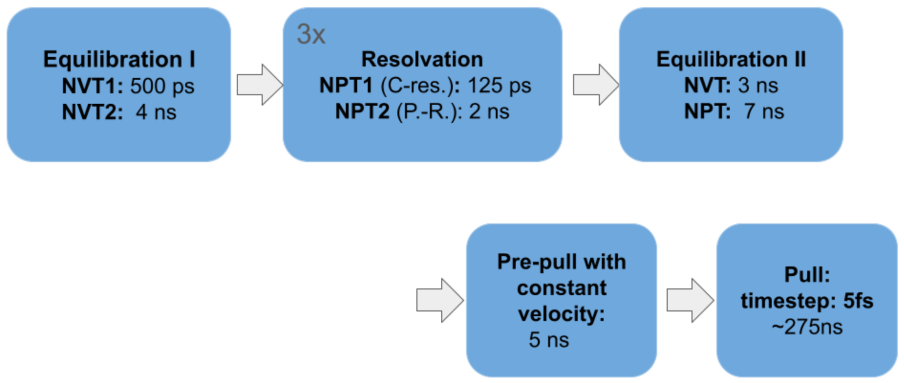

Figure S10: **Equilibration procedure for the 335 nm-long fibril.** Flowchart showing the five-step equilibration protocol used for large fibrillar systems, with timescales and ensemble conditions for each stage.

Martini force field parameters

Table S4: Martini 3 force field bonded terms of the protein backbone beads

**Bond/Angle**	**Value**	**Force Constants**
BB(all amino acids except proline/hydroxyproline)- BB	0.356 nm	18000 kJ mol^{-1} nm^{-2}
BB(proline/hydroxyproline)- BB	0.320 nm	34000 kJ mol^{-1} nm^{-2}
epsilon of Gō potential	–	9.414 kJ mol^{-1}
BB_1-BB_2-BB_3	138°	152 kJ mol^{-1}
BB_1-BB_2-BB_3-BB_4	76°	17 kJ mol^{-1}

Table S5: Martini 3 force field bonded terms of the trivalent PYD crosslink.

**Bond/Angle**	**Value**	**Force Constants**
B1-S1	0.270 nm	18000 kJ mol^{-1} nm^{-2}
S1-S2	0.250 nm	15000 kJ mol^{-1} nm^{-2}
S2-R1	0.260 nm	14000 kJ mol^{-1} nm^{-2}
R1-R2	0.250 nm	72000 kJ mol^{-1} nm^{-2}
R1-R4	0.270 nm	72000 kJ mol^{-1} nm^{-2}
R2-R4	0.395 nm	68000 kJ mol^{-1} nm^{-2}
R3-R4	0.370 nm	12000 kJ mol^{-1} nm^{-2}
R2-R3	0.230 nm	12000 kJ mol^{-1} nm^{-2}
R4-S3	0.290 nm	11000 kJ mol^{-1} nm^{-2}
R3-B2	0.250 nm	18000 kJ mol^{-1} nm^{-2}
S3-B3	0.250 nm	18000 kJ mol^{-1} nm^{-2}
B1-S1-S2	180°	150 kJ mol^{-1}
S1-S2-R1	180°	150 kJ mol^{-1}
S2-R1-R2	180°	150 kJ mol^{-1}
S2-R1-R4	100°	200 kJ mol^{-1}
R2-R3-B2	140°	150 kJ mol^{-1}
R4-S3-B3	140°	150 kJ mol^{-1}

Table S6: Martini 3 force field bonded terms of the divalent crosslink.

**Bond/Angle**	**Value**	**Force Constants**
B1-S1	0.310 nm	10000 kJ mol^{-1} nm^{-2}
S1-S2	0.415 nm	7000 kJ mol^{-1} nm^{-2}
S2-S3	0.365 nm	5000 kJ mol^{-1} nm^{-2}
S3-B2	0.360 nm	9000 kJ mol^{-1} nm^{-2}
B2-S3-S2	100°	150 kJ mol^{-1}
S1-S2-S3	140°	150 kJ mol^{-1}
B1-S1-S2	140°	150 kJ mol^{-1}

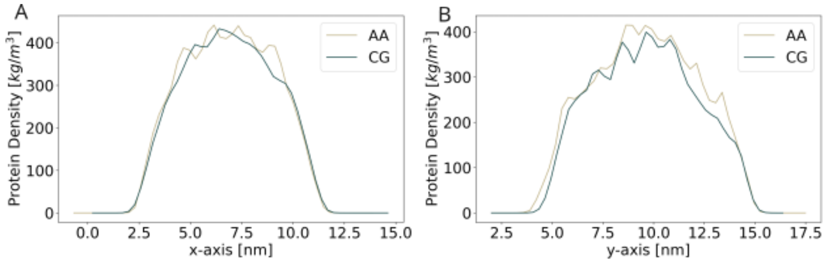

Figure S11: **Lateral spacing in the fibrillar model.** Comparison of protein density along the x- and y-axes from AA CG simulations of a 67 nm stretched model, averaged over the final 20 ns. Water is excluded. The close agreement in lateral density profiles indicates similar diameters and lateral packing under applied force.

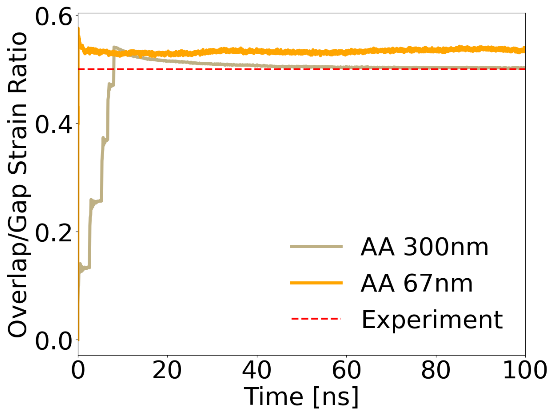

Figure S12: **Boundary effects in fibrillar models of different lengths.** Overlap/gap strain ratio comparing 67 nm vs. 335 nm all-atom fibril models under applied force, with experimental reference value (*red dashed line.* The smaller 67 nm fibril shows significantly higher strain ratios due to boundary effects, where the limited system size constraints natural deformation patterns. The longer 335 nm fibril exhibits strain ratios closer to the experimental value, demonstrating that boundary effects are minimized in larger systems. This comparison shows the need for sufficiently large fibrillar models to accurately capture physiological mechanical behavior and avoid artifacts from finite size effects.

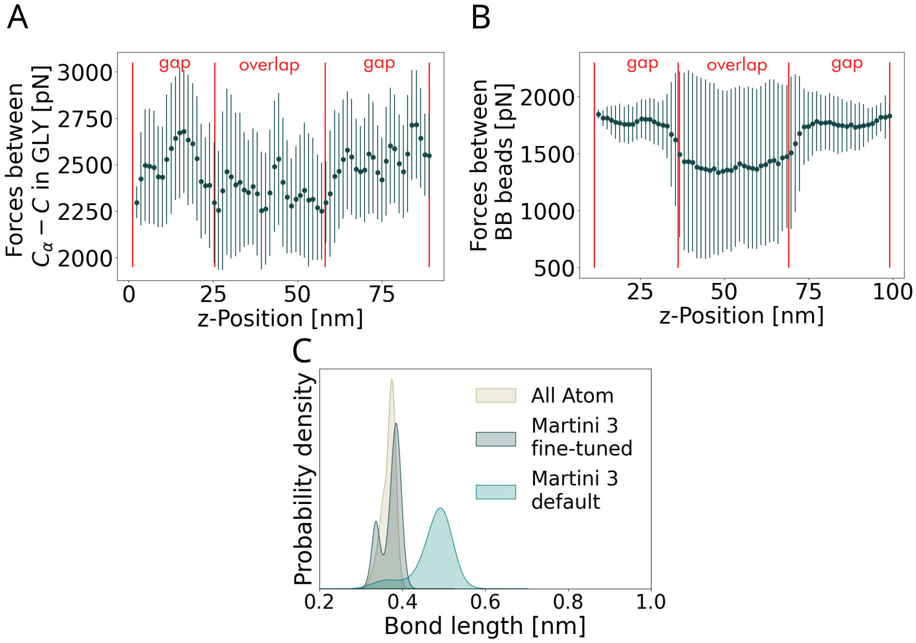

Figure S14: **Force distribution analysis of divalent crosslinked 67 nm fibril under force. A)** C_α-C bond forces in glycine residues from all-atom simulations, projected along the fibril axis and averaged over the final 70 ns. **B)** Backbone bead forces from Martini 3 simulations under applied force, showing similar periodicity. Both panels demonstrate the characteristic gap/overlap pattern with red vertical lines marking structural transitions. **C** Bond length distributions comparing all-atom reference with fine-tuned and default Martini 3 parameters, validating the improved structural representation. The force profiles reveal that divalent crosslinks produce mechanical behavior very similar to trivalent crosslinks (see Figure 4B), with lower forces in overlap regions and higher forces in gap regions. This analysis confirms that the coarse-grained model accurately reproduces the heterogeneous stress distribution along the fibril length observed in all-atom simulations.

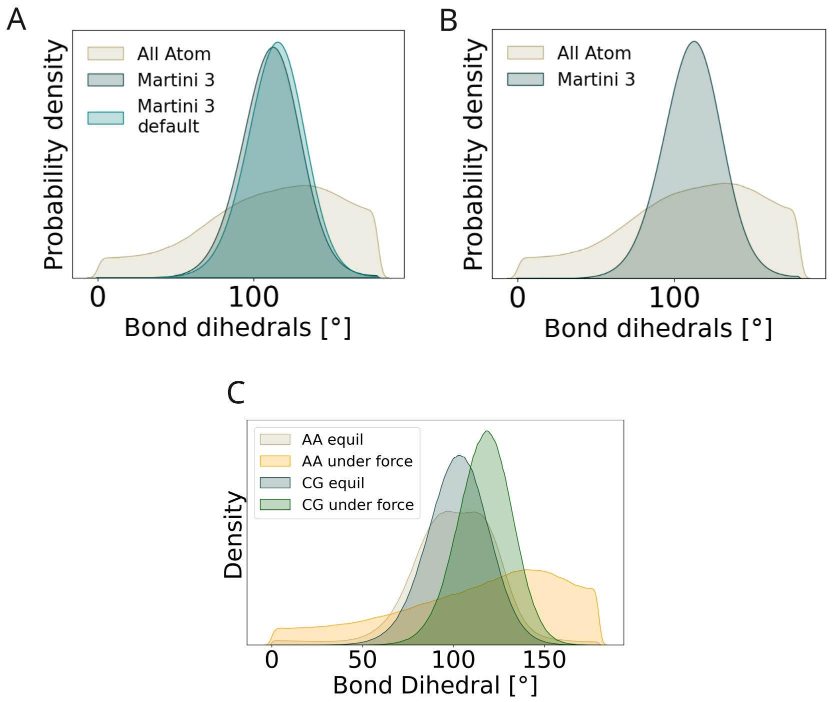

Figure S13: **Dihedral angle distributions comparing equilibrium and force conditions.** Backbone bead dihedral angle for **A)** 67 nm fibril with trivalent crosslinks, **B)** 67 nm fibril with divalent crosslinks, and **C)** 67 nm triple helix under equilibrium versus applied force. The AA data was mapped to CG resolution. Under force, both AA and CG models show rightward peak shifts, indicating structural deformation. However, AA simulations exhibit much broader angular distributions under force, covering nearly all possible angles, while CG models maintain more restricted Gaussian distributions. This difference reflects the fundamental limitation of CG dihedral parametrization: dihedrals are fitted under equilibrium conditions and cannot fully capture the extreme flexibility observed in AA simulations under force. However, as demonstrated in the main results, this does not compromise the model's ability to accurately reproduce key mechanical properties such as force-extension behavior and fibril elongation.

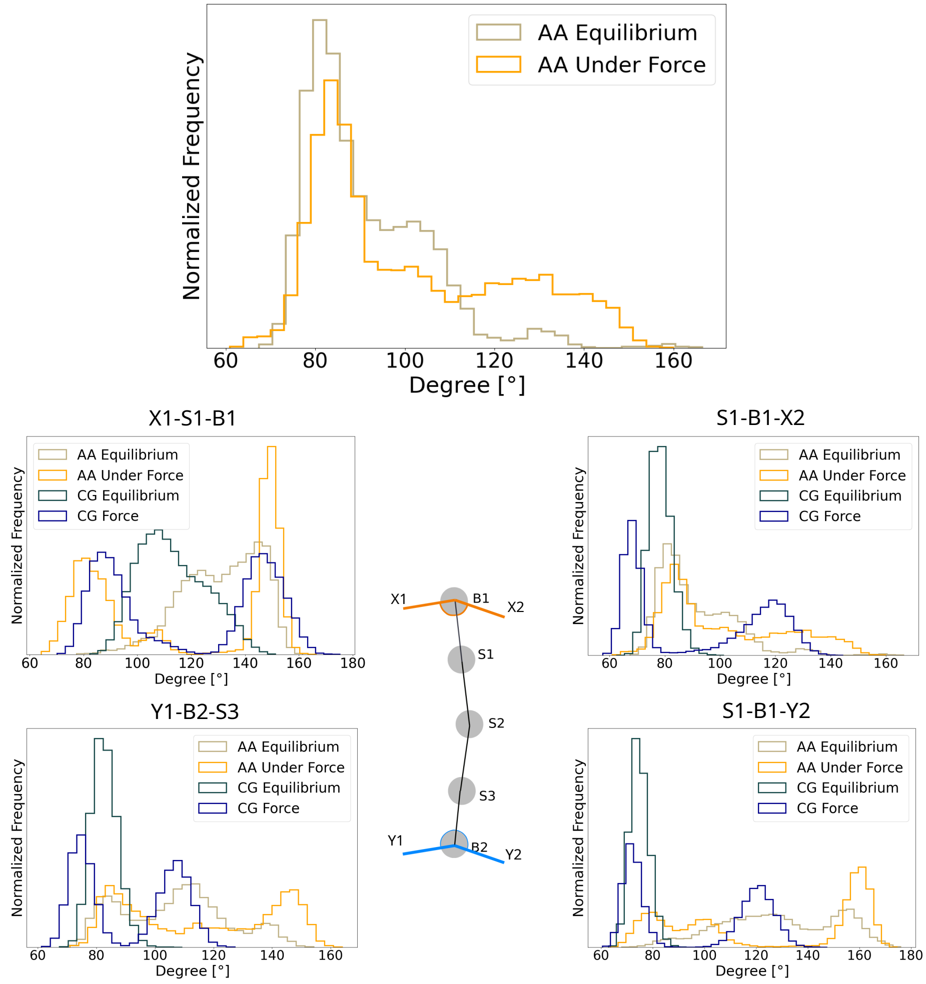

Figure S15: **Crosslink-protein backbone bond angle distributions from the 67 nm-long divalently crosslinked collagen fibril.** Overall bond angle distributions from AA simulations showing wide-spread angles under equilibrium conditions (*gray, left*) and under 1 nN pulling force (*orange, left*). Due to the wide distribution which is shown in the AA distribution, we decided to avoid tighter constraints in these regions due to the risk of overfitting to uncertain or non-representative geometries. Comparison of four specific crosslink-backbone angles (*X1-B1-S1*, *S1-B1-X2*, *Y1-B2-S3*, *S3-B2-Y2*) between AA and CG simulations under equilibrium and force conditions (*right*). The bond angle labels correspond to the crosslink mapping scheme (see Fig. 2A). Good agreement between AA and CG distributions validates the crosslink parametrization, with both models showing similar angle distributions under equilibrium and the expected narrowing of angular flexibility under applied force.

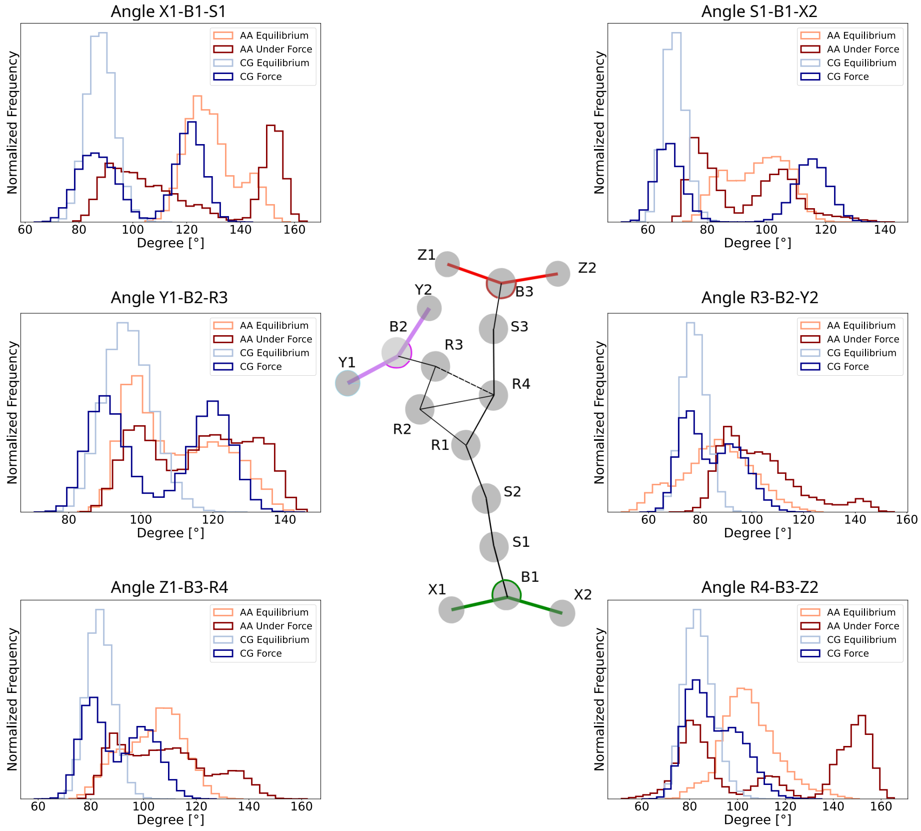

Figure S16: **Crosslink-protein backbone bond angle distributions from the 67 nm-long trivalently crosslinked collagen fibril.** Schematic of the trivalent crosslink structure showing the six bond angles analyzed (*X1-B1-S1*, *S1-B1-X2*, *Y1-B2-R3*, *R3-B2-Y2*, *Z1-B3-R4*, *R4-B3-Z2*) (*center*). Comparison of the AA and CG bond angle distributions between the protein backbone and the trivalent crosslink under equilibrium and force conditions. Overall, the CG data captures the dynamics of the bond angles reasonably well, with good agreement between AA and CG models for most angles.

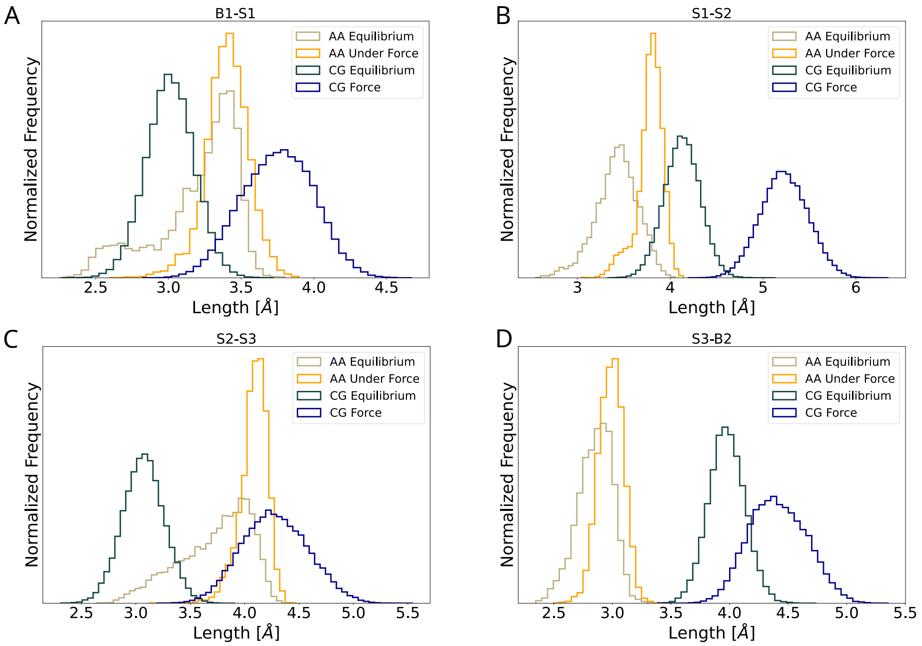

Figure S17: **Crosslink bond length distributions from the 67 nm divalently crosslinked collagen fibril. A-D** Divalent crosslink bond length distributions for specific bond pairs (*B1-S1*, *S2-S3*, *S3-B2*) comparing equilibrium simulations with simulations under applied force (1000 pN). Bond labels correspond to the divalent crosslink mapping scheme (see Fig. 2A). We observe similar trends between CG and AA reference simulations under both equilibrium and force conditions, with both models showing characteristic bond extension under applied force. However, slight deviations in peak positions and distribution widths are visible, reflecting the coarse-grained approximation while maintaining overall structural consistency.

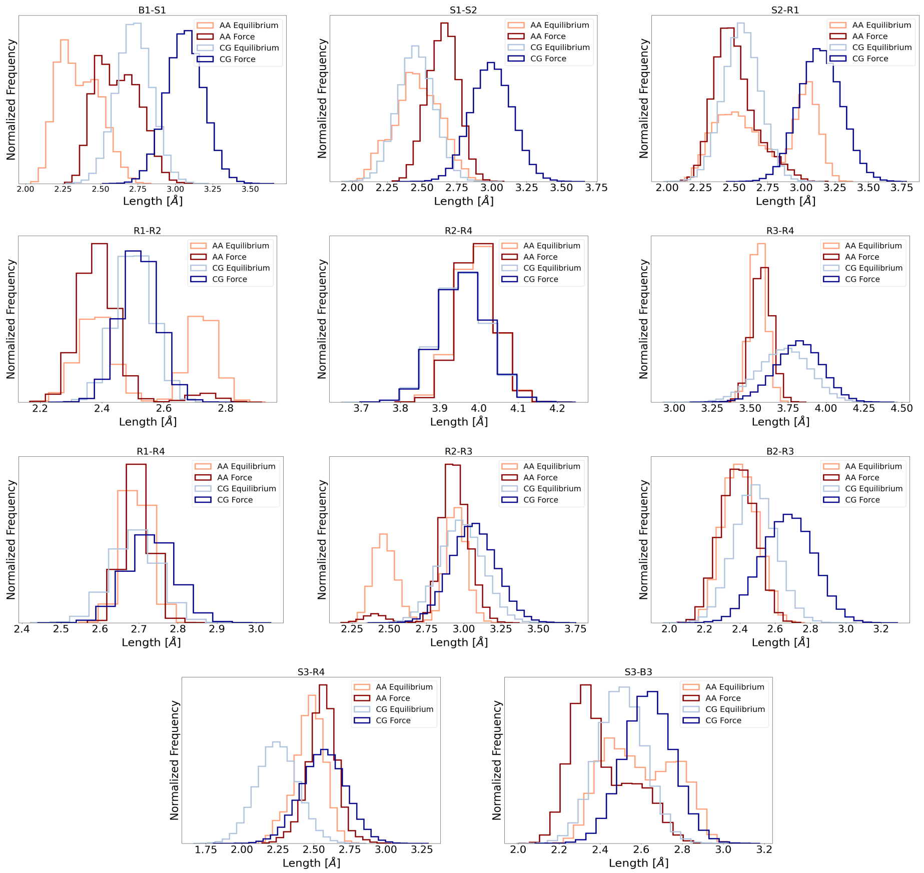

Figure S18: **Crosslink bond length distributions from the 67 nm trivalently crosslinked collagen fibril.** Trivalent crosslink bond length distributions for specific bond pairs (*B1-S1*, *S1-S2*,*S2-R1*, *R1-R2*, *R2-R4*, *R3-R4*, *R1-R4*, *R2-R3*, *S3-R4*, *S1-B3*) comparing equilibrium simulations with simulations under applied force (1000 pN). Bond labels correspond to the trivalent crosslink mapping scheme (see Fig. 2A). We observe similar trends between CG and AA reference simulations, with characteristic bond extension under applied force. However, slight deviations are visible, particularly for bonds involving the aromatic ring structure (*R1-R4*, *R2-R4*, *R3-R4*), reflecting the increased complexity of accurately representing the rigid pyridine ring geometry in the coarse-grained model.

References

[1] M. Abraham, A. Alekseenko, C. Bergh, C. Blau, E. Briand, M. Doijade, S. Fleischmann, V. Gapsys, Gaurav Garg, S. Gorelov, G. Gouaillardet, A. Gray, M. Eric Irrgang, F. Jalalypour, J. Jordan, C. Junghans, Prashanth Kanduri, S. Keller, C. Kutzner, J. A. Lemkul, M. Lundborg, P. Merz, V. Miletić, D. Morozov, S. Páll, R. Schulz, M. Shirts, A. Shvetsov, B. Soproni, D. Van Der Spoel, P. Turner, C. Uphoff, A. Villa, S. Wingbermühle, A. Zhmurov, P. Bauer, B. Hess, and E. Lindahl. GROMACS 2023 Manual. doi: 10.5281/ZENODO.7588711. URL https://zenodo.org/record/7588711.

[2] F. Grünewald, M. H. Punt, E. E. Jefferys, P. A. Vainikka, M. König, V. Virtanen, T. A. Meyer, W. Pezeshkian, A. J. Gormley, M. Karonen, M. S. P. Sansom, P. C. T. Souza, and S. J. Marrink. Martini 3 Coarse-Grained Force Field for Carbohydrates. 18(12):7555–7569. ISSN 1549-9618, 1549-9626. doi: 10.1021/acs.jctc.2c00757. URL https://pubs.acs.org/doi/10.1021/acs.jctc.2c00757.

[3] P. C. Kroon, F. Grünewald, J. Barnoud, M. van Tilburg, P. C. T. Souza, T. A. Wassenaar, and S.-J. Marrink. Martinize2 and Vermouth: Unified Framework for Topology Generation. URL https://arxiv.org/abs/2212.01191.

[4] G. Landrum, P. Tosco, B. Kelley, R. Rodriguez, D. Cosgrove, R. Vianello, sriniker, P. Gedeck, G. Jones, E. Kawashima, NadineSchneider, D. Nealschneider, A. Dalke, tadhurst cdd, M. Swain, B. Cole, S. Turk, A. Savelev, A. Vaucher, M. Wójcikowski, I. Take, H. Faara, R. Walker, V. F. Scalfani, D. Probst, K. Ujihara, N. Maeder, A. Pahl, G. Godin, and J. Lehtivarjo. RDKit: Open-source cheminformatics. https://www.rdkit.org. URL https://www.rdkit.org.

[5] A. V. Marenich, C. J. Cramer, and D. G. Truhlar. Universal Solvation Model Based on Solute Electron Density and on a Continuum Model of the Solvent Defined by the Bulk Dielectric Constant and Atomic Surface Tensions. 113(18):6378–6396. ISSN 1520-6106, 1520-5207. doi: 10.1021/jp810292n. URL https://pubs.acs.org/doi/10.1021/jp810292n.

[6] Martini Development Team. Free energy techniques tutorial. Martini Force Field Documentation. URL https://cgmartini.nl/docs/tutorials/Martini3/Free_Energy_Techniques/index.html.

[7] D. Monego, M. Brosz, J. Buck, V. Viliuga, P. Greicius, J. Jung, T. Stuehn, M. Schmies, Y. Sugita, and F. Gräter. ColBuilder: Flexible structure generation of crosslinked collagen fibrils. 41(6). ISSN 1367-4811. doi: 10.1093/bioinformatics/btaf278. URL https://academic.oup.com/bioinformatics/article/doi/10.1093/bioinformatics/btaf278/8125020.

[8] F. Neese. Software Update: The ORCA Program System—Version 6.0. 15 (2):e70019. ISSN 1759-0876, 1759-0884. doi: 10.1002/wcms.70019. URL https://wires.onlinelibrary.wiley.com/doi/10.1002/wcms.70019.

[9] F. Neese, F. Wennmohs, U. Becker, and C. Riplinger. The ORCA quantum chemistry program package. 152(22):224108, . ISSN 0021-9606, 1089-7690. doi: 10.1063/5.0004608. URL https://pubs.aip.org/jcp/article/152/22/224108/1061982/The-ORCA-quantum-chemistry-program-package.

[10] F. Neese, F. Wennmohs, U. Becker, and C. Riplinger. *ORCA 6.0 Manual*, . URL https://www.faccts.de/docs/orca/6.0/manual/.

[11] A. B. Poma, M. Cieplak, and P. E. Theodorakis. Combining the MARTINI and Structure-Based Coarse-Grained Approaches for the Molecular Dynamics Studies of Conformational Transitions in Proteins. 13(3):1366–1374. ISSN 1549-9618, 1549-9626. doi: 10.1021/acs.jctc.6b00986. URL https://pubs.acs.org/doi/10.1021/acs.jctc.6b00986.

[12] I. V. Tetko, J. Gasteiger, R. Todeschini, A. Mauri, D. Livingstone, P. Ertl, V. A. Palyulin, E. V. Radchenko, N. S. Zefirov, A. S. Makarenko, V. Y. Tanchuk, and V. V. Prokopenko. Virtual Computational Chemistry Laboratory – Design and Description. 19(6):453–463. ISSN 0920-654X, 1573-4951. doi: 10.1007/s10822-005-8694-y. URL http://link.springer.com/10.1007/s10822-005-8694-y.
