# Supplementary material for: Coarse-grained Martini 3 model for collagen fibrils
Source: Biophys J. 2025 Oct 10;125(2):581–93. doi: 10.1016/j.bpj.2025.10.012 (PMC12969027; doi:10.1016/j.bpj.2025.10.012)
Supplement: Document S2. Article plus supporting material [file mmc2.pdf]

Biophysical Journal
Article

Coarse-grained Martini 3 model for collagen fibrils

Matthias Brosz,[2,3] Johanna Buck,[1,2,3] Fabian Grünewald,[2,3] Debora Monego,[1,2,3] Jaewoon Jung,[4,5] Yuji Sugita,[4,5] Camilo Aponte-Santamaría,[1,2] and Frauke Gräter[1,2,3,*]
[1]Max Planck Institute for Polymer Research, Ackermannweg 10, 55128 Mainz, Germany; [2]Heidelberg Institute for Theoretical Studies, Am Schloss-Wolfsbrunnenweg 35, 69117 Heidelberg, Germany; [3]Institute for Scientific Computing, Heidelberg University, Im Neuenheimer Feld 205, 69120 Heidelberg, Germany; [4]Computational Biophysics Research Team, RIKEN Center for Computational Science, Kobe 650-0047, Yogo, Japan; and [5]Theoretical Molecular Science Laboratory, RIKEN Pioneering Research Institute, Wako 351-0198, Japan

ABSTRACT Collagen is a prevalent protein in the Animalia kingdom, especially in mammals. It is abundant in all connective tissue such as bone or ligaments, and thus, it is subjected to substantial mechanical forces. Cross-links play an essential role for the structural and mechanical integrity of collagen, determining its stiffness and rigidity. Until now, studies on collagen including cross-links have either been confined to fully atomistic simulations, which are computationally intensive and restrict the accessible time and length scales, or to coarse-grained descriptions that do not resolve the force response on a residue level and therefore do not consider the triple helical structure and the connectivity of cross-links. To bridge this gap, we report on the development and validation of a computational model based on the Martini 3 coarse-grained force field, in which we parametrized the fibrillar collagen structure including cross-links. We validated the model, through extensive equilibrium and nonequilibrium molecular dynamics simulations, against experimental properties and all-atom simulations. Because the type and distribution of cross-links vary with aging, we expect that this collagen model can be employed to provide insights into age-related changes in tissue mechanics and guide the development of biomimetic materials.

SIGNIFICANCE Collagen's distinctive triple helical structure and exceptional mechanical stability make it a central component of connective tissues. Understanding the role of covalent cross-links in stabilizing collagen fibrils remains a key challenge due to limitations in existing computational models, which often compromise on either resolution or system size. Here, we present a coarse-grained computational model of collagen fibrils, enabling detailed investigations of the role of cross-links and their impact on fibril properties.

INTRODUCTION

Collagen-based fibrillar structures serve dual critical roles in animals: they act as force-transmitting proteins between muscles and bones and, in this context, bear extreme mechanical loads—up to 90 MPa in a stretched Achilles tendon (1–3). As one of the most abundant proteins in the human body, collagen is widely used as a biomaterial in tissue engineering to treat burns and wounds, to serve as a biological scaffold for bone and cartilage regeneration, and to promote cell integration and proliferation in cardiovascular applications (4). The properties of collagen can be precisely controlled through its composition and cross-linking. This allows collagen-based scaffolds to be engineered to meet the diverse mechanical and structural requirements across different applications (5–7).

Collagen, which exists in several types, is most commonly found as collagen type I, consisting of stiff triple helices with persistence lengths ranging between 11 and 15 nm or between 65 and 180 nm, as measured through atomic force microscopy or dynamic light scattering, respectively (8, 9). Cross-links interconnect the triple helices, forming a substructure called a fibril. At this scale, collagen exhibits an alternating pattern of high- and low-density regions, referred to as overlap and gap zones. Each unit of gap and overlap region is a D-band and has a length of 67 nm. Several fibrils assemble together to form collagen fibers.

To uncover collagen's dynamic and mechanical properties across this hierarchical structure, computational

Submitted March 31, 2025, and accepted for publication October 7, 2025.
*Correspondence: frauke.graeter@mpip-mainz.mpg.de
Matthias Brosz and Johanna Buck contributed equally to this work.
Editor: Florence Tama.
https://doi.org/10.1016/j.bpj.2025.10.012
© 2025 The Author(s). Published by Elsevier Inc. on behalf of Biophysical Society.
This is an open access article under the CC BY license (http://creativecommons.org/licenses/by/4.0/).

simulation methods ranging from the atomistic to the mesoscopic scale have been employed. All-atom (AA) molecular dynamics (MD) simulations, on the one hand, have investigated both single collagen molecules and 67-nm-long fibrillar structures, revealing mechanisms of triple helical folding and stress distribution within cross-linked fibrils, respectively (10–15). However, they have been limited to single D-band lengths, preventing analysis of full-length (∼335 nm) microfibrils under force. To bridge the gap between molecular and fibrillar scales, Buehler et al. and others developed a mesoscopic model from AA-MD simulations of a 84-nm-long collagen molecule to investigate how cross-linking affects the stress-strain response at the fibril level (16). Vaughan further extended this model to characterize the influence of mineralization on collagen's response to mechanical load (17). Although this mesoscopic model qualitatively reproduces collagen's overall strength, it lacks a clear chemical and thermodynamic distinction between amino acids and cross-link types and does not fully resolve the triple helical structure giving rise to collagen's molecular stiffness. Additional earlier efforts to develop a coarse-grained (CG) model for the collagen triple helix were made by Gautieri, using the Martini 2 force field. Their approach parameterized bond length, bond angle, and torsion angle potentials by matching force-extension curves of small peptides, such as glycine-proline, glycine-proline-hydroxyproline, and glycine-proline-hydroxyproline-glycine, to AA-MD simulations (18). Despite these advances, extant CG models fail to capture collagen's hierarchical structure in sufficient detail, especially its triple helical conformation and cross-links. The limitations of existing AA-resolution, mesoscopic, and earlier CG models hinder our understanding of how cross-linked collagen responds to mechanical perturbation at the microscale, highlighting the need for a new CG collagen model with improved resolution and accurate helical structure.

Here, we present a CG model for collagen based on the Martini 3 force field in conjunction with a Gō model, designed to perform large-scale CG-MD simulations of cross-linked collagen microfibrils under both equilibrium and high-force conditions (19, 20). We focused on understanding the molecular mechanisms underlying collagen rupture by simulating forces comparable to those that cause failure in biological systems. Our model successfully captures the force-extension behavior observed in AA simulations and enables CG-MD simulations of large collagen microfibrils under tension. The parametrization process combines standard Martini 3 fitting techniques with parameter optimization under force, making it suitable for nonequilibrium simulations. Combined with the automated generation of Martini 3 input files for MD simulations through our extended ColBuilder server, this model enables detailed investigation of the relationship between cross-link configuration, microfibrillar structure, and mechanical load across both microscopic and mesoscopic scales (21).

MATERIALS AND METHODS

In Martini 3, each CG interaction site represents two to four heavy atoms plus their associated hydrogens, positioned at the center of geometry of the underlying AA structure. Martini 3 employs a building block principle for mapping a given AA structure to CG resolution and uses a mixed top-down and bottom-up parameterization strategy for the nonbonded and bonded interactions, respectively (19, 22). To model collagen microfibril's complex structure—composed of multiple triple helices connected by cross-links—we first parameterized individual cross-links and triple helices before assembling them into microfibrillar structures. As the divalent hydroxylysino-5-keto-norleucine (HLKNL) and trivalent pyridinoline (PYD) cross-links are among the most prevalent and well-characterized cross-links in mature collagen tissue, they were chosen as a proof of concept. This approach can readily be extended to other cross-links to assess their role in collagen structure, dynamics, and mechanics.

To find the bead types and mapping schemes of the not yet parametrized subblocks, we considered symmetry arguments, polarity, and thermodynamic calculations. We evaluated three collagen systems of increasing complexity: first a single triple helix in water spanning one 67-nm-long D-band without intermolecular cross-links, second a fibrillar assembly of the same length comprising 40 triple helices with cross-links localized at two different regions, and third a fibrillar structure of the length of a full collagen molecule (335 nm) containing 267 triple helices with 10 cross-link regions (3, 23). An overview of the details of the simulated systems can be found in Table S1.

The parameterization procedure for the CG model is outlined in Fig. 1. Specifically, we split the model into smaller building blocks and parametrized each block individually by determining the nonbonded interactions for both cross-links and the bonded interactions for the collagen triple helix. We aimed to reproduce the properties of the AA reference simulations and the experimental data with the Martini 3 simulations.

All-atom simulation protocol

Our AA simulations were based on two different atomistic force fields: the Amber99SB-ildn* force field (AMBER) for simulating the collagen triple helix and 67-nm-long cross-linked fibril and the Chemistry at Harvard Macromolecular Mechanics (CHARMM36m) force field for parametrizing cross-links through nonequilibrium MD-based thermodynamic free energy calculations (24–29). The AMBER parameters for both cross-links were derived by Zapp through density functional theory, using the B3LYP functional for geometry optimization and Antechamber to determine partial charges (14, 30, 31). The topology of the collagen fibrils was built using an earlier version of ColBuilder, a pipeline to generate simulation-ready structures and topology files for collagen molecules, including cross-links (21). Our simulation protocol began with the solvation of each system in a rectangular simulation box with TIP3P water (26). After energy minimization, we neutralized the system with counter-ions replacing some of the water molecules. For the AA simulations, two different engines were used: smaller systems (67 nm) were simulated with GROMACS, whereas longer fibrils (335 nm)—AA systems of approximately 43 million atoms including water—were simulated on the Fugaku supercomputer using GENESIS (32, 33).

We used GROMACS for simulations of cross-links (v2023), triple helices (v2023), or the D-band of a fibril (v2020) (i.e., the small and intermediate AA systems) (34–36). The solvent was equilibrated around the protein by applying a harmonic potential to position-restrain the heavy atoms of the collagen backbone with a force constant of 1000 kJ mol^{-1} nm^{-2}. Short-range electrostatic and van der Waals interactions were truncated at 1 nm using the Verlet scheme, and long-range electrostatics were treated using the particle-mesh Ewald method (37–39). The temperature was maintained at 300 K using the velocity rescaling thermostat with a time constant of $\tau_T =$ 0.1 ps, and the pressure was initially set to 1 bar. The Parrinello-Rahman

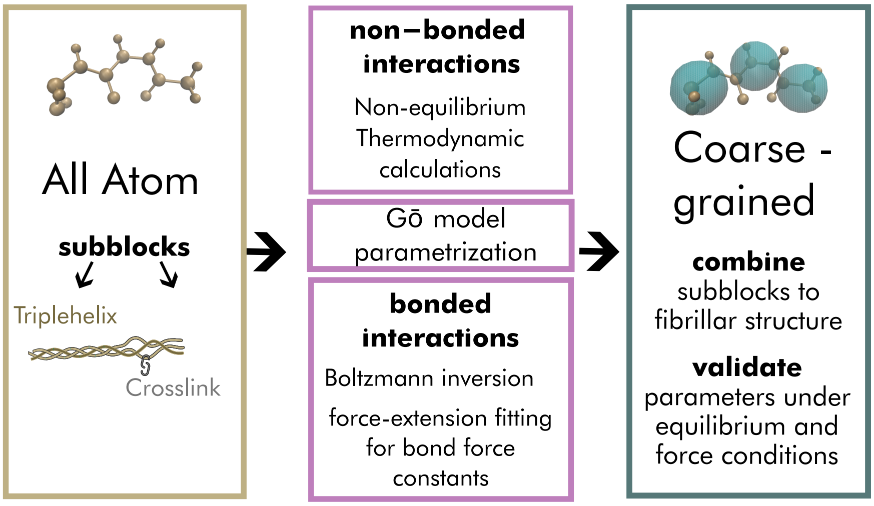

FIGURE 1 Overview of the parameterization process. The AA model is used to identify building blocks, which are independently parametrized by two main steps: the nonbonded interactions, the bead types, and the mapping schemes are found using thermodynamic calculations, symmetry, and polarity arguments. The bonded interactions are fitted via Boltzmann inversion to meet the AA data under equilibrium and under force conditions. If the building blocks are successfully determined, the two subsystems and their parameters are combined, and the structure is assembled via ColBuilder (21). To this end, the cross-linked microfibrillar structure is validated against experimental data and atomistic simulations under equilibrium and under force conditions taking into account different sized collagen systems.

barostat was used with $\tau_P = 2$ ps and a compressibility of 4.5×10^{-4} bar^{-1} (40, 41). NPT and NVT equilibration steps were performed for 10 ns each. Periodic boundary conditions were applied throughout the simulations. For the GENESIS simulations (the large 335-nm AA system), we used a 1-nm cutoff for short-range electrostatic and van der Waals interactions. The system was equilibrated in the NVT ensemble at 300 K for 10 ns, followed by 20-ns NPT equilibration using the Martyna-Tobias-Klein barostat to maintain pressure at 1 atm (42). During the equilibration steps, position restraints were gradually decreased.

After obtaining a fully equilibrated system, assessed based on the convergence of the box volume, pressure, and temperature, we performed both equilibrium simulations and simulations under constant force pulling. For the latter, we defined each pulling group as the three caps of an individual triple helix, enabling force application at the center of each molecule. Forces were applied at both ends of collagen in the z-direction, aligned with the helical axis, to mimic physiological stress conditions. The magnitude of the applied force ranged from 300 pN to 1000 pN (per triple helix). To prevent unwinding of the triple helices, torque restraints were used for all AA systems. In the GROMACS simulations, a rotational force of 2000 kJ mol^{-1} nm^{-2} was applied to the caps of the AA models, following the approach of Zapp (14, 43). As a consequence of the applied force, the collagen triple helix/fibril straightened in the pulling direction until a new equilibrium state under force was reached.

Nonbonded interactions

For parametrization of the nonbonded interactions, we divided the collagen system into two blocks: cross-links and collagen triple helix. For the triple helix, we mapped the amino acids of the AA trajectory to CG resolution before finding the Martini 3 parameters. For the cross-links, we followed the Martini 3 parametrization procedure, which involves determining partition coefficients through nonequilibrium thermodynamic calculations (44).

Nonequilibrium thermodynamic calculations. The selection of Martini 3 bead types for the divalent HLKNL and trivalent PYD cross-links was based on a comparison of partition coefficients from Martini 3 with AA simulations (19). Although we acknowledge the limitations of this parametrization approach (45), experimental validation data for these specific cross-links are, to the best of our knowledge, currently not available. The partition coefficient measures the distribution of a compound between two immiscible phases, such as octanol and water, and is determined by the solute's partitioning free energy. Using nonequilibrium MD-based free energy calculations, we estimated the free energy change to transfer each cross-link from water ($S1$) to a hydrophobic solvent ($S2$), in two thermodynamic steps, via

$$\Delta\Delta G_{S1\rightarrow S2} = \Delta G_{\varnothing\rightarrow S2} - \Delta G_{\varnothing\rightarrow S1}, \tag{1}$$

with $\Delta G_{\varnothing\rightarrow S2}$ and $\Delta G_{\varnothing\rightarrow S1}$ corresponding to the free energy associated to transfer the cross-link to (forward transition) or from (backward) the hydrophobic solvent and to and from water to vacuum, respectively. We determined the solvation free energy in water, $\Delta G_{\varnothing\rightarrow S1}$, and in the hydrophobic solvent, $\Delta G_{\varnothing\rightarrow S2}$, using the maximum likelihood method to extract equilibrium free energies from nonequilibrium transitions, based on Crooks fluctuation theorem as proposed by Shirts (46, 47). Uncertainties in the solvation free energy differences, ΔG, were quantified through bootstrapping and error propagation. To generate the coordinate and topology files for both cross-links, we used the CHARMM-Gui input generator (29, 48, 49). We relied on the standard capping for the cross-link parametrization using GROMACS and the CHARMM36m force field. Specifically, the amide group of the cross-link's peptide bond is capped with a single hydrogen ($-NH_2$) and the carbonyl group with an alcohol group ($-COOH$).

Each compound was first solvated in water and anhydrous octanol, then neutralized, and finally thermodynamically integrated (50). For the trivalent cross-link PYD, we inserted a chloride ion to compensate for the charged nitrogen N^+ on the pyridine ring, achieving a zero net charge system. To prepare for the nonequilibrium transitions, we energy minimized the system using the steepest descent algorithm and performed a NVT equilibration for 50 ns using the Berendsen thermostat at 300 K in each end state (51). After discarding the first 10 ns, we extracted 100 starting configurations from both end state ensembles.

Nonequilibrium alchemical transitions were completed using a coupling parameter λ, which was varied in both forward and reversed directions, i.e., 0→1 and 1→0, with $\lambda = 1$ corresponding to having the compound in solution and $\lambda = 0$ in vacuum. To sample the AA data, four separate replicate simulations, each consisting of 100 backward and 100 forward transitions of 2–10 ns in length, were carried out, yielding a cumulative simulation time of 4 μs. The same cumulative time was reached by 100 forward and 100 backward transitions of the CG model. The GROMACS implementation for free energy calculations was used for this purpose (32). During nonequilibrium runs, the temperature was implicitly kept constant at 300 K using a stochastic dynamics integrator, whereas the pressure was maintained at 1 bar using the stochastic rescaling barostat with $\tau_P = 5$ ns and a compressibility of 4.5×10^{-5} bar^{-1} (52, 53). A Gapsys soft-core potential was selected ($\alpha_{LJ} = 0.85$, $\sigma_{LJ} = 0.3$, $\alpha_Q = 0.3$) to prevent singularities when changing the Lennard-Jones and Coulomb interactions together (54). The work distributions associated with the forward and backward nonequilibrium transitions were finally collected to quantify the free energy

of transferring each compound between solvents, using the *pmx* package developed by Gapsys et al. (54, 55). This procedure was performed while iterating over bead types and mapping schemes to match the free energy estimates from Martini 3 to AA reference simulations. Although the Martini 3 convention is to use wet octanol as a reference solvent (19), we initially employed dry octanol for parametrization to ensure consistency with our nonequilibrium AA workflow. To account for recent advances in QM calculations and the findings of Isik et al. (45), we also compared the logP values obtained by the QM-tool ORCA (56) with SMD solvent(57), machine learned predictions (58) (reported in the supporting material) and Martini 3 thermodynamical integration. The latter were obtained with equivalent sampling as our original free energy calculation while solvating with a "wet" octanol (concentration 92:8 octanol:water) to compare against QM (SMD) predictions (19, 45, 59, 60).

Bonded interactions

We followed the standard Martini 3 framework, i.e., using AA reference data to parameterize bond lengths, angles, and dihedrals. We performed direct Boltzmann inversion by fitting a Gaussian $P(q)$ to the measured probability distributions to derive the Martini 3 force field parameters, treating each bonded term separately (61):

$$P(q) = C_q \cdot \exp\left(- \frac{1}{2} \frac{\left(q - \mu_q\right)^2}{\sigma_q^2} \right), \quad (2)$$

where q denotes a single degree of freedom (bond distance r_{ij}, bond angle θ_{ijk}, or torsion angle ϕ_{ijkl}), and μ_q, σ_q^2, and C_q are the mean, variance, and amplitude of the fitted Gaussian, respectively. For the Boltzmann inversion, we assumed each degree of freedom to be independent in the canonical ensemble, following a Boltzmann distribution (61):

$$P(q) = Z^{-1} \cdot \exp\left(- \beta \cdot V^{CG}(q) \right), \quad (3)$$

where Z is the partition function for the ensemble, and $V^{CG}(q)$ is the Martini 3 potential. By comparing the mean μ_q and variance σ_q^2 of the Gaussian function (Eq. 2) to the Boltzmann distribution (Eq. 3), we obtained the equilibrium bond value (μ_q) and an initial force constant under equilibrium (yielding proper σ_q) of each bonded term in the Martini 3 force field.

We performed inverse Boltzmann fitting separately for each of the two building blocks. For the triple helix parameters, we performed AA equilibrium simulations of three 67-nm-long collagen molecules in water. These three triple helices were obtained by splitting the 300-nm-long collagen molecule into five parts: two telopeptide regions (at each end) and three central 67-nm segments. The telopeptide regions were discarded, and the three centered segments were chosen as parametrization configurations. Each segment was run for 100 ns, before applying a center-of-geometry-based mapping scheme to the protein backbone atoms. We then extracted bond lengths, bond angles, and torsion angles between backbone beads (BBs), using their probability distributions as our major parametrization targets while considering their helical shape, particularly the experimental results for the triple helix (62–65). Additionally, we conducted fitting under force to fine-tune bond force constants. For both cross-links, we also determined the bonded terms with Boltzmann inversion and optimized the bond force constants during simulations under force.

Gō model. The standard Martini 3 force field does not inherently preserve the helical structure of the collagen molecule. To address this, we implemented a Gō model, following the work of Souza, which adds a Lennard-Jones potential applied to virtual sites that move with the protein BBs (20, 66). Specifically, we restricted the Gō-like potentials to each triple helix, that is, to intrahelical interactions, as shown in Fig. S9. Note that the influence of the Gō model's potential well depth on the elongation under high forces is negligible. For this reason, we opted to rely on the default parameters, including a potential well depth of $\epsilon_r = 9.414$ kJ mol^{-1} (67). To generate the topology of the collagen fibrils, we relied on ColBuilder (21). More details about the design of the Gō model and the generation of the fibrillar structure can be found in the supporting material in Topology generation.

Coarse-grained simulation protocol

For the CG simulations, we followed the standard Martini 3 parameter setup for the neighbor list, electrostatic, and van der Waals interactions (68). Specifically, the neighbor list was defined with a cutoff at 1.35 nm, whereas the reaction-field algorithm cutoff the electrostatics at 1.1 nm with a relative permittivity of $\epsilon_r = 15$ (69, 70). These parameters follow the Martini 3 simulation guidelines available at https://www.cgmartini.nl. All simulations were performed using GROMACS (v2023) (32).

For simulating a single collagen triple helix, we first solvated the structure in a rectangular simulation box using Martini 3 water beads. We then neutralized the system with counter-ions and performed energy minimization using the steepest descent algorithm. Temperature stabilization followed for 5 ns at 300 K using the velocity rescaling thermostat with $\tau_T = 1$ ps. Pressure equilibration was achieved using the Berendsen barostat, maintaining 1 bar for 3 ns with a compressibility of 3×10^{-5} bar^{-1} and $\tau_P = 12$ ps. In the production run, we set the pressure to 1 bar using isotropic pressure coupling with the Parrinello-Rahman barostat. For higher-order crystal structures, we followed similar energy minimization steps but implemented a longer multistep NVT and NPT equilibration procedure (see Fig. S10). For the 335-nm-long microfibril, we inserted additional water beads, in several insertion steps between NPT runs, to ensure adequate solvation. After achieving equilibrated CG collagen molecules and fibrils under isothermal-isobaric conditions, we performed pulling production runs. Although the setup for the CG system matched the AA conditions, for the CG model, we decided not to use torque restraints because the force constants we applied in the AA simulations were too low for the CG simulations, and we had no systematic basis for choosing appropriate force constants. Also, these restraints were unnecessary for the 335-nm-long CG collagen fibril due to its structural stability. The trajectory analysis and structural properties were computed using the protein helices analysis modules from MDAnalysis 2.5 (71, 72). For other properties, we used GROMACS tools (32). For equilibrated properties, we discarded the initial 20 ns, 30 ns, or 150 ns of trajectories, depending on the system size.

RESULTS

Mapping of the cross-links and caps

Specifically, for the HLKNL cross-link, we developed a symmetrical mapping relative to the central secondary amine group (Fig. 2 *A*); a small *SP1d* bead represents the hydrogen donor characteristics of this amine group. Standard four-to-one mapping was applied to the 1-propanol and 1-propanone groups, using a polar *P1* bead for the alcohol and an intermediate hydrophilic *N6a* bead for the ketone group. The aromatic structure of the PYD cross-link required small and tiny beads (Fig. 2 *A*, *bottom*). The central pyridine ring was represented by four tiny beads: a *TQ2p* bead for the ring nitrogen, a *TP1q* bead for the phenol group, a *TC6q* bead for the benzene moiety, and a *TC4* bead at the ortho-bridging carbon that connects to the C_α of the lysine residue (*LY2*). This rhombic arrangement (*R1*, *R2*, *R3*, *R4* in Fig. 2 *A*, *bottom*), preserves the structural rigidity of the aromatic ring. The ring structure could only withstand such high forces with this rhombic structure of covalent bonds. Each of these four bonds was

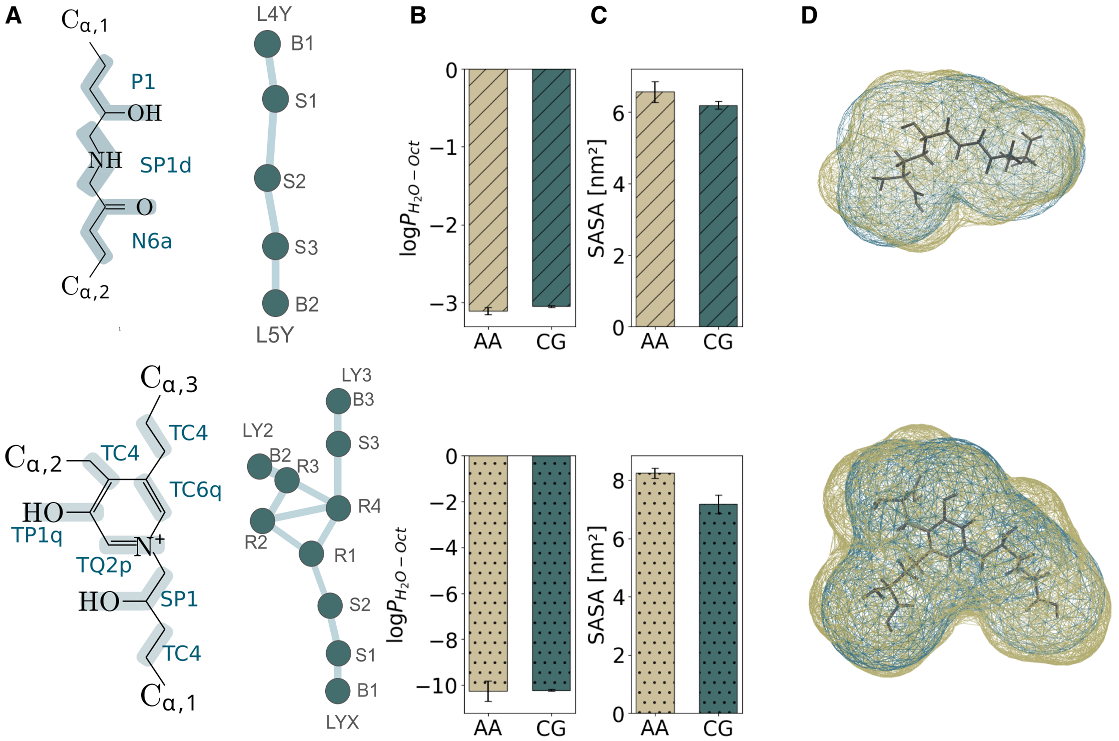

FIGURE 2 Mapping and bead type selection of the collagen cross-links with Martini 3. The divalent HLKNL (*top*) and the trivalent PYD (*bottom*) cross-link were considered. (*A*) Mapping and bead types are shown, highlighting the atoms grouped in each Martini 3 bead type (*left*) and the connectivity with an alpha-numeric numbering (*right*). (*B*) Octanol/water log P_{H_2O-Oct} values were obtained from AA and CG simulations to validate the proper thermodynamic partitioning of the cross-links. (*C*) Solvent-accessible surface area (SASA) and (*D*) Connolly surface area from AA simulations (*ocher*) and CG simulations (*dark turquoise*) confirmed the proper size of the CG cross-links.

found to be crucial for maintaining ring stability under pulling forces, preventing the separation of the two arms of the trivalent cross-link attached to the same triple helix. The nontrivial bonds (*R2–R4* and *R3–R4*) cannot be straightforwardly replaced by constraints. Despite the planarity of the ring, we decided to not define a dihedral angle for this degree of freedom, since this led to high numerical instabilities in the simulations under force. Moreover, similar rhombic arrangements have been successfully used for mapping aromatic systems in CG models (44), validating our approach. For the beads attached to the ring, we used angles to maintain the system's geometry. To account for the protonated pyridine nitrogen, we distributed partial charges across the tiny beads, assigning $q = 0.7$ e, $q = 0.2$ e, and $q = 0.1$ e to the *TQ2p*, *TP1q*, and *TC6q* bead, respectively, following the AMBER charge distribution. We applied q-labeled beads even for charges below 0.25 to emphasize the delocalization of charges. This choice is not in line with the Martini 3 guidelines, but this bead typing yielded good agreement with the partitioning behavior from AA simulations. For the C_α connections to the helices, we selected moderate polar *TC4* beads showing good agreement with the partitioning data (supporting material: Fig. S6). The link between the charged nitrogen and $C_{\alpha,1}$ was represented by a polar *SP1* bead.

We based our bead type selection by comparing partition coefficients for both cross-links from Martini 3 with AA simulations (70). The partition coefficient of each compound was determined in an octanol/water system using nonequilibrium MD-based free energy calculations. The transfer-free energies obtained through this method are $\Delta\Delta G_{W\rightarrow O} = -19.06 \pm 0.07$ kJ mol^{-1}, for the divalent cross-link, and $\Delta\Delta G_{W\rightarrow O} = -60.63 \pm 0.24$ kJ mol^{-1}, for the trivalent cross-link. From these transfer-free energies, the partition coefficients were obtained via Eq. 1. We observed good agreement between the partition coefficients for the divalent and trivalent cross-links from Martini 3, as visualized in Fig. 2 *B*, showing that the chosen bead types properly capture the thermodynamic partitioning of the cross-links. We further validated our bead type selection by comparing partition coefficients obtained by QM(SMD)/machine-learned prediction methods for both cross-links (70). The logP values are summarized in Table 1 (computational details for comparative methods are provided in the supporting material). For the neutral HLKNL cross-link, AA-dry and CG-dry closely agree, showing that the chosen bead types properly capture the thermodynamic partitioning of the cross-links. When wet octanol is used instead, CG-wet and QM(SMD) converge within uncertainty, indicating that our CG mapping is robust across solvent definitions. For

TABLE 1 Comparison of logP values across different methods

Cross-link	AA-*dry*	CG-*dry*	CG-*wet*	QM(SMD)
HLKNL	−3.11 ± 0.05	−3.05 ± 0.01	−3.50 ± 0.69	−2.9
PYD	−10.26 ± 0.44	−10.22 ± 0.04	−5.46 ± 0.76	−4.3

All partition coefficients were obtained via Eq. 1 by first estimating the free energy. The "AA-dry" and "CG-dry" partition coefficients were used for parametrization, whereas the "CG-wet" and QM(SMD) columns were obtained for further validation. The CG values agree well with their respective reference (AA or QM).

the charged PYD cross-link, AA-dry and CG-dry yield artificially high values, reflecting the ill-defined nature of logP for charged species. By contrast, CG-wet and QM(SMD) agree well with empirical predictions. This is consistent with observations from the SAMPL7 blind challenge, where charged molecules showed large method-dependent deviations (73).

These comparisons demonstrate that our parametrization strategy yields transferable bead assignments: although AA-dry was used for initial parametrization, the close agreement between CG-wet and QM(SMD) values validates our approach across different solvent definitions. Given that QM(SMD) approaches are increasingly considered state-of-the-art for logP predictions (73), the agreement of the corresponding values confirms our bead choices. We chose to fit the model to the partitioning data as the prior target, trying also to be as consistent as possible with the Martini 3 guidelines. However, in some cases, trade-offs arose where simultaneously fulfilling both requirements was not possible. We consider the presented mappings to be a reasonable compromise while acknowledging that alternative bead choices may also reproduce the data well and could be more suitable for other applications.

Parametrization of bonded terms for cross-links

We followed the previously mentioned AA simulation protocol under equilibrium conditions to obtain an equilibrated ensemble for each cross-link in water. We computed bond distances, bond angles, and torsion angles between cross-link beads from the mapped trajectory and analyzed their probability distributions. We then adjusted the CG probability densities to match the mapped AA distributions, following the standard bottom-up procedure for parametrizing new molecules with Martini 3 (19, 74). To reproduce the atomistic structure, the lengths of bonds between Martini 3 beads were rescaled based on solvent-accessible surface area (SASA) comparisons with AA simulations. The adjustments are typically small (on the order of 0.1–0.2 nm) but essential for ensuring that the CG model reproduces the correct molecular volumes and packing behavior (details in Fig. S7). As expected, the SASA value for the divalent cross-link from Martini 3 simulations (6.2 ± 1.1 nm^2) agrees with the estimates from AA simulations (6.5 ± 0.3 nm^2). Due to the challenging geometry of the trivalent cross-link, we accepted larger deviations for this cross-link, with a value of 8.24 ± 0.2 nm^2 for Martini 3 and 7.04 ± 0.3 nm^2 for AA simulations (Fig. 2 *C*). Further details on the Martini 3 force field parameters for both cross-links can be found in Tables S4–S6.

To examine structural differences at the molecular level, we analyzed Connolly surfaces derived from AA and CG simulations (Fig. 2 *D*). Although the agreement is not perfect, the CG model reasonably reproduced the HLKNL cross-link Connolly surface from the AA structure. Similar agreement was observed for the Connolly surface of the trivalent PYD cross-link. The differences in size likely arise from the inherent loss of fine-grained atomic details upon coarse-graining. These differences become negligible in the larger fibril structure, where bonded interactions dominate under applied force.

Parametrization of bonded terms for triple helical backbone

The collagen molecule exhibits a triple helical structure that confers unique mechanical properties through both intra- and interhelical interactions. These interactions are critical for understanding the molecule's behavior under physiological conditions and its role in tissue mechanics. Therefore, the parametrization of bonded terms is of particular importance. For the backbone structure, we first assigned bead types based on the Martini 3 parameters for amino acids. We combined conditional probability distributions with Boltzmann inversion to reproduce the triple helix symmetry and shape. Bond length analysis revealed a bimodal distribution (Fig. 3 *A*), indicating that backbone bead distances are sequence specific rather than uniform. We fitted this complexity using a bimodal Gaussian distribution—comprising two Gaussian functions—which allowed us to estimate the mean and variance of each distribution separately, thereby accounting for the nonequidistance of the mapped collagen backbone.

Based on our analysis, the first Gaussian, representing the lower peak of the bimodal distribution, was centered at $\mu_{b,1} = 0.318$ nm, with $\sigma_{b,1} = 0.010$ nm, whereas the second was located at $\mu_{b,2} = 0.354$ nm, with $\sigma_{b,2} = 0.013$ nm. Sequence analysis identified hydroxyproline (HYP) and proline (PRO) as primary contributors to the first peak, consistent with their helix-stabilizing role. We therefore implemented two bond potentials: one for general amino acid pairs and another for HYP/PRO-X combinations (where X is any amino acid). To decide which bond distance to assign to each amino acid type, we first determined the intersection point of the two Gaussian distributions at $x_0 = 0.329$ nm, based on the overlapping probability densities. We computed backbone bead distances and applied this threshold as a filter. Next, we assigned probabilities to each residue type based on whether its backbone bead was

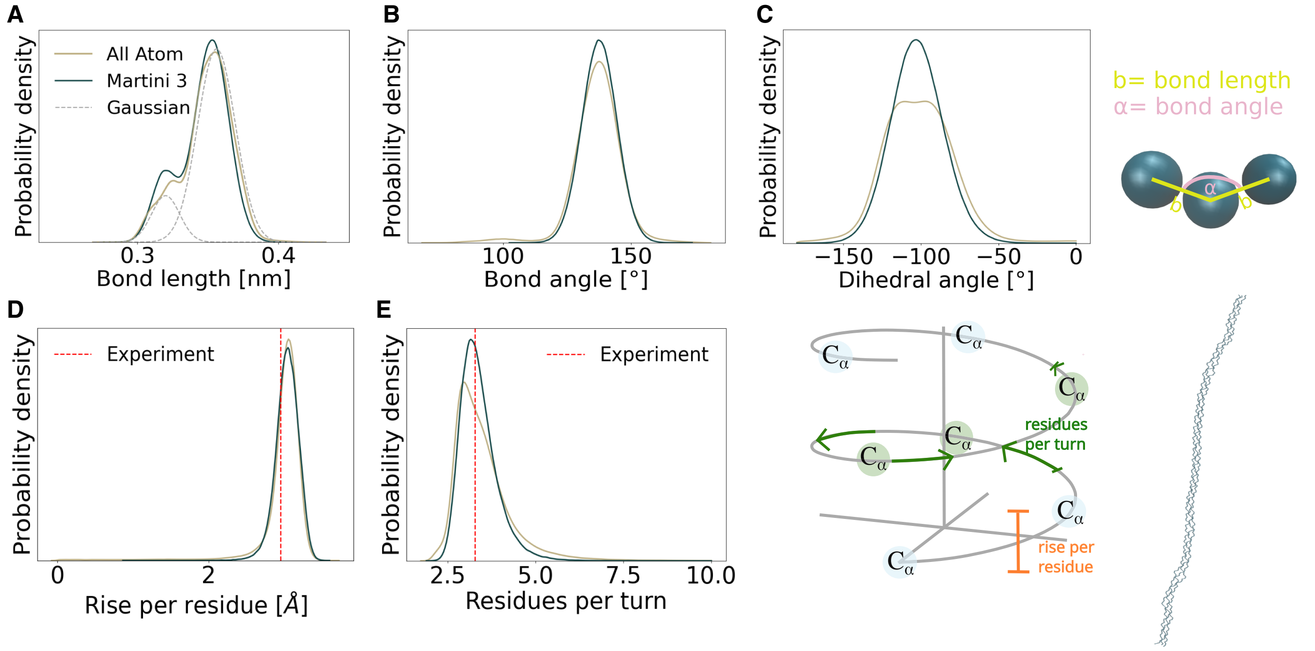

FIGURE 3 Parametrization of bonded interactions and shape of collagen triple helix under equilibrium conditions. Mapped AA trajectory (simulated in equilibrium, *ocher*) versus CG trajectory (*dark turquoise*). (*A*) Bond length including the two Gaussian fits (*gray*), (*B*) bond angles, (*C*) dihedral angles, (*D*) rise per residue, and (*E*) residues per turn (*right*). All figures show qualitative agreement of the two different setups. Additional properties (local bends and twist of the helix) can be found in the supporting material (Fig. S2).

at the start (BB_1) or end (BB_2) of the bond. The detailed probability assignment process and validation are available in Fig. S2. To achieve better agreement with the AA simulation peaks, we optimized the model's accuracy by marginally adjusting the bond lengths to $\mu_{b,1} = 0.320$ nm and $\mu_{b,2} = 0.356$ nm. This dual-potential approach was essential to capture the sequence-specific structural variations in the collagen backbone. We applied equivalent procedures for bond angle and dihedral angle parametrization, both of which exhibited unimodal probability distributions (Fig. 3 *B* and *C*). The parameters derived through our systematic optimization approach are presented in Table 2.

TABLE 2 Martini 3 force field parameters

Bonded terms	Equilibrium value [nm or °]	Force constant [kJ mol^{-1} nm^{-2} or kJ mol^{-1}]
BB_1–BB_2[a]	0.356	18,000
BB_1–BB_2[b]	0.320	34,000
BB_1–BB_2–BB_3	138	152
BB_1–BB_2–BB_3–BB_4	76	17

Force constants were derived from simulations under constant force to capture the mechanical response accordingly. Of note, the bond length value is chosen to be marginally larger than the Boltzmann inversion suggested since the peaks of the Gaussian distributions from Martini 3 tend to be at smaller values than the AA data.

[a]Bond length potential for all amino acid sequences, except those starting with a proline-like type of amino acid.

[b]Bond length potential for amino acid sequences, such as HYP/PRO-X.

We validated the parameters by comparing two key observables: rise per residue (characterizing axial extension) and residues per turn (describing radial geometry) (Fig. 3 *D* and *E*) (62, 63, 65, 75, 76). The rise per residue was slightly higher than the experimental value of 0.290 nm measured by x-ray fiber diffraction (0.307 nm for AA, and 0.304 nm for CG simulations) (62, 63). Both simulations underestimated residues per turn compared with experiments. The AA simulations show a peak at 2.96 residues per turn (10% below experimental values), whereas CG simulations exhibit a peak at 3.15 residues per turn (5% below experimental measurements with 3.28 residues per turn) (64, 65, 75). These comparisons demonstrate that our CG collagen model successfully captures both the axial and radial degrees of freedom of each helical strand and largely reproduces the shape of the collagen triple helix.

Equilibrium parameters did not accurately capture mechanical properties under applied force. Bond force constants were identified as the primary determinant of extension behavior of a triple helix, and thus also of fibrillar collagen structures, under force. As a result, we fine-tuned the force constant of the bond length potentials under force to capture the appropriate force response. Therefore, we performed simulations of a single triple helix under a range of pulling forces (300–1000 pN) using both AA and CG models (Fig. 4 *A*). We systematically explored the interplay between the Gō model and bonded parameters by testing different potential well depths under varying mechanical loads (see supporting material Fig. S9). Although the Gō

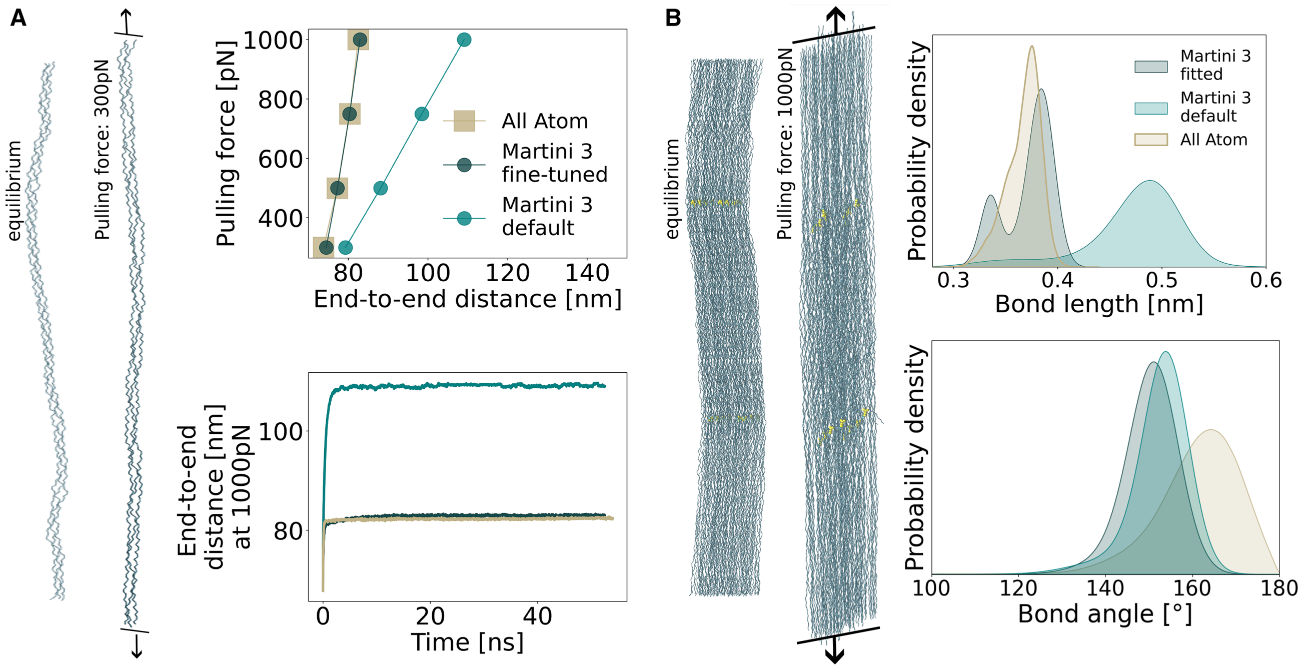

FIGURE 4 Fitting and validation of the obtained backbone-bonded parameters in simulations under force. (*A*) Triple helix under force: visualization of a triple helix in equilibrium and under force conditions (*left*). Converged values of the end-to-end distance of the triple helix simulated under forces from 300 pN to 1000 pN are shown for the adjusted bonded parameters, "Martini 3 fine-tuned" (backbone force constants of 18,000 kJ mol^{-1} nm^{-2}/ 34,000 kJ mol^{-1} nm^{-2}), and for default Martini 3 (force constant of 4000 kJ mol^{-1} nm^{-2}) and AA simulations for comparison (*right, top*). A representative timeseries at 1000 pN applied pulling force (*right, bottom*). (*B*) A 67-nm-long, trivalently cross-linked fibril under force. Structure under equilibrium and force conditions, where the yellow dots represent the cross-links (*left*). Bond length and angle width distributions of the fibrillar structure (*right*).

model well depth significantly influences the molecular response below 300 pN (entropic force regime), this effect disappears above 1 nN. Rather, at such high forces, bond stretching dominates the mechanical response. This confirms that optimizing bonded parameters, rather than the Gō model epsilon, is the appropriate approach for reproducing collagen mechanics under high stress. Increasing bond stiffness successfully matched the AA response. For this, we increased the bond force constants to 18,000 kJ mol^{-1} nm^{-2} and 34,000 kJ mol^{-1} nm^{-2}. For comparison, we also evaluated the standard Martini 3 force field with its default bond force parameter of 4000 kJ mol^{-1} nm^{-2}, which showed significantly less accurate reproduction of the mechanical response.

We tested our model by evaluating the mechanical response of a 67-nm-long collagen fibril, cross-linked with both divalent (Fig. S14) and trivalent bonds (Fig. 4 *B*). To analyze backbone-bonded parameters under force, we mapped AA trajectories to CG resolution and compared distributions at the Martini bead level. Specifically, we examined the distributions of bond lengths and bond angles between BBs subjected to a force of 1000 pN applied at each triple helix. Under force, the bimodal bond length distribution observed in AA equilibrium simulations (Fig. 3 *A*) transforms into a single broad distribution, whereas the Martini 3 model maintains a distinct double-peak character. Despite this difference in distribution shape, stiffening the bonds in our model significantly improved the overall mechanical response of the fibril when compared with the AA data.

For the trivalently cross-linked fibril, the bond angles under force revealed partial deviations between the CG and AA simulations (Fig. 4 *B*). The fine-tuned CG model exhibits more restricted angular variations compared with the mapped AA trajectory, where the backbone angles show greater flexibility. Although this discrepancy could be partially attributed to the loss of information upon mapping from atomistic to CG resolution, we deliberately maintained higher angle force constants to preserve the characteristic triple helical structure, prioritizing biological relevance over exact flexibility matching. The trade-off between exact reproduction of AA flexibility and maintenance of helical stability reflects our focus on capturing the fundamental structural properties of collagen in the CG model. Future work could explore more sophisticated parameter balancing approaches.

Force-extension dynamics

Finally, to evaluate the applicability of our model for large-scale simulations, we considered a 335-nm-long microfibril of collagen. Such systems consisted of ~43 million atoms (at AA resolution) and 6.3 million beads (at CG resolution), highlighting the relevance of developing CG models for studying these types of systems. We

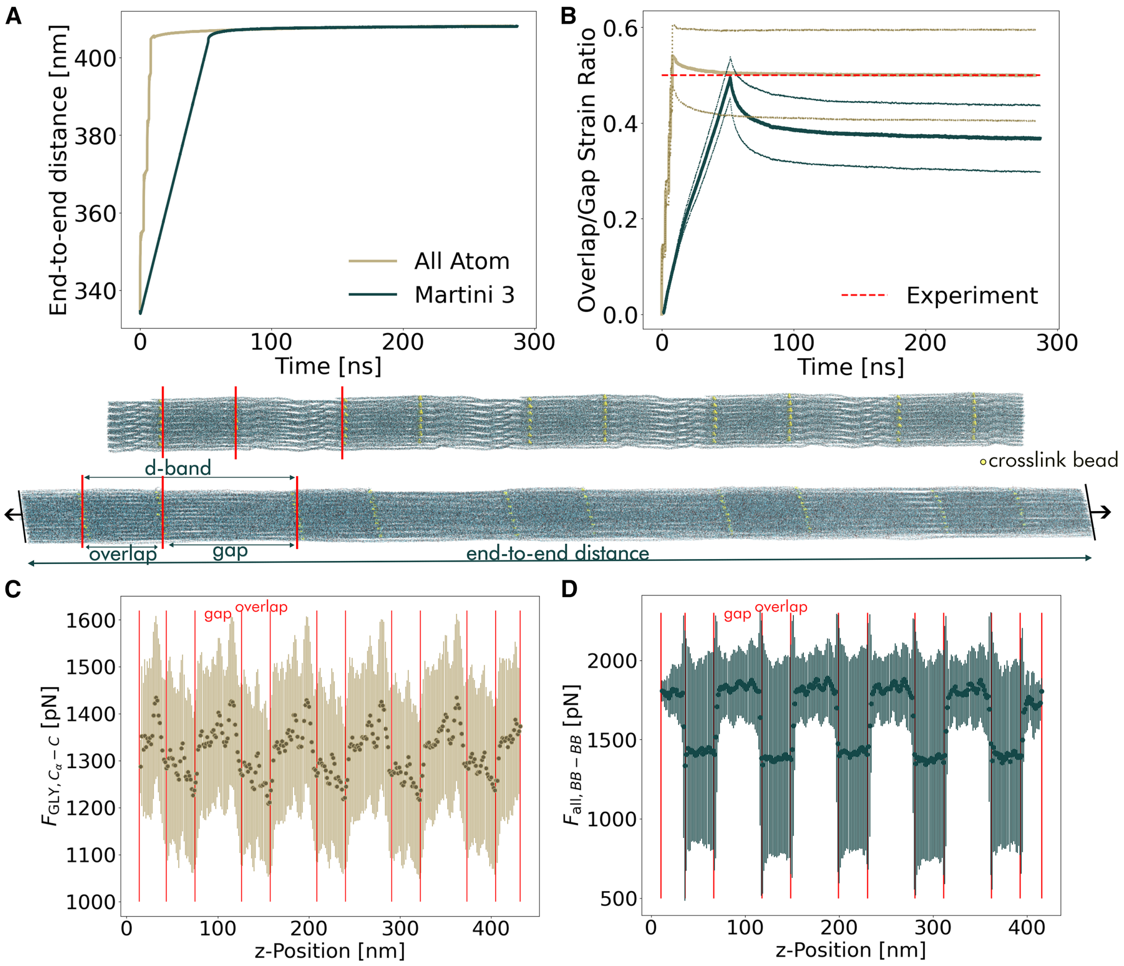

FIGURE 5 Force-extension dynamics of the 335-nm-long microfibril of collagen. (*A*) Timeseries of the end-to-end distance obtained from AA and Martini 3 simulations. (*B*) Overlap-gap strain ratio time series with the corresponding uncertainty: since the cross-link positions vary, we show the propagated error of the uncertainty of the overlap and gap length. (*C* and *D*) Detailed analysis along the *z*-axis of the fibril: binned mean of 300 bins evenly distributed along the *z*-axis and shows their *z*-position. For comparison, we show the forces between the C_α-*C* atoms in glycine in the AA model and the forces within all backbone beads (BBs) in the CG model.

examined the force-extension dynamics of this fibril under applied force, applying a pulling force of 1000 pN. For this case, we chose the trivalent PYD cross-link, because its challenging parametrization made it an especially rigorous test case for model validation.

The end-to-end distance under 1000 pN force demonstrates that both AA and CG models converge to the same elongation, showing a similar force response (Fig. 5 *A*). To examine the structural deformation in detail, we calculated the average overlap/gap strain ratio $\langle \epsilon_{overlap,t} \rangle / \langle \epsilon_{gap,t} \rangle$ according to

$$\frac{\langle \epsilon_{overlap,t} \rangle}{\langle \epsilon_{gap,t} \rangle} = \frac{\langle l_{overlap,t} - l_{overlap,0} \rangle}{\langle l_{gap,t} - l_{gap,0} \rangle}, \quad (4)$$

where $l_{overlap,t}$ and $l_{gap,t}$ are the length of the overlap and gap region at time *t*, respectively. The dotted lines in Fig. 5 *B* represent the propagated uncertainty of the gap and overlap measurements, arising from variations in cross-link positions within the fibril. We computed gap and overlap elongations as averages $\langle \ldots \rangle$ across all complete segments (four and five, respectively) in the fibril. The AA data match the experimental value of 0.5 very closely. The CG data converge to a value around 0.4, indicating a greater stiffness of the overlap region relative to the gap region. Thus, our Martini 3 model overestimates this difference and finds the overlap region to be slightly stiffer compared with AA. We attribute this difference to the loss in structural details at the cross-link region, which slightly changes cross-link tilting and extension under force. This deviation highlights the critical role of cross-links as structural connectors in determining mechanical properties. Nevertheless, the agreement between our CG model and the AA data at this large scale (~300 nm) is excellent.

To further characterize the mechanical response, we analyzed the force distribution along the fibril's *z*-axis. For the AA model (Fig. 5 *C*), we analyzed C_α-*C* bond forces in glycine residues ($F_{GLY,C_\alpha-C}$) as representative backbone

markers. For the Martini 3 model, we examined the forces $F_{all,BB_i-BB_{i+1}}$ between all BBs (Fig. 5 *D*).

Although our force distribution analysis provides valuable insights into the mechanical response along the fibril axis, we acknowledge that this approach has limitations in capturing the full complexity of interhelical interactions. Specifically, hydrophobic interactions between collagen chains within the fibril may not be fully represented by this force-based analysis method. At the high pulling forces used in our simulations (1000 pN), mechanical response is dominated by covalent interactions (primarily cross-link mechanics and backbone bond stretching) rather than weaker noncovalent interactions such as hydrophobic effects. Additionally, the inherent smoothing of the free energy landscape in CG models reduces friction between triple helices, potentially leading to enhanced elongation compared with AA systems (77). This effect, combined with differences in load distribution between individual chains during mechanical deformation, represents a fundamental challenge in CG modeling that requires careful analysis. In the case of collagen, we expect that the interhelical water layer both at AA and CG levels reduces friction due to relative helix sliding. A complete characterization of these interaction effects between the triple helices is an important area for future model development.

The CG model showed a repeating force pattern across all regions, with consistently lower average forces in overlap regions compared with the gap regions. Furthermore, CG simulations showed wider force distributions in overlap regions, whereas the AA model exhibited uniform uncertainty across both regions. Notably, the AA data reveal higher forces at gap-to-overlap transitions, a feature not captured in the Martini 3 model. This discrepancy likely results from a combination of the reduced precision of the mapping procedure and the intrinsically smoothed nature of the CG force field. Despite these differences, recapitulation of the repeating force pattern enabled the CG model to reproduce the correct force elongations.

DISCUSSION

We have developed and validated a CG model for collagen using the Martini 3 force field. Our model enables the detailed investigation of cross-link interactions with triple helical structures, significantly improving upon previous approaches (17, 18). We have integrated our model into the framework of ColBuilder, enabling large-scale CG-MD simulations of collagen fibrils and a comprehensive analysis of how cross-link density and types influence collagen dynamics (21).

The unique structural characteristics of collagen presented distinct challenges for parametrization. Specifically, we encountered situations, when parameterizing the cross-links, where we had to balance adhering to the Martini 3 guidelines with reproducing the AA data, a trade-off that should be carefully considered in future development and application of the presented model. Also, the triple helical coil and GLY-X-Y pattern, where X is typically a PRO and Y a HYP, required refitting approaches. Despite these challenges, our model successfully reproduced key collagen-specific observables. To maintain the helical secondary structure, we implemented a Gō model. We observed that small systems, particularly single triple-helix ends, can exhibit partial unwinding under very high force. This unwinding behavior was not observed in biologically sized fibrillar systems (of several hundreds of nanometers like the one studied here), suggesting that system size and cross-linking provide natural structure stabilization. Although the current Martini 3 implementation cannot fully maintain the helical shape through applied potentials alone, that is, without the Gō model or similar ways of explicit secondary structure stabilization, future versions may offer new approaches for parametrizing helical proteins.

A key methodological contribution of our work addresses the challenge of out-of-equilibrium, stretched coarse-graining with Martini 3, for which no established protocols currently exist. Our observation that Martini 3 bonds require stiffening led us to prioritize bond potential fitting over angle potentials. We propose that bond forces are crucial for accurate force/pulling simulations, an insight that may be valuable for parameterizing other CG scales, such as the CALVADOS model (78), under force. We demonstrated feasibility of simulating exceptionally large systems (43 million atoms) on the Fugaku supercomputer at the Riken center. On standard high-performance computing clusters, we observed substantial computational efficiency with approximately 16-fold speed-up comparing the AA (0.31 ns/day/cpu) to the Martini 3 setup (5.0 ns/day/cpu). This enhanced performance enables extensions to the model, including system sizes that overcome boundary effects (Fig. S12) and enable better comparison with biological experiments and investigation of the effect of advanced glycation end products in collagen fibrils.

The Martini 3 force field's coarse-graining approach offers several advantages beyond its computational efficiency. Although maintaining residue-level resolution, it provides more detailed structural representation than coarser models such as colbreaker (79) or the model from Depalle (16). Furthermore, our model's compatibility with backmapping techniques creates opportunities for predicting reactivity with chemical detail, e.g., through reactive-MD pipelines like KIMMDY (14, 80). This capability will allow detailed analysis of rupture events and force distribution patterns in systems of 335-nm length and beyond.

DATA AND CODE AVAILABILITY

The force field parameters are available via the ColBuilder GitHub repository (github.com/graeter-group/colbuilder). Simulation data produced can be found at https://doi.org/10.17617/3.MF8BBT.

ACKNOWLEDGMENTS

This work was supported by the Klaus Tschira Foundation, the Max Planck Society, and the European Research Council (ERC) (grant number 101002812) and by a Research Grant from HFSP (ref.-no: RGP025/2024) and the award DOI: https://doi.org/10.52044/HFSP.RGP0252024.pc.gr.194178. F. Gräter acknowledges funding from the Deutsche Forschungsgemeinschaft (DFG, German Research Foundation) under Germany's Excellence Strategy for the Excellence Cluster "3D Matter Made to Order" (EXC-2082/1–390761711). F. Grünewald acknowledges funding from the Klaus Tschira Stiftung gGmbH (Independent PostDoc). D.M. acknowledges funding from the Marie Skłodowska-Curie Actions Individual Fellowship (grant number 101151862). The authors acknowledge support by the state of Baden-Württemberg through bwHPC for computational resources on the bwForCluster Helix and the German Research Foundation (DFG) through grant EXC-2082/1-390761711. Y.S and J.J acknowledge the MEXT program for promoting research on the supercomputer Fugaku (JPMXP1020200101) and MEXT program for Big-data-driven bio/synthetic polymer science to create absolutely circular materials (JPMXP1122714694) and Data-Driven Research Methods Development and Materials Innovation Led by Computational Materials Science (JPMXP1020230327).

AUTHOR CONTRIBUTIONS

M.B. and J.B. parametrized the model with Martini 3 and performed the simulations as well as the analysis. F. Grünewald and C.A.-S. helped with understanding, using, and tuning the Martini 3 force field to the intended model. M.B. and D.M. built ColBuilder, which was used to generate the simulated system. J.J. and Y.S. implemented and performed the all-atom molecular dynamics simulations on Fugaku. J.B., D.M, M.B., and F. Gräter wrote the manuscript with input from all authors. F. Gräter conceived and supervised the project.

DECLARATION OF INTERESTS

No competing interest is declared.

SUPPORTING MATERIAL

Supporting material can be found online at https://doi.org/10.1016/j.bpj.2025.10.012.

REFERENCES

1. Komi, P. V. 1990. Relevance of in Vivo Force Measurements to Human Biomechanics. *J. Biomech.* 23:23–34. https://linkinghub.elsevier.com/retrieve/pii/0021929090900385.
2. Ban, E., B. R. Freedman, …, R. G. Wells. 2023. Editorial: The Mechanobiology of Collagen Remodeling in Health and Disease. *Front. Mech. Eng.* 9:1211250. https://www.frontiersin.org/articles/10.3389/fmech.2023.1211250/full.
3. Fratzl, P. 2008. *In* Collagen: Structure and Mechanics, an Introduction, P. Fratzl, ed Springer US, pp. 1–13. http://link.springer.com/10.1007/978-0-387-73906-9_1.
4. Copes, F., N. Pien, …, D. Mantovani. 2019. Collagen-Based Tissue Engineering Strategies for Vascular Medicine. *Front. Bioeng. Biotechnol.* 7:166. https://www.frontiersin.org/article/10.3389/fbioe.2019.00166/full.
5. Majid, Q. A., A. T. R. Fricker, …, I. Roy. 2020. Natural Biomaterials for Cardiac Tissue Engineering: A Highly Biocompatible Solution. *Front. Cardiovasc. Med.* 7:554597. https://www.frontiersin.org/articles/10.3389/fcvm.2020.554597/full.
6. Gurumurthy, B., and A. V. Janorkar. 2021. Improvements in Mechanical Properties of Collagen-Based Scaffolds for Tissue Engineering. *Curr. Opin. Biomed. Eng.* 17:100253. https://linkinghub.elsevier.com/retrieve/pii/S2468451120300489.
7. Hapach, L. A., J. A. VanderBurgh, …, C. A. Reinhart-King. 2015. Manipulation of in Vitro Collagen Matrix Architecture for Scaffolds of Improved Physiological Relevance. *Phys. Biol.* 12:061002. https://iopscience.iop.org/article/10.1088/1478-3975/12/6/061002.
8. Sun, Y.-L., Z.-P. Luo, …, K.-N. An. 2002. Direct Quantification of the Flexibility of Type I Collagen Monomer. *Biochem. Biophys. Res. Commun.* 295:382–386. https://linkinghub.elsevier.com/retrieve/pii/S0006291X0200685X.
9. Rezaei, N., A. Lyons, and N. R. Forde. 2018. Environmentally Controlled Curvature of Single Collagen Proteins. *Biophys. J.* 115:1457–1469. https://linkinghub.elsevier.com/retrieve/pii/S0006349518310270.
10. Bodian, D. L., R. J. Radmer, …, T. E. Klein. 2011. Molecular Dynamics Simulations of the Full Triple Helical Region of Collagen Type I Provide an Atomic Scale View of the Protein's Regional Heterogeneity. *Pac. Symp. Biocomput.* 193–204. http://www.worldscientific.com/doi/abs/10.1142/9789814335058_0021.
11. in't Veld, P. J., and M. J. Stevens. 2008. Simulation of the Mechanical Strength of a Single Collagen Molecule. *Biophys. J.* 95:33–39. https://linkinghub.elsevier.com/retrieve/pii/S0006349508702820.
12. Saitoh, K.-I., T. Sato, …, Y. Takahashi. 2020. Molecular Dynamics Study of Collagen Fibrils: Relation between Mechanical Properties and Molecular Chirality. *J. Biomaterials Nanobiotechnol.* 11:260–278. https://www.scirp.org/journal/doi.aspx?doi=10.4236/jbnb.2020.114017.
13. Hartmann, J., and M. Zacharias. 2021. Mechanism of Collagen Folding Propagation Studied by Molecular Dynamics Simulations. *PLoS Comput. Biol.* 17:e1009079. https://dx.plos.org/10.1371/journal.pcbi.1009079.
14. Zapp, C., A. Obarska-Kosinska, …, F. Gräter. 2020. Mechanoradicals in Tensed Tendon Collagen as a Source of Oxidative Stress. *Nat. Commun.* 11:2315. https://www.nature.com/articles/s41467-020-15567-4.
15. Rowe, J., and K. Röder. 2023. Chemical Bonds in Collagen Rupture Selectively under Tensile Stress. *Phys. Chem. Chem. Phys.* 25:2331–2341. https://xlink.rsc.org/?DOI=D2CP05051J.
16. Depalle, B., Z. Qin, …, M. J. Buehler. 2015. Influence of Cross-Link Structure, Density and Mechanical Properties in the Mesoscale Deformation Mechanisms of Collagen Fibrils. *J. Mech. Behav. Biomed. Mater.* 52:1–13. https://linkinghub.elsevier.com/retrieve/pii/S175161611400201X.
17. Tavakol, M., and T. J. Vaughan. 2023. A Coarse-Grained Molecular Dynamics Investigation of the Role of Mineral Arrangement on the Mechanical Properties of Mineralized Collagen Fibrils. *J. R. Soc. Interface.* 20:20220803. https://royalsocietypublishing.org/doi/10.1098/rsif.2022.0803.
18. Gautieri, A., A. Russo, …, M. J. Buehler. 2010. Coarse-Grained Model of Collagen Molecules Using an Extended MARTINI Force Field. *J. Chem. Theor. Comput.* 6:1210–1218. https://pubs.acs.org/doi/10.1021/ct100015v.
19. Souza, P. C. T., R. Alessandri, …, S. J. Marrink. 2021. Martini 3: A General Purpose Force Field for Coarse-Grained Molecular Dynamics. *Nat. Methods.* 18:382–388. https://www.nature.com/articles/s41592-021-01098-3.
20. Souza, P. C. T., L. Borges-Araújo, …, S. Thallmair. 2025. GōMartini 3: From Large Conformational Changes in Proteins to Environmental Bias Corrections. Preprint at bioRxiv. http://biorxiv.org/lookup/doi/10.1101/2024.04.15.589479.
21. Monego, D., M. Brosz, …, F. Gräter. 2025. ColBuilder: Flexible Structure Generation of Crosslinked Collagen Fibrils. *Bioinformatics.* 41:btaf278. https://academic.oup.com/bioinformatics/article/doi/10.1093/bioinformatics/btaf278/8125020.
22. Brosz, M., N. Michelarakis, …, F. Gräter. 2022. Martini 3 Coarse-Grained Force Field for Poly(Para-Phenylene Ethynylene)s. *Phys.*

Chem. Chem. Phys. 24:9998–10010. https://xlink.rsc.org/?DOI=D1CP04237H.

23. Orgel, J. P. R. O., T. C. Irving, …, T. J. Wess. 2006. Microfibrillar Structure of Type I Collagen in Situ. *Proc. Natl. Acad. Sci. USA.* 103:9001–9005. https://pnas.org/doi/full/10.1073/pnas.0502718103.
24. Best, R. B., and G. Hummer. 2009. Optimized Molecular Dynamics Force Fields Applied to the Helix−Coil Transition of Polypeptides. *J. Phys. Chem. B.* 113:9004–9015. https://pubs.acs.org/doi/10.1021/jp901540t.
25. Lindorff-Larsen, K., S. Piana, …, D. E. Shaw. 2010. Improved Side-chain Torsion Potentials for the Amber ff99SB Protein Force Field. *Proteins.* 78:1950–1958. https://onlinelibrary.wiley.com/doi/10.1002/prot.22711.
26. Jorgensen, W. L., J. Chandrasekhar, …, M. L. Klein. 1983. Comparison of Simple Potential Functions for Simulating Liquid Water. *J. Chem. Phys.* 79:926–935. https://pubs.aip.org/jcp/article/79/2/926/776316/Comparison-of-simple-potential-functions-for.
27. Durell, S. R., B. R. Brooks, and A. Ben-Naim. 1994. Solvent-Induced Forces between Two Hydrophilic Groups. *J. Phys. Chem.* 98:2198–2202. https://pubs.acs.org/doi/abs/10.1021/j100059a038.
28. Beglov, D., and B. Roux. 1994. Finite Representation of an Infinite Bulk System: Solvent Boundary Potential for Computer Simulations. *J. Chem. Phys.* 100:9050–9063. https://pubs.aip.org/jcp/article/100/12/9050/473082/Finite-representation-of-an-infinite-bulk-system.
29. Huang, J., and A. D. MacKerell. 2013. CHARMM36 All-Atom Additive Protein Force Field: Validation Based on Comparison to NMR Data. *J. Comput. Chem.* 34:2135–2145. https://onlinelibrary.wiley.com/doi/10.1002/jcc.23354.
30. Lee, C., W. Yang, and R. G. Parr. 1988. Development of the Colle-Salvetti Correlation-Energy Formula into a Functional of the Electron Density. *Phys. Rev. B Condens. Matter.* 37:785–789. https://link.aps.org/doi/10.1103/PhysRevB.37.785.
31. Sousa Da Silva, A. W., and W. F. Vranken. 2012. ACPYPE - AnteChamber PYthon Parser interfacE. *BMC Res. Notes.* 5:367. https://bmcresnotes.biomedcentral.com/articles/10.1186/1756-0500-5-367.
32. Abraham, M., A. Alekseenko, …, E. Lindahl. 2023. GROMACS 2023 Manual. https://zenodo.org/record/7588711.
33. Jung, J., C. Kobayashi, …, Y. Sugita. 2021. New Parallel Computing Algorithm of Molecular Dynamics for Extremely Huge Scale Biological Systems. *J. Comput. Chem.* 42:231–241. https://onlinelibrary.wiley.com/doi/10.1002/jcc.26450.
34. Berendsen, H. J. C., D. Van Der Spoel, and R. Van Drunen. 1995. GROMACS: A Message-Passing Parallel Molecular Dynamics Implementation. *Comput. Phys. Commun.* 91:43–56. https://linkinghub.elsevier.com/retrieve/pii/001046559500042E.
35. Van Der Spoel, D., E. Lindahl, …, H. J. C. Berendsen. 2005. GROMACS: Fast, Flexible, and Free. *J. Comput. Chem.* 26:1701–1718. https://onlinelibrary.wiley.com/doi/10.1002/jcc.20291.
36. Abraham, M. J., T. Murtola, …, E. Lindahl. 2015. GROMACS: High Performance Molecular Simulations through Multi-Level Parallelism from Laptops to Supercomputers. *SoftwareX.* 1–2:19–25. https://linkinghub.elsevier.com/retrieve/pii/S2352711015000059.
37. Páll, S., and B. Hess. 2013. A Flexible Algorithm for Calculating Pair Interactions on SIMD Architectures. *Comput. Phys. Commun.* 184:2641–2650. https://linkinghub.elsevier.com/retrieve/pii/S0010465513001975.
38. Darden, T., D. York, and L. Pedersen. 1993. Particle Mesh Ewald: An Nlog(N) Method for Ewald Sums in Large Systems. *J. Chem. Phys.* 98:10089–10092. https://pubs.aip.org/jcp/article/98/12/10089/461765/Particle-mesh-Ewald-An-N-log-N-method-for-Ewald.
39. Essmann, U., L. Perera, …, L. G. Pedersen. 1995. A Smooth Particle Mesh Ewald Method. *J. Chem. Phys.* 103:8577–8593. https://pubs.aip.org/jcp/article/103/19/8577/180219/A-smooth-particle-mesh-Ewald-method.
40. Parrinello, M., and A. Rahman. 1981. Polymorphic Transitions in Single Crystals: A New Molecular Dynamics Method. *J. Appl. Phys.* 52:7182–7190. https://pubs.aip.org/jap/article/52/12/7182/9558/Polymorphic-transitions-in-single-crystals-A-new.
41. Martyna, G. J., D. J. Tobias, and M. L. Klein. 1994. Constant Pressure Molecular Dynamics Algorithms. *J. Chem. Phys.* 101:4177–4189. https://pubs.aip.org/jcp/article/101/5/4177/165484/Constant-pressure-molecular-dynamics-algorithms.
42. Jung, J., and Y. Sugita. 2020. Group-Based Evaluation of Temperature and Pressure for Molecular Dynamics Simulation with a Large Time Step. *J. Chem. Phys.* 153:234115. https://pubs.aip.org/jcp/article/153/23/234115/199662/Group-based-evaluation-of-temperature-and-pressure.
43. Kutzner, C., J. Czub, and H. Grubmüller. 2011. Keep It Flexible: Driving Macromolecular Rotary Motions in Atomistic Simulations with GROMACS. *J. Chem. Theor. Comput.* 7:1381–1393. https://pubs.acs.org/doi/10.1021/ct100666v.
44. Alessandri, R., J. Barnoud, …, S. J. Marrink. 2022. Martini 3 Coarse-Grained Force Field: Small Molecules. *Adv. Theory Simul.* 5:2100391. https://onlinelibrary.wiley.com/doi/10.1002/adts.202100391.
45. Işık, M., T. D. Bergazin, …, D. L. Mobley. 2020. Assessing the Accuracy of Octanol–Water Partition Coefficient Predictions in the SAMPL6 Part II Log P Challenge. *J. Comput. Aided Mol. Des.* 34:335–370. http://link.springer.com/10.1007/s10822-020-00295-0.
46. Crooks, G. E. 1998. Nonequilibrium Measurements of Free Energy Differences for Microscopically Reversible Markovian Systems. *J. Stat. Phys.* 90:1481–1487. http://link.springer.com/10.1023/A:1023208217925.
47. Shirts, M. R., E. Bair, …, V. S. Pande. 2003. Equilibrium Free Energies from Nonequilibrium Measurements Using Maximum-Likelihood Methods. *Phys. Rev. Lett.* 91:140601. https://link.aps.org/doi/10.1103/PhysRevLett.91.140601.
48. Brooks, B. R., C. L. Brooks, …, M. Karplus. 2009. CHARMM: The Biomolecular Simulation Program. *J. Comput. Chem.* 30:1545–1614. https://onlinelibrary.wiley.com/doi/10.1002/jcc.21287.
49. Lee, J., X. Cheng, …, W. Im. 2016. CHARMM-GUI Input Generator for NAMD, GROMACS, AMBER, OpenMM, and CHARMM/OpenMM Simulations Using the CHARMM36 Additive Force Field. *J. Chem. Theor. Comput.* 12:405–413. https://pubs.acs.org/doi/10.1021/acs.jctc.5b00935.
50. Kirkwood, J. G. 1935. Statistical Mechanics of Fluid Mixtures. *J. Chem. Phys.* 3:300–313. https://pubs.aip.org/jcp/article/3/5/300/203696/Statistical-Mechanics-of-Fluid-Mixtures.
51. Berendsen, H. J. C., J. P. M. Postma, …, J. R. Haak. 1984. Molecular Dynamics with Coupling to an External Bath. *J. Chem. Phys.* 81:3684–3690. https://pubs.aip.org/jcp/article/81/8/3684/565473/Molecular-dynamics-with-coupling-to-an-external.
52. Goga, N., A. J. Rzepiela, …, H. J. C. Berendsen. 2012. Efficient Algorithms for Langevin and DPD Dynamics. *J. Chem. Theor. Comput.* 8:3637–3649. https://pubs.acs.org/doi/10.1021/ct3000876.
53. Bernetti, M., and G. Bussi. 2020. Pressure Control Using Stochastic Cell Rescaling. *J. Chem. Phys.* 153:114107. https://pubs.aip.org/jcp/article/153/11/114107/199610/Pressure-control-using-stochastic-cell-rescaling.
54. Gapsys, V., S. Michielssens, …, B. L. De Groot. 2015. Pmx: Automated Protein Structure and Topology Generation for Alchemical Perturbations. *J. Comput. Chem.* 36:348–354. https://onlinelibrary.wiley.com/doi/10.1002/jcc.23804.
55. Seeliger, D., and B. L. De Groot. 2010. Protein Thermostability Calculations Using Alchemical Free Energy Simulations. *Biophys. J.* 98:2309–2316. https://linkinghub.elsevier.com/retrieve/pii/S000634951000216X.
56. Neese, F. 2025. Software Update: The ORCA Program System—Version 6.0. *WIREs Comput. Mol. Sci.* 15:e70019. https://wires.onlinelibrary.wiley.com/doi/10.1002/wcms.70019.
57. Marenich, A. V., C. J. Cramer, and D. G. Truhlar. 2009. Universal Solvation Model Based on Solute Electron Density and on a Continuum Model of the Solvent Defined by the Bulk Dielectric Constant and Atomic Surface Tensions. *J. Phys. Chem. B.* 113:6378–6396. https://pubs.acs.org/doi/10.1021/jp810292n.

58. Landrum, G., P. Tosco, …, J. Lehtivarjo. RDKit: Open-source Cheminformatics. https://www.rdkit.org.

59. Fan, S., B. I. Iorga, and O. Beckstein. 2020. Prediction of Octanol-Water Partition Coefficients for the SAMPL6-log*P* Molecules Using Molecular Dynamics Simulations with OPLS-AA, AMBER and CHARMM Force Fields. *J. Comput. Aided Mol. Des.* 34:543–560. http://link.springer.com/10.1007/s10822-019-00267-z.

60. Grünewald, F., M. H. Punt, …, S. J. Marrink. 2022. Martini 3 Coarse-Grained Force Field for Carbohydrates. *J. Chem. Theor. Comput.* 18:7555–7569. https://pubs.acs.org/doi/10.1021/acs.jctc.2c00757.

61. Rühle, V., C. Junghans, …, D. Andrienko. 2009. Versatile Object-Oriented Toolkit for Coarse-Graining Applications. *J. Chem. Theor. Comput.* 5:3211–3223. https://pubs.acs.org/doi/10.1021/ct900369w.

62. Ramachandran, G. N., and G. Kartha. 1955. Structure of Collagen. *Nature.* 176:593–595. https://www.nature.com/articles/176593a0.

63. Ramachandran, G. N., and V. Sasisekharan. 1961. Structure of Collagen. *Nature.* 190:1004–1005. https://www.nature.com/articles/1901004a0.

64. Rich, A., and F. Crick. 1961. The Molecular Structure of Collagen. *J. Mol. Biol.* 3:483–506. https://linkinghub.elsevier.com/retrieve/pii/S0022283661800168.

65. Rich, A., and F. H. C. Crick. 1955. The Structure of Collagen. *Nature.* 176:915–916. https://www.nature.com/articles/176915a0.

66. Borges-Araújo, L., G. P. Pereira, …, P. C. Souza. 2024. Assessing the Martini 3 Protein Model: A Review of Its Path and Potential. *Biochim. Biophys. Acta Proteins Proteom.* 1872:141014. https://linkinghub.elsevier.com/retrieve/pii/S1570963924000219.

67. Poma, A. B., M. Cieplak, and P. E. Theodorakis. 2017. Combining the MARTINI and Structure-Based Coarse-Grained Approaches for the Molecular Dynamics Studies of Conformational Transitions in Proteins. *J. Chem. Theor. Comput.* 13:1366–1374. https://pubs.acs.org/doi/10.1021/acs.jctc.6b00986.

68. Marrink, S. J., H. J. Risselada, …, A. H. De Vries. 2007. The MARTINI Force Field: Coarse Grained Model for Biomolecular Simulations. *J. Phys. Chem. B.* 111:7812–7824. https://pubs.acs.org/doi/10.1021/jp071097f.

69. Kim, H., B. Fábián, and G. Hummer. 2023. Neighbor List Artifacts in Molecular Dynamics Simulations. *J. Chem. Theor. Comput.* 19:8919–8929. https://pubs.acs.org/doi/10.1021/acs.jctc.3c00777.

70. Marrink, S. J., and D. P. Tieleman. 2013. Perspective on the Martini Model. *Chem. Soc. Rev.* 42:6801–6822. https://xlink.rsc.org/?DOI=c3cs60093a.

71. Gowers, R., M. Linke, …, O. Beckstein. 2019. MDAnalysis: A Python Package for the Rapid Analysis of Molecular Dynamics Simulations, pp. 98–105. https://doi.curvenote.com/10.25080/Majora-629e541a-00e.

72. Michaud-Agrawal, N., E. J. Denning, …, O. Beckstein. 2011. MDAnalysis: A Toolkit for the Analysis of Molecular Dynamics Simulations. *J. Comput. Chem.* 32:2319–2327. https://onlinelibrary.wiley.com/doi/10.1002/jcc.21787.

73. Bergazin, T. D., N. Tielker, …, D. L. Mobley. 2021. Evaluation of Log P, pKa, and Log D Predictions from the SAMPL7 Blind Challenge. *J. Comput. Aided Mol. Des.* 35:771–802. https://link.springer.com/10.1007/s10822-021-00397-3.

74. Alessandri, R., F. Grünewald, and S. J. Marrink. 2021. The Martini Model in Materials Science. *Adv. Mater.* 33:2008635. https://onlinelibrary.wiley.com/doi/10.1002/adma.202008635.

75. Cowan, P. M., S. McGavin, and A. C. T. North. 1955. The Polypeptide Chain Configuration of Collagen. *Nature.* 176:1062–1064. https://www.nature.com/articles/1761062a0.

76. Shoulders, M. D., and R. T. Raines. 2009. Collagen Structure and Stability. *Annu. Rev. Biochem.* 78:929–958. https://www.annualreviews.org/doi/10.1146/annurev.biochem.77.032207.120833.

77. Kmiecik, S., D. Gront, …, A. Kolinski. 2016. Coarse-Grained Protein Models and Their Applications. *Chem. Rev.* 116:7898–7936. https://pubs.acs.org/doi/10.1021/acs.chemrev.6b00163.

78. Cao, F., S. von Bülow, …, K. Lindorff-Larsen. 2024. A Coarse-Grained Model for Disordered and Multi-Domain Proteins. *Protein Sci.* 33:e5172.

79. Rennekamp, B., H. Grubmüller, and F. Gräter. 2024. Hidden Length Lets Collagen Buffer Mechanical and Chemical Stress. *Phys. Rev. E.* 110:054408. https://link.aps.org/doi/10.1103/PhysRevE.110.054408.

80. Rennekamp, B., C. Karfusehr, …, F. Gräter. 2023. Collagen Breaks at Weak Sacrificial Bonds Taming Its Mechanoradicals. *Nat. Commun.* 14:2075. https://www.nature.com/articles/s41467-023-37726-z.

Biophysical Journal, Volume *125*

Supplemental information

Coarse-grained Martini 3 model for collagen fibrils

Matthias Brosz, Johanna Buck, Fabian Grünewald, Debora Monego, Jaewoon Jung, Yuji Sugita, Camilo Aponte-Santamaría, and Frauke Gräter

Supplemental Information: Coarse-Grained Martini 3 Model for Collagen Fibrils

Table S1: **Overview of the simulated collagen systems**: the single triple helix, the divalent and trivalent crosslinks, the 67 nm- and 335 nm-long fibril. We show the structure length in nm, the number of triple helices (3 alpha chains), number of crosslinks, number of D-bands, number of particles (excluding water molecules), and the simulation time of a single replicate.

Property	Triple helix	Free energy simulations (Crosslinks)	PYD	HLKNL	Short fibril	Long fibril
Length [nm]	67	-	-	-	67	335
Triple helix	1	-	-	-	41	225
Crosslinks	-	1	1	1	16	248
D-band	1	-	-	-	1	5
Particles AA	8571	-	60	44	73 k	4.9 Mio
Particles CG	1940	-	11	5	323 k	1.1 Mio
Simulation time AA [ns]	100 /segment	4×(100× forward/ backward), 2–10 ns each	100	100	100	450
Simulation time CG [ns]	100 /segment	1x(100× forward/ backward), 2–10 ns each	100	100	100	300

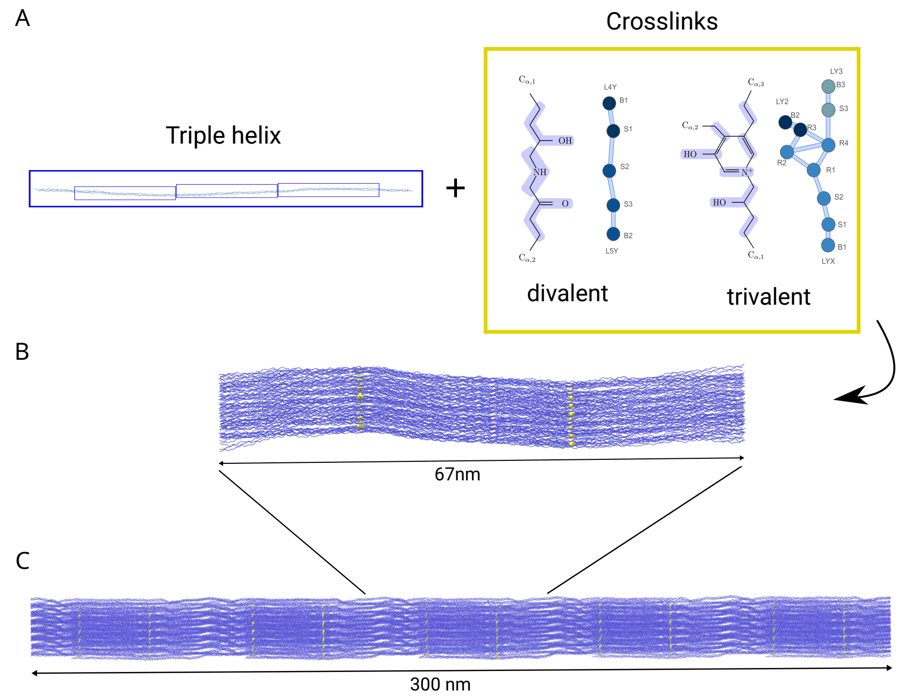

Figure S1: **Overview of collagen systems simulated for parametrization.** **A** Schematic showing triple helix building blocks (*left*) combined with divalent and trivalent crosslinks (*right, yellow box*). **B** 67 nm-long fibril containing 40 triple helices with crosslinks at 2 regions (*yellow dots*). **C** 335 nm-long fibril containing 267 triple helices with crosslinks at 10 regions (*yellow dots*). The three independently simulated 67 nm-long triple helix segments without crosslinks (highlighted rectangles in panel **A**) were used for backbone parametrization, while the fibrillar systems (**B** and **C**) were used to parametrize and validate crosslink interactions and mechanical properties.

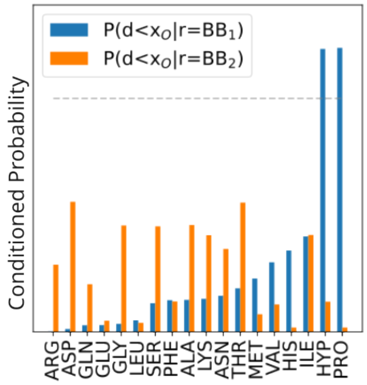

Figure S2: **Residue-specific bond length distributions in triple helix parametrization.** Conditional probability of specific amino acids at the start of bonds (BB_1, given that the bond length (d) is shorter than the intersection point (x_0) of the bimodal gaussian distribution, from equilibrium distributions. This analysis focuses on the first peak of the bimodal distribution ($d < x_0$), revealing that proline and hydroxyproline have very high probability of being involved in shorter bonds. Based on these results, bonds involving proline-type amino acids were assigned optimized bond lengths and force constants.

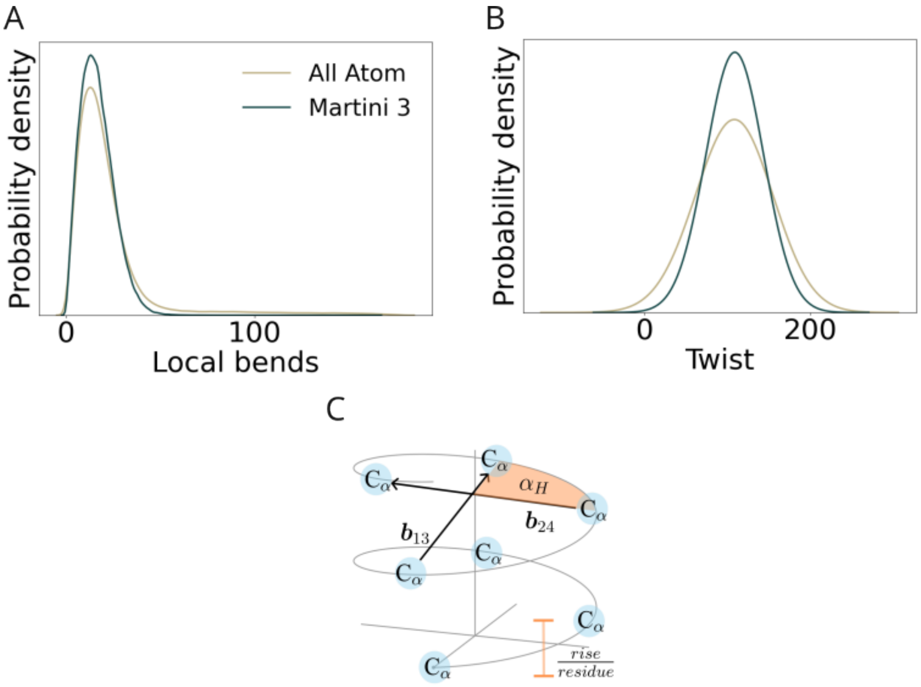

Figure S3: **Triple helical shape parameters.** **A** Local bending angles between helical axes separated by three residues, comparing AA (*brown*) and CG (*gray*) simulations. **B** Helical twist angle distributions showing good agreement between AA and CG models. **C** Schematic illustration of helical geometry calculations. For twist calculations, four adjacent C_α-atoms are selected and connected in sequence by Euclidean vectors to determine bisectors b_{13} and b_{24} (*black arrows*). The helical twist α_H is calculated as the dot product of these bisectors to estimate the enclosed angle. Local bending describes the deviation of local helical segments from perfect linearity. These geometric parameters validate that the CG model accurately reproduces the characteristic triple helical shape observed in AA reference simulations, confirming proper structural representation at the coarse-grained level.

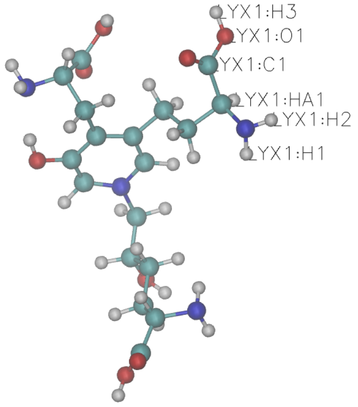

Figure S4: **Capping strategy for crosslink parametrization: Atomistic structure of the PYD crosslink.** The trivalent PYD crosslink connects two chains from one triple helix to one chain from another triple helix, requiring capping of three terminal ends for parametrization. To avoid using AMBER force field ACE/NME caps that would alter partitioning behavior, CHARMM36m force field was selected for free energy calculations since it provides suitable standard capping without additional modifications. Using the *pdb2gmx* command with CHARMM36m, each peptide bond terminus was capped: the amide group with a single hydrogen (-NH_2) and the carboxylate group with another (-COOH). This capping strategy preserves the crosslink's native chemical environment while enabling accurate thermodynamic property calculations for CG parametrization. The labeled atoms (LYX1:H3, LYX1:O1, etc.) indicate the CHARMM36m naming convention used in the calculations.

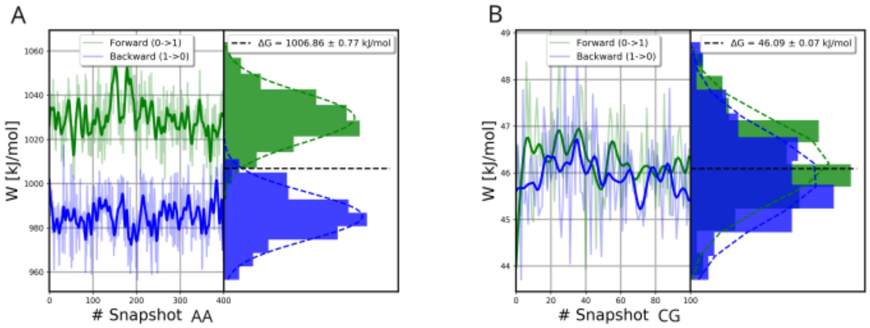

Figure S5: **Work distributions from non-equilibrium MD-based free energy calculations for crosslink parametrization. A** All-atom simulations of the divalent crosslink showing 400 forward (0→1, *green*) and 400 backward (1→0, *blue*) transitions between water and octanol phases. **B** Corresponding CG-MD simulations with 100 forward and 100 backward transitions. The work distributions (*left*) and cumulative work plots (*right*) demonstrate the sampling quality for free energy estimation using Crooks fluctuation theorem. The calculated free energy differences (ΔG values shown) validate the thermodynamic consistency between AA and CG models. This non-equilibrium approach enabled iterative optimization of bead types and mapping schemes to match the partitioning behavior of crosslinks between our CG model and AA reference simulations, ensuring accurate representation of crosslink hydrophobic/hydrophilic character in the CG model.

Free energy estimates and logP value comparison

QM(SMD) calculations

For the QM calculation we used the QM-software Orca 6.0 [9, 8] with the B3LYP DFT functional with the DEF2-SVP basis set, we performed a geometry optimization and computed vibrational frequencies numerically (finite differences of energies) using Grimme's D4 dispersion correction. These settings were also suggested in the manual of ORCA 6.0 [9, 10]. For the solvent, we chose to use the inexpensive solvent type SMD [5] with the specification *water* or *1-octanol*.

Thermodynamic integration for the CG *wet* calculations

To obtain a logP value in a solvated octanol configuration, we chose to perform DTI - thermodynamic integration using the same sampling as for the method presented for AA and CG *dry* methods described in detail in the main text. We applied the relation of 920:80 (octanol:water) [2] as solvent for *wet* octanol representing the experimental conditions which were used to fit the ML data. We first solvated the box with octanol+water or water, followed by an energy minimization, nvt and npt equilibration and the thermodynamic integration. We followed Martini3 standard methods found in [6]. We modified this approach using lambda in steps of 0.05 and a longer simulation time of 50 ns for each lambda value. After the integration, the free energy of solvation was computed

via the GROMACS bar module which uses Bennett acceptance ratio (BAR).

Using AlogP with SMILES

For calculating AlogP, we used the virtual computational chemistry tool which is available online using a SMILES as an input. Further details can be found in the respective publications of the tool [12].

Using RDKit with SMILES

We also applied the machine learned predicting tool RDKit on the SMILES of both crosslinks and obtained further values for comparison [4].

Crosslink	CG *wet*	QM(SMD)	AlogP	RDKit
HLKNL	-3.50 ± 0.69	-2.9	-4.2	-2.1
PYD	-5.46 ± 0.76	-4.3	-4.2	-5.5

Table S2: Comparison of logP values across different predicting methods. The AlogP values are of the same magnitude which does not correspond to our expectations. Due to the charge of the PYD molecule we expected a higher logP value than for HLKNL. The RDKit value, however, shows agreement with the trend and also the value for the PYD crosslink agrees (when including the Cl in the SMILES).

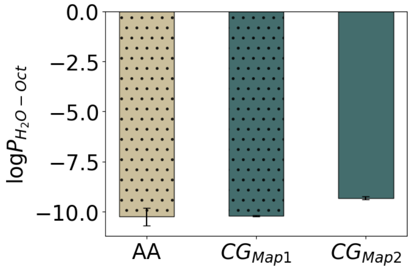

Label	Map 1 (chosen)	Map 2 (alternative)
B1	SP2	SP2
S1	TC3	TC4
S2	SP1	SP1
R1	TQ3p	TQ2p
R2	TP1q	TP1q
R4	TC5q	TC6q
R3	TC5	TC4
S3	TC3	TC4
B2	SP2	SP2
B3	SP2	SP2

Figure S6: **Bead type optimization through octanol/water partitioning validation.** Comparison of partition coefficients ($logP_{H_2O} \rightarrow Oct$) for the trivalent crosslinks using different Martini 3 bead types at specific backbone connection sites. Initially, $TC3$ beads were selected following standard Martini 3 protocols for positions $S1$, $S3$ of the trivalent crosslink and for $R3$ a $TC5$ bead (see Fig. 2A). However, comparison with AA reference simulations showed that $TC4$ beads provide better agreement at those positions (e.g., AA: 10.26, CG ($Map1$): 10.22 and CG ($Map2$): 9.33). This iterative optimization process of trying out many different alternative mappings where we show one example here ensured that the CG model accurately reproduces the thermodynamic properties of crosslinks in different solvent environments, validating the final bead type selection for the crosslink parametrization.

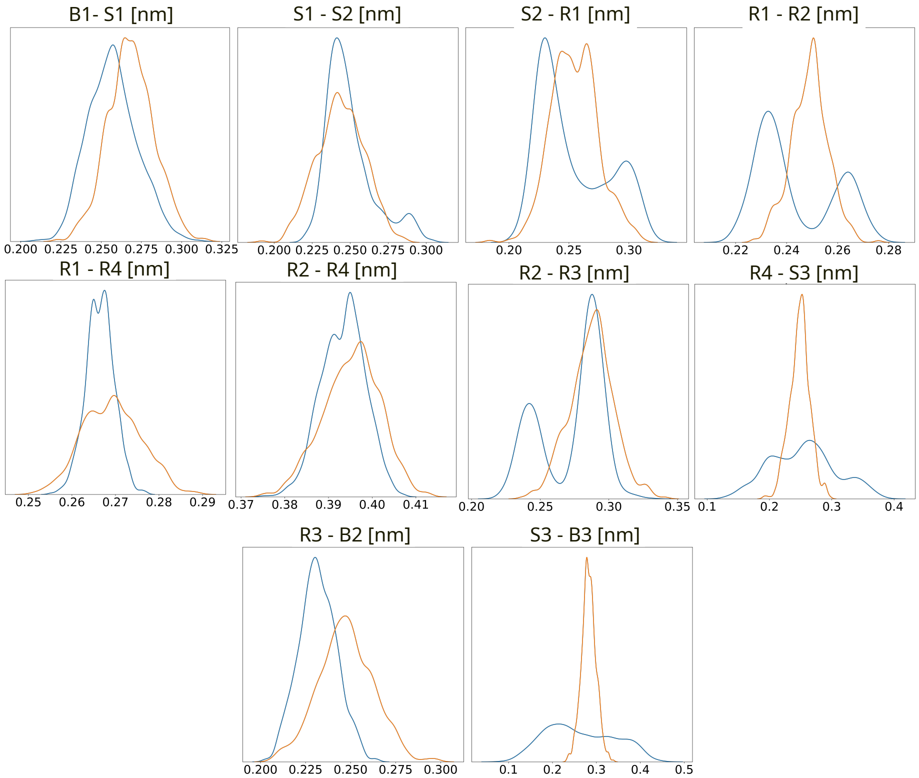

Figure S7: **Bond length distributions showing SASA-based optimization validation.** Probability densities for all bonded terms in the trivalent crosslink comparing center-of-geometry mapped AA trajectories (*blue*) with SASA-optimized Martini 3 simulations (*orange*). Each panel shows a different bond pair within the crosslink structure (*B1-S1*, *S1-S2*, *S2-R1*, etc, referring to the bead labels in Fig. 2A). The SASA-based optimization procedure adjusted bond lengths to better reproduce the structural characteristics observed in AA simulations, accounting for the different effective sizes of atoms versus CG beads. Without SASA-based adjustments, the CG distributions would have identical means to the initial Boltzmann inversion by construction, but would not accurately capture the true structural flexibility. The improved agreement between AA and CG distributions after SASA optimization demonstrates that this approach successfully maintains that crosslink's structural integrity while preserving its dynamic behavior in the CG representation.

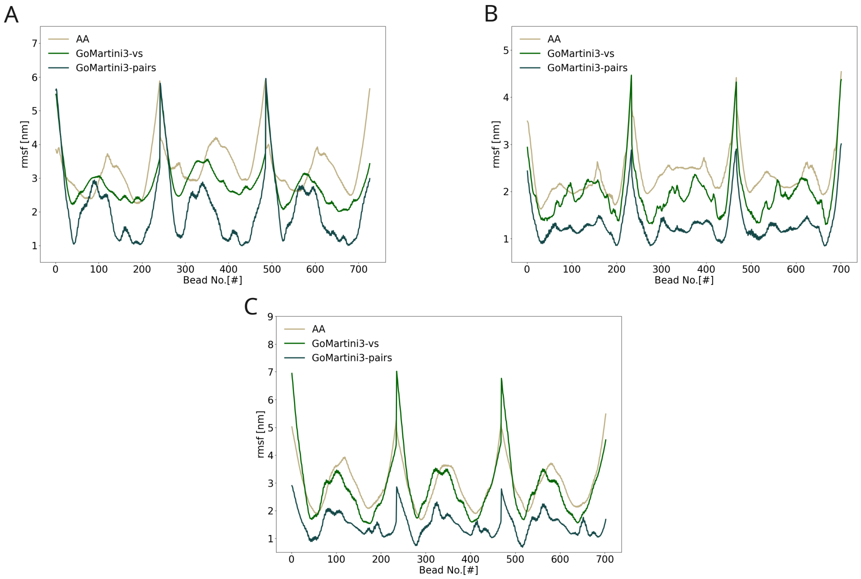

Figure S8: **Gō model implementation comparison: Root-mean-square-fluctuations for virtual sites versus pairwise interactions.** RMSF profiles comparing AA reference (*brown*), standard GōMartini 3 with virtual sites (*green*), and our modified GōMartini 3 with pairwise interactions (*blue*) across three different collagen molecules segments (**A, B, C**). The standard "vs" approach uses virtual sites for Gō model interactions following default GōMartini 3 protocols, while our "pairs" approach defines interactions directly between bead pairs. Although both CG approaches reproduce the overall magnitude of AA fluctuations, the pairwise method shows reduced noise and improved stability compared to virtual sites. Importantly, the pairwise approach dramatically reduces memory requirements, making it computationally feasible for large fibrillar systems where the virtual site implementation becomes impractical due to excessive memory demands. The three panels represent different regions along the collagen molecule, demonstrating consistent performance across the entire structure.

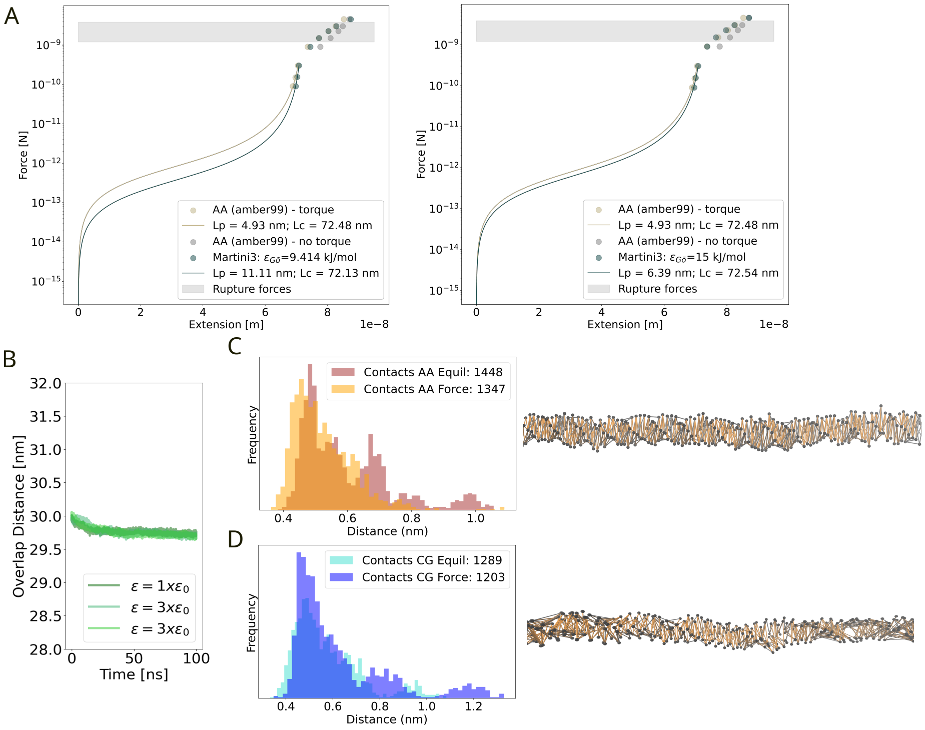

Figure S9: **Gō model details: Influence of the potential well depth** ϵ **A** End-to-end distance of the triple helix for different forces observed from AA and CG simulations. Also, the influence of the torque restrains, applied to both ends of the collagen triple helix, for the AA simulations is shown. **B** Overlap length of a trivalently crosslinked fibril with varying ϵ values (1x, 3x, 5x the original value of $\epsilon_{G\bar{o}} = 9.414\,\mathrm{kJ\,mol^{-1}}$). **C** Gō model contacts under equilibrium and under force shown as a distribution of distances and vizualised in an example triple helix under force. The number of contacts shown is measured with a threshold of 1.1 nm which corresponds to the Lennard-Jones cutoff. Orange contacts persist under force while gray contacts are below the tresshold.

In our force-extension analysis, we observe two different regimes for this semiflexible polymer under tension: Entropic regime at low forces: The Gō model epsilon significantly affects the force-extension behavior, with the default value ($\epsilon_{G\bar{o}} = 9.414\,\mathrm{kJ\,mol^{-1}}$) providing good agreement with AA simulations. Enthalpic regime at high force, $> 300\,\mathrm{pN}$: Under high pulling-forces, collagen's mechanical response to stress is driven by stretching stiffness as the chain becomes stretched and larger than its persistence length. In this regime, the Gō model has minimal influence because it is not designed to handle such high forces. Both Gō model parameter sets converge to similar behavior, and additional tests with scaled epsilon values (factors of 3, 5, shown in the right panel of the figure) confirm that changes to the Gō model parameter have negligible impact on the high-force stretching response. As we see through stretching also the contacts below the Lennard-Jones cutoff of 1.1 nm is reduced. As expected, the distances of Gō contacts also shift to higher values.

Topology generation of crosslinked Martini 3 collagen fibrils

To generate the topology of the collagen fibril, we used a bottom-up approach by first constructing individual collagen molecule topologies, and second merging them based on crosslinking information. For each single collagen molecule, we pre-processed the coordinate file and used the *martinize2* command from the *vermouth* package to generate the Martini 3 force field topology and coordinate file [3]. We then applied the *contact map* analysis script to identify close contacts in the atomistic structure, considering both geometric and chemical criteria, namely interatomic distances and amino acid types. Using the Martini 3 coordinate file and the atomistic *contact map* as inputs for the *create_goVirt.py* script from Poma, we generated the GōMartini 3 topology and coordinates files by combining physics-based and structure-based modeling approaches[11]. This pipeline was applied to each triple helix to account for their intrahelical interactions. To combine crosslinked triple helices, we used the *connect* file from ColBuilder, which enables generation of fibrillar structures[7]. Next, crosslinks were incorporated at sites selected based on experimental knowledge of lysine/hydroxylysine positions as defined in ColBuilder. We then combined the topology files of crosslinked collagen molecules and added the bonded potentials between crosslink beads. We modified the standard GōMartini 3 approach by defining Gō-like Lennard-Jones interactions between virtual sites as *pairs*, rather than assigning new bead types for each virtual site. Using a cut-off of 1.1 nm, this modification enabled efficient generation of multi-million particle systems using *grompp* from GROMACS (v2023)[1]. The original definition of virtual site interactions in GōMartini 3 is impractical with *grompp* due to excessive memory and time requirements: We tested the time required to compile different sized test system (table below) to extract at which size the pairs approach is needed. Of note, this virtual site memory issue persists even with newer versions of GROMACS when simulating large systems. For fibrillar structure which were the aim in this work, we were not able to compile with gmx grompp within sensible timescales using current standard RAM. The final topology combined the GōMartini 3 files for pairs and triplets of collagen triple helices with the bonded terms for divalent and trivalent crosslinks.

We tested a compliation of an energy minimization at the start of the simulation without any solvent beads. The limit was measured on a standard Laptop with 31GB RAM GROMACS version 2022.5. The same result was also obtained with a interactive node of 32GB memory and GROMACS version 2024.3. Importantly, the Gō model needs to be of high density because of collagens given density and the triplehelical structure which we want to preserve. Table S3 shows why the decision was necessary to switch to pairs in order to simulate at scale.

System (no water)	No. of virtual sites	Virtual sites, t	Pairs, t
Triple helix	702	16 s	9 s
30 nm long fibril, dc = 18	14k	$> 7h$	29 s
40 nm long fibril, dc = 18	18k	fails	31 s
67 nm long fibril	26k	fails	37 s
300 nm long fibril, dc =40	393k	fails	231 s

Table S3: Overview of systems and the compilation time of the Gō model approaches virtual sites and pairs.

Equilibration procedure for Martini 3 collagen fibrils

Dependent on the collagen system, we performed a multi-step equilibration procedure to enable production run simulations. The 67 nm-long crosslinked collagen fibril was equilibrated in the NVT ensemble for 5 ns and for 30 ns with the NPT ensemble using a $2-5$ fs timestep.

For the 335 nm collagen fibril, we applied a multi-step equilibration procedure as given in Fig. S10. Moreover, we observed water holes subsequent to our pulling simulations. Thus, we introduced a resolvation step before we continued with further pulling, where we inserted more water molecules and performed shorter NPT-equilibration steps in between. We then continued with the pulling simulations where we first performed first constant velocity pulling in order to let the fibril adapt to the force, followed by the production run under constant force. Every pull group consisted of the three caps at the end of a single triple helix. We pulled with 3 nN per triple helix, thus 1 nN per strand. In the case of the 335 nm-long collagen fibril, this accumulates to 606 nN at each side.

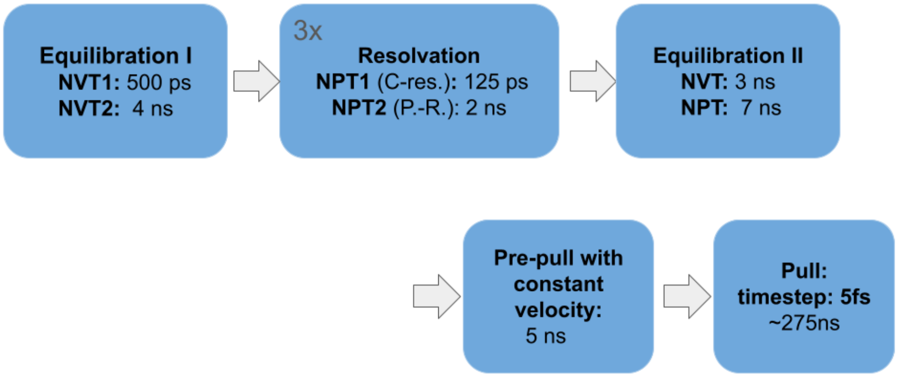

Figure S10: **Equilibration procedure for the 335 nm-long fibril.** Flowchart showing the five-step equilibration protocol used for large fibrillar systems, with timescales and ensemble conditions for each stage.

Martini force field parameters

Table S4: Martini 3 force field bonded terms of the protein backbone beads

Bond/Angle	Value	Force Constants
BB(all amino acids except proline/hydroxyproline)- BB	0.356 nm	18000 kJ mol^{-1} nm^{-2}
BB(proline/hydroxyproline)- BB	0.320 nm	34000 kJ mol^{-1} nm^{-2}
epsilon of Gō potential	–	9.414 kJ mol^{-1}
BB_1-BB_2-BB_3	138°	152 kJ mol^{-1}
BB_1-BB_2-BB_3-BB_4	76°	17 kJ mol^{-1}

Table S5: Martini 3 force field bonded terms of the trivalent PYD crosslink.

Bond/Angle	Value	Force Constants
B1-S1	0.270 nm	18000 kJ mol^{-1} nm^{-2}
S1-S2	0.250 nm	15000 kJ mol^{-1} nm^{-2}
S2-R1	0.260 nm	14000 kJ mol^{-1} nm^{-2}
R1-R2	0.250 nm	72000 kJ mol^{-1} nm^{-2}
R1-R4	0.270 nm	72000 kJ mol^{-1} nm^{-2}
R2-R4	0.395 nm	68000 kJ mol^{-1} nm^{-2}
R3-R4	0.370 nm	12000 kJ mol^{-1} nm^{-2}
R2-R3	0.230 nm	12000 kJ mol^{-1} nm^{-2}
R4-S3	0.290 nm	11000 kJ mol^{-1} nm^{-2}
R3-B2	0.250 nm	18000 kJ mol^{-1} nm^{-2}
S3-B3	0.250 nm	18000 kJ mol^{-1} nm^{-2}
B1-S1-S2	180°	150 kJ mol^{-1}
S1-S2-R1	180°	150 kJ mol^{-1}
S2-R1-R2	180°	150 kJ mol^{-1}
S2-R1-R4	100°	200 kJ mol^{-1}
R2-R3-B2	140°	150 kJ mol^{-1}
R4-S3-B3	140°	150 kJ mol^{-1}

Table S6: Martini 3 force field bonded terms of the divalent crosslink.

**Bond/Angle**	**Value**	**Force Constants**
B1-S1	0.310 nm	10000 $kJ\,mol^{-1}\,nm^{-2}$
S1-S2	0.415 nm	7000 $kJ\,mol^{-1}\,nm^{-2}$
S2-S3	0.365 nm	5000 $kJ\,mol^{-1}\,nm^{-2}$
S3-B2	0.360 nm	9000 $kJ\,mol^{-1}\,nm^{-2}$
B2-S3-S2	100°	150 $kJ\,mol^{-1}$
S1-S2-S3	140°	150 $kJ\,mol^{-1}$
B1-S1-S2	140°	150 $kJ\,mol^{-1}$

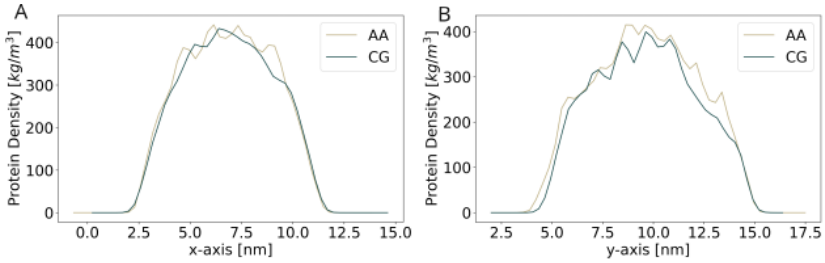

Figure S11: **Lateral spacing in the fibrillar model.** Comparison of protein density along the x- and y-axes from AA CG simulations of a 67 nm stretched model, averaged over the final 20 ns. Water is excluded. The close agreement in lateral density profiles indicates similar diameters and lateral packing under applied force.

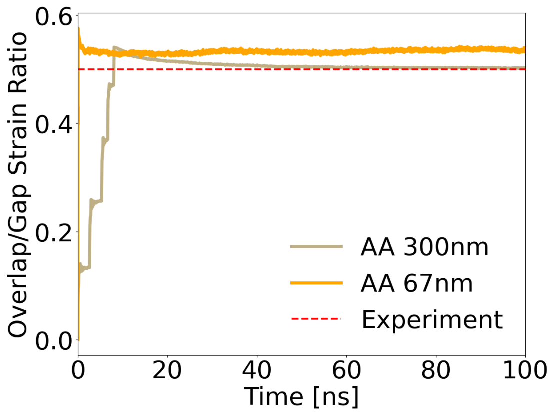

Figure S12: **Boundary effects in fibrillar models of different lengths.** Overlap/gap strain ratio comparing 67 nm vs. 335 nm all-atom fibril models under applied force, with experimental reference value (*red dashed line*. The smaller 67 nm fibril shows significantly higher strain ratios due to boundary effects, where the limited system size constraints natural deformation patterns. The longer 335 nm fibril exhibits strain ratios closer to the experimental value, demonstrating that boundary effects are minimized in larger systems. This comparison shows the need for sufficiently large fibrillar models to accurately capture physiological mechanical behavior and avoid artifacts from finite size effects.

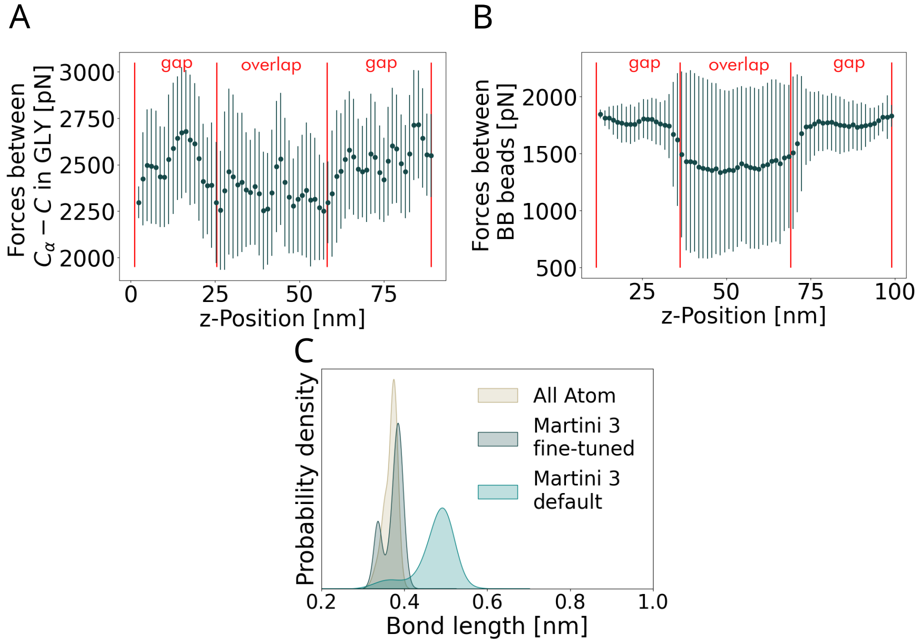

Figure S14: **Force distribution analysis of divalent crosslinked 67 nm fibril under force. A)** C_α-C bond forces in glycine residues from all-atom simulations, projected along the fibril axis and averaged over the final 70 ns. **B)** Backbone bead forces from Martini 3 simulations under applied force, showing similar periodicity. Both panels demonstrate the characteristic gap/overlap pattern with red vertical lines marking structural transitions. **C** Bond length distributions comparing all-atom reference with fine-tuned and default Martini 3 parameters, validating the improved structural representation. The force profiles reveal that divalent crosslinks produce mechanical behavior very similar to trivalent crosslinks (see Figure 4B), with lower forces in overlap regions and higher forces in gap regions. This analysis confirms that the coarse-grained model accurately reproduces the heterogeneous stress distribution along the fibril length observed in all-atom simulations.

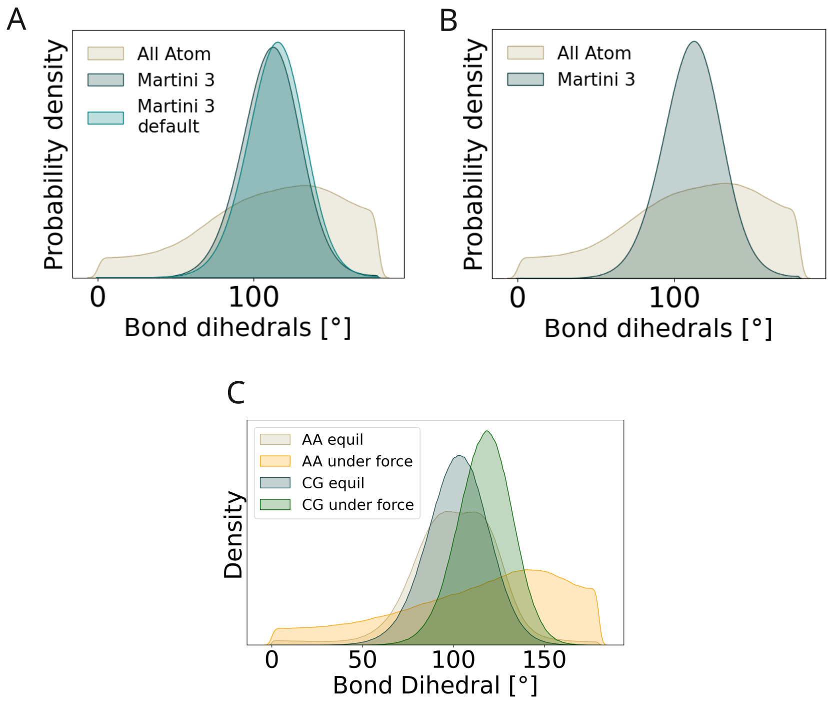

Figure S13: **Dihedral angle distributions comparing equilibrium and force conditions.** Backbone bead dihedral angle for **A)** 67 nm fibril with trivalent crosslinks, **B)** 67 nm fibril with divalent crosslinks, and **C)** 67 nm triple helix under equilibrium versus applied force. The AA data was mapped to CG resolution. Under force, both AA and CG models show rightward peak shifts, indicating structural deformation. However, AA simulations exhibit much broader angular distributions under force, covering nearly all possible angles, while CG models maintain more restricted Gaussian distributions. This difference reflects the fundamental limitation of CG dihedral parametrization: dihedrals are fitted under equilibrium conditions and cannot fully capture the extreme flexibility observed in AA simulations under force. However, as demonstrated in the main results, this does not compromise the model's ability to accurately reproduce key mechanical properties such as force-extension behavior and fibril elongation.

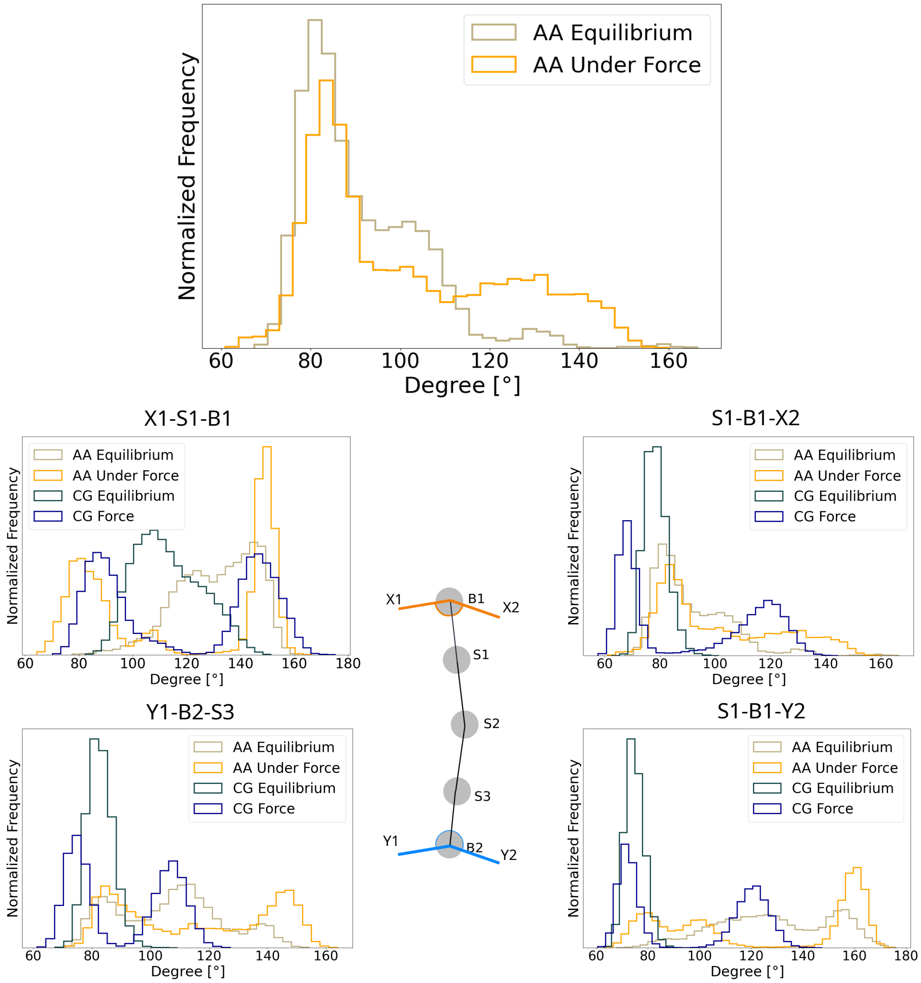

Figure S15: **Crosslink-protein backbone bond angle distributions from the 67 nm-long divalently crosslinked collagen fibril.** Overall bond angle distributions from AA simulations showing wide-spread angles under equilibrium conditions (*gray, left*) and under 1 nN pulling force (*orange, left*). Due to the wide distribution which is shown in the AA distribution, we decided to avoid tighter constraints in these regions due to the risk of overfitting to uncertain or non-representative geometries. Comparison of four specific crosslink-backbone angles (*X1-B1-S1*, *S1-B1-X2*, *Y1-B2-S3*, *S3-B2-Y2*) between AA and CG simulations under equilibrium and force conditions (*right*). The bond angle labels correspond to the crosslink mapping scheme (see Fig. 2A). Good agreement between AA and CG distributions validates the crosslink parametrization, with both models showing similar angle distributions under equilibrium and the expected narrowing of angular flexibility under applied force.

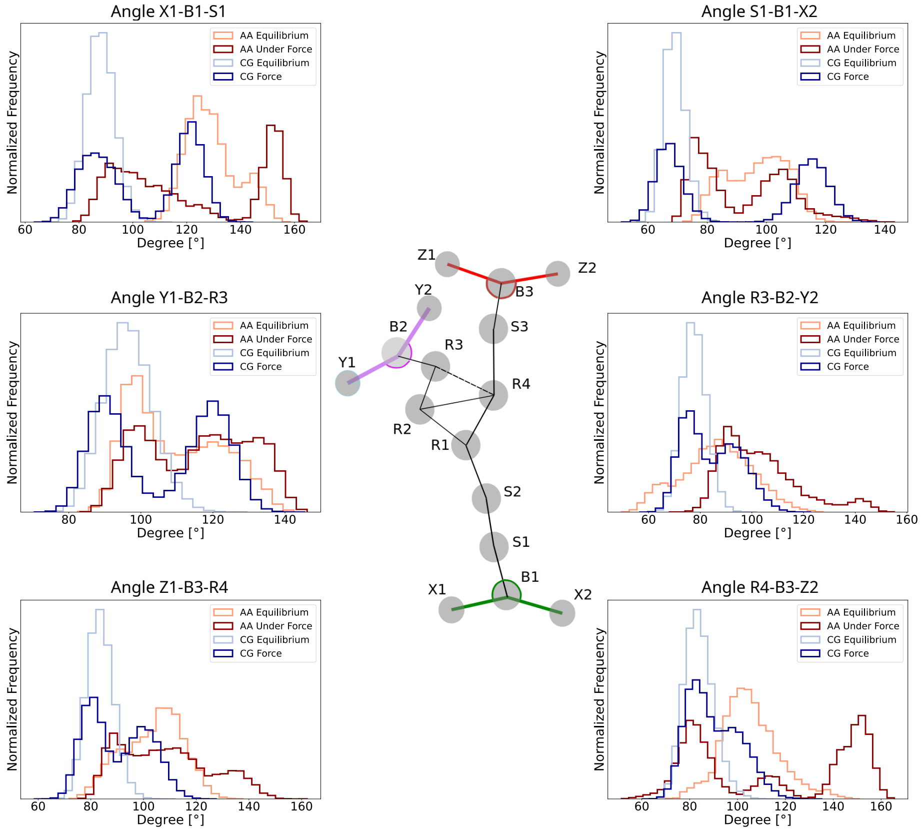

Figure S16: **Crosslink-protein backbone bond angle distributions from the 67 nm-long trivalently crosslinked collagen fibril.** Schematic of the trivalent crosslink structure showing the six bond angles analyzed (*X1-B1-S1*, *S1-B1-X2*, *Y1-B2-R3*, *R3-B2-Y2*, *Z1-B3-R4*, *R4-B3-Z2*) (*center*). Comparison of the AA and CG bond angle distributions between the protein backbone and the trivalent crosslink under equilibrium and force conditions. Overall, the CG data captures the dynamics of the bond angles reasonably well, with good agreement between AA and CG models for most angles.

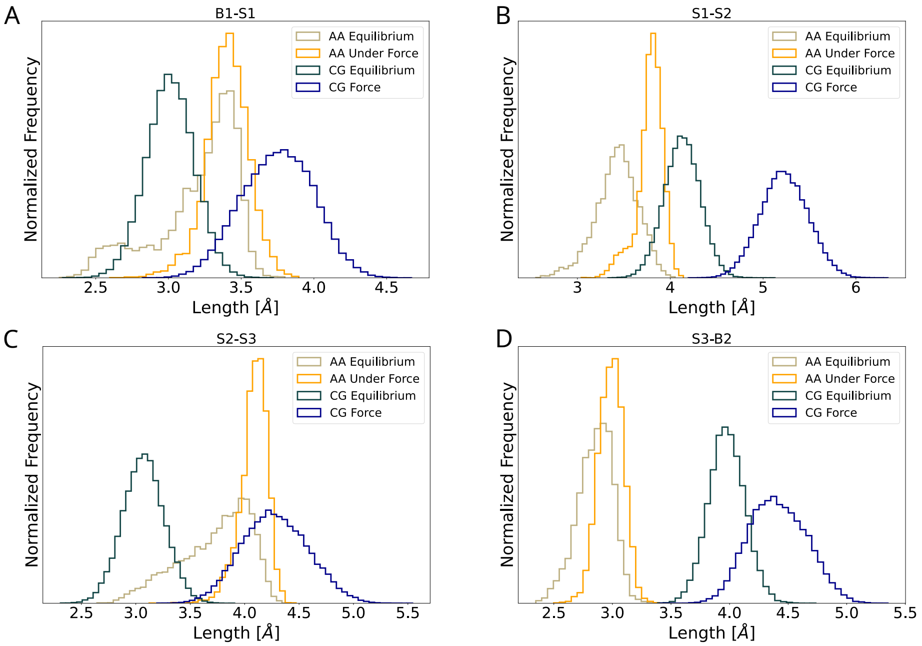

Figure S17: **Crosslink bond length distributions from the 67 nm divalently crosslinked collagen fibril. A-D** Divalent crosslink bond length distributions for specific bond pairs (*B1-S1*, *S2-S3*, *S3-B2*) comparing equilibrium simulations with simulations under applied force (1000 pN). Bond labels correspond to the divalent crosslink mapping scheme (see Fig. 2A). We observe similar trends between CG and AA reference simulations under both equilibrium and force conditions, with both models showing characteristic bond extension under applied force. However, slight deviations in peak positions and distribution widths are visible, reflecting the coarse-grained approximation while maintaining overall structural consistency.

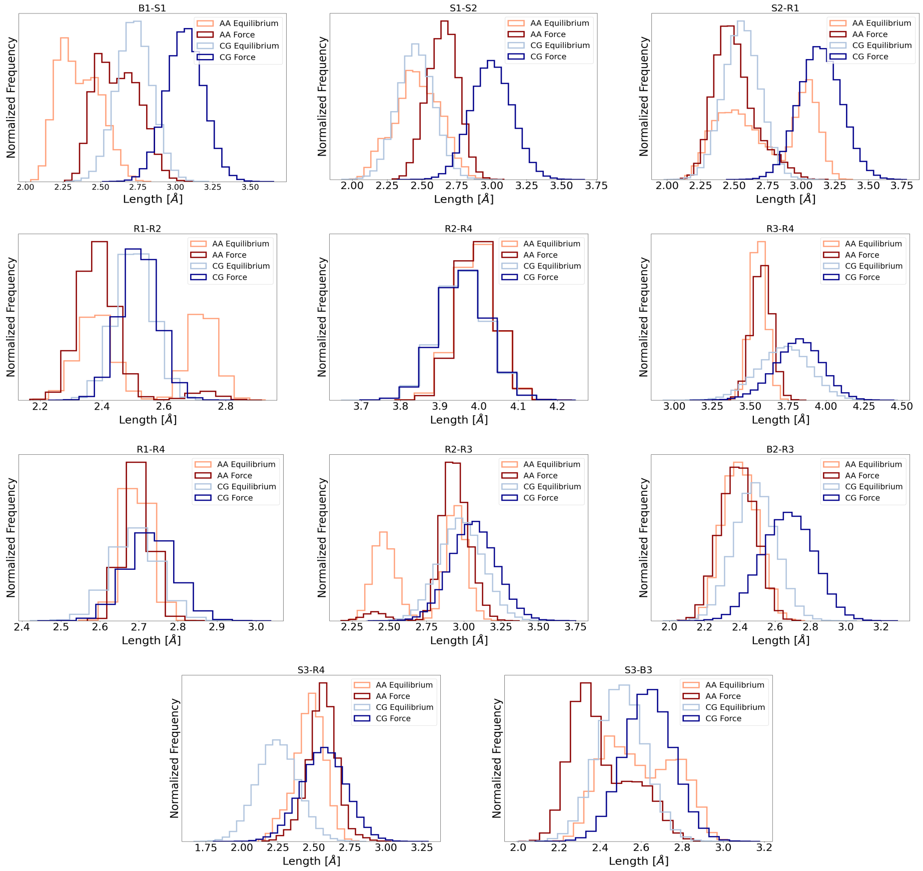

Figure S18: **Crosslink bond length distributions from the 67 nm trivalently crosslinked collagen fibril.** Trivalent crosslink bond length distributions for specific bond pairs (*B1-S1*, *S1-S2*,*S2-R1*, *R1-R2*, *R2-R4*, *R3-R4*, *R1-R4*, *R2-R3*, *S3-R4*, *S1-B3*) comparing equilibrium simulations with simulations under applied force (1000 pN). Bond labels correspond to the trivalent crosslink mapping scheme (see Fig. 2A). We observe similar trends between CG and AA reference simulations, with characteristic bond extension under applied force. However, slight deviations are visible, particularly for bonds involving the aromatic ring structure (*R1-R4*, *R2-R4*, *R3-R4*), reflecting the increased complexity of accurately representing the rigid pyridine ring geometry in the coarse-grained model.

References

[1] M. Abraham, A. Alekseenko, C. Bergh, C. Blau, E. Briand, M. Doijade, S. Fleischmann, V. Gapsys, Gaurav Garg, S. Gorelov, G. Gouaillardet, A. Gray, M. Eric Irrgang, F. Jalalypour, J. Jordan, C. Junghans, Prashanth Kanduri, S. Keller, C. Kutzner, J. A. Lemkul, M. Lundborg, P. Merz, V. Miletić, D. Morozov, S. Páll, R. Schulz, M. Shirts, A. Shvetsov, B. Soproni, D. Van Der Spoel, P. Turner, C. Uphoff, A. Villa, S. Wingbermühle, A. Zhmurov, P. Bauer, B. Hess, and E. Lindahl. GROMACS 2023 Manual. doi: 10.5281/ZENODO.7588711. URL https://zenodo.org/record/7588711.

[2] F. Grünewald, M. H. Punt, E. E. Jefferys, P. A. Vainikka, M. König, V. Virtanen, T. A. Meyer, W. Pezeshkian, A. J. Gormley, M. Karonen, M. S. P. Sansom, P. C. T. Souza, and S. J. Marrink. Martini 3 Coarse-Grained Force Field for Carbohydrates. 18(12):7555–7569. ISSN 1549-9618, 1549-9626. doi: 10.1021/acs.jctc.2c00757. URL https://pubs.acs.org/doi/10.1021/acs.jctc.2c00757.

[3] P. C. Kroon, F. Grünewald, J. Barnoud, M. van Tilburg, P. C. T. Souza, T. A. Wassenaar, and S.-J. Marrink. Martinize2 and Vermouth: Unified Framework for Topology Generation. URL https://arxiv.org/abs/2212.01191.

[4] G. Landrum, P. Tosco, B. Kelley, R. Rodriguez, D. Cosgrove, R. Vianello, sriniker, P. Gedeck, G. Jones, E. Kawashima, NadineSchneider, D. Nealschneider, A. Dalke, tadhurst cdd, M. Swain, B. Cole, S. Turk, A. Savelev, A. Vaucher, M. Wójcikowski, I. Take, H. Faara, R. Walker, V. F. Scalfani, D. Probst, K. Ujihara, N. Maeder, A. Pahl, G. Godin, and J. Lehtivarjo. RDKit: Open-source cheminformatics. https://www.rdkit.org. URL https://www.rdkit.org.

[5] A. V. Marenich, C. J. Cramer, and D. G. Truhlar. Universal Solvation Model Based on Solute Electron Density and on a Continuum Model of the Solvent Defined by the Bulk Dielectric Constant and Atomic Surface Tensions. 113(18):6378–6396. ISSN 1520-6106, 1520-5207. doi: 10.1021/jp810292n. URL https://pubs.acs.org/doi/10.1021/jp810292n.

[6] Martini Development Team. Free energy techniques tutorial. Martini Force Field Documentation. URL https://cgmartini.nl/docs/tutorials/Martini3/Free_Energy_Techniques/index.html.

[7] D. Monego, M. Brosz, J. Buck, V. Viliuga, P. Greicius, J. Jung, T. Stuehn, M. Schmies, Y. Sugita, and F. Gräter. ColBuilder: Flexible structure generation of crosslinked collagen fibrils. 41(6). ISSN 1367-4811. doi: 10.1093/bioinformatics/btaf278. URL https://academic.oup.com/bioinformatics/article/doi/10.1093/bioinformatics/btaf278/8125020.

[8] F. Neese. Software Update: The ORCA Program System—Version 6.0. 15 (2):e70019. ISSN 1759-0876, 1759-0884. doi: 10.1002/wcms.70019. URL https://wires.onlinelibrary.wiley.com/doi/10.1002/wcms.70019.

[9] F. Neese, F. Wennmohs, U. Becker, and C. Riplinger. The ORCA quantum chemistry program package. 152(22):224108, . ISSN 0021-9606, 1089-7690. doi: 10.1063/5.0004608. URL https://pubs.aip.org/jcp/article/152/22/224108/1061982/The-ORCA-quantum-chemistry-program-package.

[10] F. Neese, F. Wennmohs, U. Becker, and C. Riplinger. *ORCA 6.0 Manual*, . URL https://www.faccts.de/docs/orca/6.0/manual/.

[11] A. B. Poma, M. Cieplak, and P. E. Theodorakis. Combining the MARTINI and Structure-Based Coarse-Grained Approaches for the Molecular Dynamics Studies of Conformational Transitions in Proteins. 13(3):1366–1374. ISSN 1549-9618, 1549-9626. doi: 10.1021/acs.jctc.6b00986. URL https://pubs.acs.org/doi/10.1021/acs.jctc.6b00986.

[12] I. V. Tetko, J. Gasteiger, R. Todeschini, A. Mauri, D. Livingstone, P. Ertl, V. A. Palyulin, E. V. Radchenko, N. S. Zefirov, A. S. Makarenko, V. Y. Tanchuk, and V. V. Prokopenko. Virtual Computational Chemistry Laboratory – Design and Description. 19(6):453–463. ISSN 0920-654X, 1573-4951. doi: 10.1007/s10822-005-8694-y. URL http://link.springer.com/10.1007/s10822-005-8694-y.
